# *Arabidopsis thaliana* exhibits wide within‐species variation in tolerance to boron limitation and root and shoot trait resilience associate with a pleiotropic locus

**DOI:** 10.1111/nph.70570

**Published:** 2025-09-15

**Authors:** Thomas D. Alcock, Manuela Désirée Bienert, Astrid Junker, Rhonda C. Meyer, Henning Tschiersch, Sreelekha Kudamala, Nicolaus von Wirén, Thomas Altmann, Gerd Patrick Bienert

**Affiliations:** ^1^ Crop Physiology, TUM School of Life Sciences Technical University of Munich Alte Akademie 12 Freising Germany; ^2^ HEF World Agricultural Systems Center Technical University of Munich Liesel‐Beckmann‐Straße 2 Freising Germany; ^3^ Metalloid Transport Group Leibniz Institute of Plant Genetics and Crop Plant Research, OT Gatersleben Corrensstrasse 3 Seeland Germany; ^4^ Department of Molecular Genetics Leibniz Institute of Plant Genetics and Crop Plant Research, OT Gatersleben Corrensstrasse 3 Seeland Germany; ^5^ Department Physiology and Cell Biology Leibniz Institute of Plant Genetics and Crop Plant Research, OT Gatersleben Corrensstrasse 3 Seeland Germany

**Keywords:** *Arabidopsis thaliana* (thale cress), automatic phenotyping, boron, genome‐wide association study, natural variation, nutrient efficiency, pleiotropy, root phenotyping

## Abstract

- To improve plant tolerance to suboptimal availability of the micronutrient boron (B), it is crucial to understand the mechanisms plants have evolved to tolerate B‐limited conditions.
- We assessed temporal physiological, ionomic and molecular responses to B deficiency across 185 *Arabidopsis thaliana* accessions grown in soil‐substrate in an automated phenotyping system and on agar plates.
- Whilst profound shoot‐ and root‐growth inhibition was observed in most accessions under B limitation, seven highly B‐deficiency tolerant accessions with < 20% reduced fresh and digital biomass accumulation were identified. Boron‐efficient accessions were characterised by sustaining lateral more than primary root growth under B limitation. Whilst expression of B transporters increased under B limitation, no correlations between expression and B uptake or B efficiency were observed, suggesting increased B‐use efficiency in B‐efficient accessions. Phylogenetic analysis suggests B efficiency evolved independently multiple times in response to local environmental needs. Genome‐wide association analyses identified a QTL on chromosome 4 that is associated with both root and shoot resilience to B limitation.
- Our results suggest that an optimised root system contributes to maintaining shoot productivity in B‐limited conditions. Further dissection of the identified QTL and candidate genes will form an important strategy for elucidating the molecular control of B efficiency.

## Introduction

Boron (B) is an essential element for vascular plants (Lewis, 2020; Wimmer *et al*., 2020) which forms dimerizing di‐ester bonds between two rhamnogalacturonan II (RG‐II) monomers in the pectin fraction of the primary cell wall. The number of RG‐II dimers determines the integrity, plasticity and stability of plant cell walls (O'Neill *et al*., 2004). Hence, at the cellular level, B is biochemically important for determining cell wall properties, apoplastic transport and compartmentalisation processes, stabilisation of membranes, cell growth, cell differentiation, pollen development, pollen tube growth, mechanosensing and penetration resistance to pathogens. At the macroscopic level, B deficiency results in heart rot in roots and shoots, susceptibility to pathogens, impaired root and shoot meristem activity, morphological abnormalities such as stunting, leaf curling, wilting and leaf darkening and impaired flower development and fertility (Marschner, 2012). Together, these impairments lead to decreased plant performance and stress tolerance and ultimately reduced yield (Shorrocks, 1997; Eggert & von Wirén, 2013).

World‐wide, B is one of the most frequently deficient and actively managed micronutrients in crops, and B fertilisation is critical for achieving optimal agricultural yield and quality, even in cereals, which have a relatively low demand for B (Bienert *et al*., 2023). At their onset, B deficiency‐induced defects are typically phenotypically latent. Thus, reliable field‐suitable biomarkers for early B deficiency in crops are lacking, and B‐fertilisation measures are not always employed when most needed. The breeding of B‐efficient cultivars is crucial to sustainably improve the performance and fertility of crops grown under temporary suboptimal B conditions. To achieve this, it is necessary to understand physiological and molecular mechanisms underlying B efficiency in plants.

Various plant nutrient efficiency mechanisms were identified based on natural variation in the ionome of the model plant *Arabidopsis thaliana* (Arabidopsis; Rus *et al*., 2006; Loudet *et al*., 2007; Baxter *et al*., 2008, 2012; Kobayashi *et al*., 2008; Morrissey *et al*., 2009; Chao *et al*., 2012; Pineau *et al*., 2012; Koprivova *et al*., 2013; Campos *et al*., 2017). Characterisation of genes and proteins regulating B uptake and transport has also been achieved using Arabidopsis (Onuh & Miwa, 2021). Two transmembrane protein families are chiefly responsible for B uptake and translocation in Arabidopsis, namely the boron transporter (BOR) and Nodulin26‐like intrinsic protein (NIP) families. AtNIP5;1 is essential for the efficient uptake of B from the soil into the plant under B‐deficient growth conditions (Takano *et al*., 2006). AtBOR1 acts synergistically with AtNIP5;1 under low B supply and is required for loading B into the xylem in roots, and therefore for root‐to‐shoot B translocation (Takano *et al*., 2002, 2010). The unloading of B out of the xylem into shoot tissues is mediated by AtNIP6;1 (Tanaka *et al*., 2008). These pioneering discoveries in Arabidopsis paved the way for further studies advancing our understanding of B transport in other plant species (Matthes *et al*., 2020).

In contrast to our knowledge on B transport mechanisms, the genetic and molecular basis of B efficiency in Arabidopsis and plants in general is scarce. Genetic analyses of responses to B deficiency in Arabidopsis are restricted to a few studies considering just five different accessions (Zeng *et al*., 2007, 2008; Huai *et al*., 2018). Recently, AT1G78860 , encoding a curculin‐like lectin family protein, has been suggested to be the underlying gene for AtBE1‐2 (Huai *et al*., 2018), one of five described B‐efficiency quantitative trait loci (QTLs) in Arabidopsis (Zeng *et al*., 2008). How this protein can functionally impact B efficiency has not yet been resolved. Further genes and proteins determining B efficiency traits in Arabidopsis have yet to be identified. Successful approaches in *Brassica napus* (Zhang *et al*., 2014, 2024; Pommerrenig *et al*., 2018) indicate that a valuable prerequisite for the discovery of B‐efficiency QTLs is a detailed overview of natural variation in B efficiency amongst diverse accessions. This would additionally enable isolation of extreme accessions with contrasting B‐deficiency tolerance for use in fine‐mapping approaches. In combination with detailed sampling location meta‐data, as exist for many Arabidopsis accessions (The 1001 Genomes Consortium, 2016), this would also enable association of B efficiency within individual or groups of accessions with localised ancestral growth conditions, whilst enabling us to address the open question of whether B efficiency has evolved only once or multiple times via different mechanisms in Arabidopsis.

In this study, we comprehensively screened a genetically diverse collection of 185 Arabidopsis accessions from across the world to identify and characterise natural variation in temporal physiological, ionomic and molecular traits related to B‐deficiency tolerance. Different manual and automated phenotyping technologies and cultivation systems allowed us to unravel and characterise (1) B‐deficiency sensitive root and shoot growth traits, (2) morphological and molecular biomarkers for the detection of early B deficiency, (3) highly B deficiency‐tolerant and ‐sensitive accessions, (4) traits that correlate with B efficiency and most importantly (5) QTLs that are associated with root and shoot resilience to B limitation. The comparison of root and shoot system architecture and growth traits, elemental composition and B transporter transcript abundance among accessions grown under different exogenous B conditions, combined with analysis of phylogenetic relationships, suggested that independent B‐efficiency responses have evolved in individual and groups of Arabidopsis accessions.

## Materials and Methods

### Plant material

A total of 185 *Arabidopsis thaliana* (L.) Heynh. (Arabidopsis) accessions were selected based on their expected genotypic variation and diverse origin representing sites expected to vary in soil B availability (Supporting Information Table S1). *Atnip5;1* knock‐down mutant (SALK_122287) seeds were obtained from The European Arabidopsis Stock Centre (Loughborough, United Kingdom) and used as a control for B‐deficiency treatments.

### Plant cultivation on soil‐substrate in an automated plant phenotyping system

**Fig. 1:**
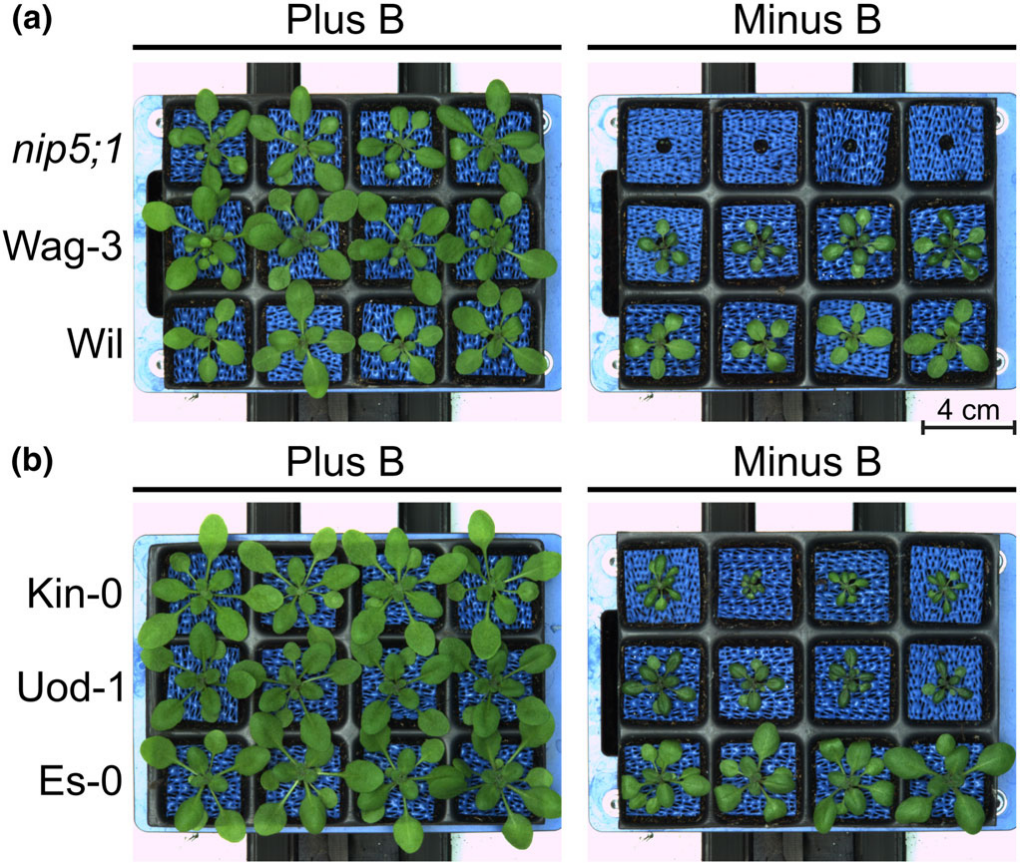
Visual appearance of shoots of selected Arabidopsis accessions contrasting in their boron (B) deficiency tolerance. Images of shoots of different Arabidopsis accessions were taken 20 d after sowing on B‐sufficient (2.5 mg B kg^−1^ soil) or B‐deficient (< 0.1 mg B kg^−1^ soil) soil. Each of the 12 growth units per tray are 4 × 4 cm. Blue mats under rosettes were used to increase contrast for automated imaging. The B transporter mutant *nip5;1*, which did not develop beyond germination under B‐deficient conditions in (a), served as a biological reference and demonstrates that the B‐deficient growth conditions in the phenotyping system were successfully established. Accessions in (b) represent examples of contrasting phenotypes with Es‐0 being a B‐efficient accession, Uod‐1 a B‐inefficient accession, and Kin‐0 a highly B‐inefficient accession. Wag‐3 and Wil (a) represent examples of B‐inefficient accessions.

Plants were cultivated in an automated plant transport and imaging system situated in a controlled environment plant growth chamber (Junker *et al*., 2015) set to long‐day (16 h day) conditions. Plant carriers consisted of 12 growth units of 4 × 4 cm (Fig. 1). The growth substrate used was a B‐free (< 0.1 mg B kg^−1^ substrate) white‐peat volcanic clay mixture, which was supplemented with 2.5 mg B kg^−1^ soil for the B‐replete treatment. Between 9 and 16 d after sowing (DAS), top view images of plants were taken daily in the visible range of the light spectrum and of static fluorescence signals as described previously (Junker *et al*., 2015). Shoots of a subset of 43 accessions selected for extreme contrasting B efficiency/inefficiency were additionally subjected to elemental analysis by ICP‐MS. Additional growth setup information and details related to data collection within this experimental setup can be found in Methods S1.

### Root phenotyping of *in vitro* cultivated plants

Square Petri dishes (120 × 120 × 17 mm, vented; Greiner) were used as growth containers for *in vitro* cultures. The experiment included the 185 selected accessions (Table S1) represented across five plants per Petri dish per accession and B treatment. Seeds were subjected to a pre‐culture for 5 d in media to which no B was added. Five seedlings per accession were then transferred to main‐culture media comprising a Phytagel (Sigma‐Aldrich Co. LLC)‐based modified half‐strength MS media with the same composition as the pre‐culture medium but pretreated with a B‐chelator then supplemented with 0.2 μM boric acid (B deficient) or 100 μM boric acid (B sufficient). Petri dishes were placed in a Percival Scientific CU chamber (CLF Climatics, Wertingen, Bavaria, Germany) with a photoperiod of 16 h light at 22°C (120 μmol m^−2^ s^−1^) and 8 h dark at 19°C. Petri dishes were scanned after 10 d of growth on main‐culture media; then, shoots were carefully separated from root systems for dry weight determination. Additionally, RNA was extracted from root systems of the same 43 accessions as selected for shoot elemental analysis to determine the expression of B‐transporter encoding genes. Additional growth setup information and details related to data collection within this experimental setup can be found in Methods S1.

### Genetic association and *in silico* parameter analysis

A maximum parsimony phylogenetic tree of the 185 Arabidopsis accessions was generated based on 214 051 Single Nucleotide Polymorphisms (SNPs; Atwell *et al*., 2010; Horton *et al*., 2012) using the R package phangorn (Schliep, 2011). The same SNP‐set, filtered to exclude SNPs with a minor allele frequency of 0.05, was also used to perform genome‐wide association (GWA) mapping, using the R package gapit (v.3) implementing the GWA method farmcpu (Liu *et al*., 2016). *k*‐means clustering was used to group accessions by shared phenotypic behaviour across 38 shoot and root trait ratios using the stats package in the software R. Additional details can be found in Methods S1.

## Results

### Vegetative growth of most Arabidopsis accessions is strongly limited by B limitation

**Fig. 2:**
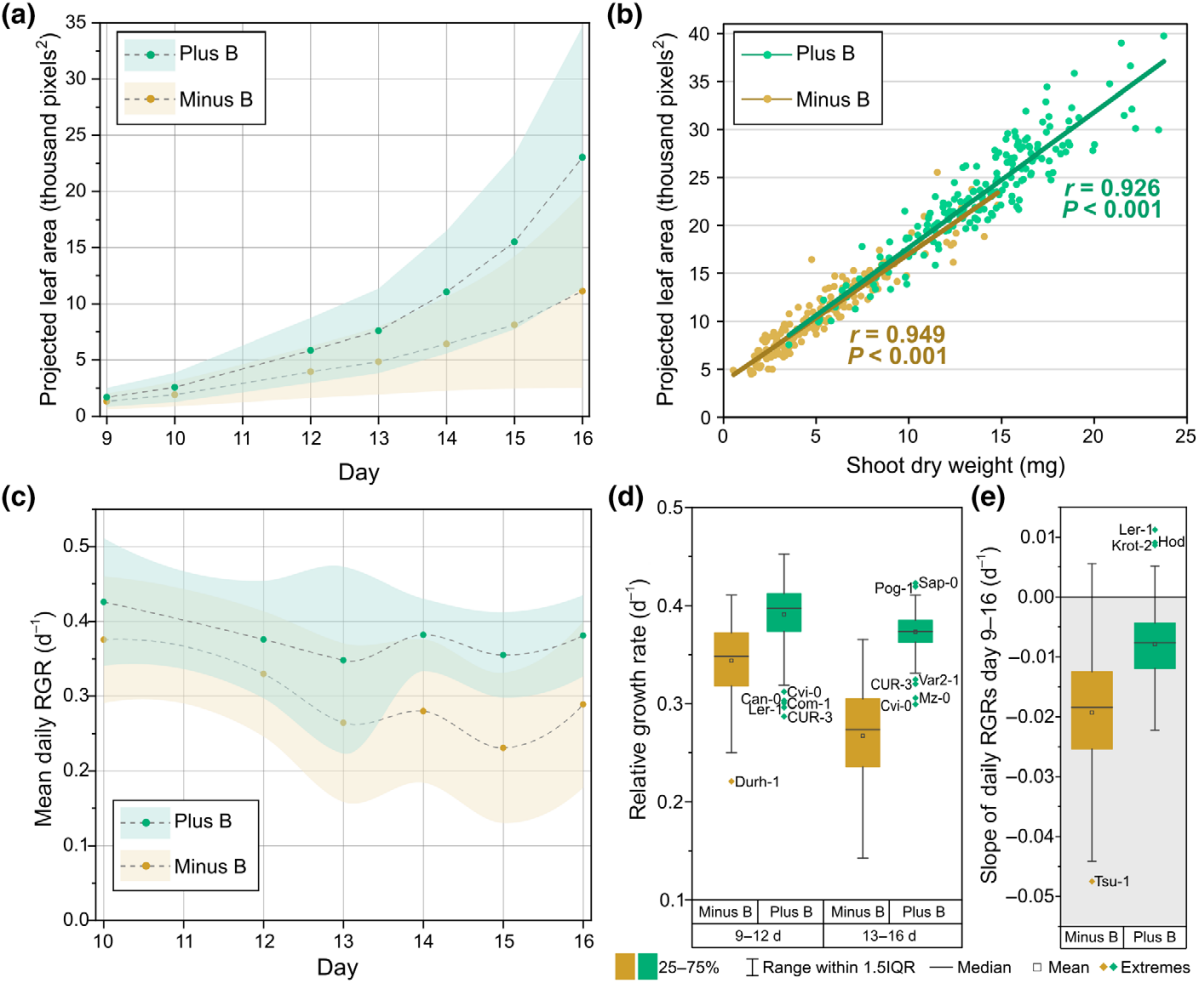
Growth parameters of Arabidopsis accessions grown in soil in an automated phenotyping experiment over time. Imaging data derived from measurements between 9 and 16 d after sowing using fluorescence imaging. (a) Mean average leaf area development of accessions over time. Dashed lines and dots represent mean average projected leaf area (PLA) across genotypes on each day in each boron (B) treatment. Shaded areas above and below lines represent SD. PLA between measurement days inferred with Akima Spline smoothing. (b) Correlation between shoot dry weight 20 d after sowing (DAS; experiment end) and PLA at day 16 (last imaging day) for both B‐sufficient (Plus B) and B‐deficient (Minus B) growth conditions. Values shown are Pearson correlation coefficients (*r*). (c) Change in daily relative growth rate (RGR) of accessions grown in either B‐sufficient or B‐deficient conditions. Dashed lines and dots represent mean average daily RGR across genotypes on each day in each B treatment. Shaded areas above and below lines represent SD. RGRs between measurement days inferred with Akima Spline smoothing. (d) RGRs in 3‐d intervals from 9–12 to 13–16 DAS across genotypes between B‐sufficient and B‐deficient conditions. (e) The slope of daily RGRs across accessions between B‐sufficient and B‐deficient conditions, where a more negative slope represents a faster reduction in RGR with time. For box plots in (d) and (e), boxes represent the 25^th^ to 75^th^ percentiles and whiskers represent the range within 1.5 times the interquartile range (IQR) with any extremes shown. The identities of extreme accessions are listed next to the corresponding data points.

Within the duration of the automated plant phenotyping system experiment, most of the 185 accessions showed typical B‐deficiency symptoms in the B‐deficient treatment (Fig. 1a,b). This was primarily visible as reduced shoot growth, lanceolated leaves, leaf curling and darker green leaf colour. The B transporter mutant *nip5;1* did not develop beyond germination under B‐deficient conditions (Fig. 1a), demonstrating that B‐deficient growth conditions in the phenotyping system were successfully established. A comparison between *nip5;1* and the accession Col‐0 from which it is derived is summarised in Fig. S1a. Of the 329 traits measured over the seven imaging days of the experiment (47 phenes per day), 94 were more than 20% higher or lower in B‐deficient compared to B‐sufficient growth conditions on average across accessions, indicating widespread disruption in response to B limitation. The majority of these (73) were apparent only from 13 DAS, which thus represents the time point at which seed B reserves and the limited available soil B were no longer enough to sustain unperturbed growth. Whilst most accessions continued to grow until harvest, plants subjected to B deficiency grew at a slower pace. This was reflected by the projected leaf area (PLA; Fig. 2a), which correlated strongly with shoot dry weight (DW) at 20 DAS (Figs 2b, S2) in both B‐sufficient (Pearson *r* = 0.926; *P* < 0.001) and B‐deficient (Pearson *r* = 0.949; *P* < 0.001) conditions. At day 16, PLAs of plants subjected to B sufficiency were on average 13.7 times larger than at day 9, while PLAs of plants subjected to B deficiency were only 8.5 times bigger (Fig. 2a). On average, accessions suffered a 22 and 52% reduction in PLA in B‐deficient compared to B‐sufficient conditions by Days 9 and 16, respectively (Table S1), with differences between B treatments becoming particularly clear after *c*. 12 DAS when the PLA was on average 32% lower in B‐deficient compared to B‐sufficient plants (Fig. 2a). The reference accession Col‐0 appeared particularly sensitive to B deficiency, suffering a 71% reduction in PLA by day 16 under B‐deficient compared to B‐sufficient conditions. Shoot fresh weight (FW) of plants at harvest (20 DAS) was on average 62% lower in plants subjected to B‐deficient compared to B‐sufficient conditions. Shoot FW of Col‐0 plants was on average 80% lower in B‐deficient compared to B‐sufficient conditions. Relative SD from replicate plants for each genotype for traits measured in the automated plant phenotyping system experiment averaged 25.0% for plants grown in B‐deficient conditions and 23.3% for plants grown in B‐sufficient conditions, indicating similar variability between replicates for each treatment (Table S2).

Relative growth rates (RGRs) of accessions were calculated on a daily (Fig. 2c) and a 3‐d (Fig. 2d) basis. Daily RGRs generally decreased between 9 and 16 DAS across accessions in both B conditions, but at a quicker rate under B‐deficient conditions (Fig. 2e). Under B‐sufficient conditions, RGR decreased by 10.6% between 10 and 16 DAS, but RGR decreased by 23.1% over the same period under B‐deficient conditions, on average. A clearly lower daily RGR under B‐deficient conditions was apparent across accessions from 14 DAS, when it was 27% lower than in B‐sufficient conditions (Fig. 2c). Similar effects were observed for the 3‐d RGRs, with a bigger difference in RGR between B treatments observed in the second half of the imaging period (13–16 DAS; Fig. 2d). Only three accessions, Sei‐0, Yo‐0 and Kn‐0, were in the 80^th^ percentile (37 top performing accessions) of daily RGRs in B‐deficient conditions across all imaging days. Meanwhile, 16 out of the 37 accessions which were among the 80^th^ percentile of daily RGRs in B‐deficient conditions in at least one of the first 3 imaging d were among the bottom 50^th^ percentile of accessions by the end of the imaging period. These results demonstrate that Arabidopsis seedlings become highly dependent on sufficient external B supply soon after germination, and that early vigour is not necessarily enough to overcome B limitation.

### Shoot geometrical, architectural and colour‐related phenes respond earlier to B limitation than digital shoot biomass accumulation

**Fig. 3:**
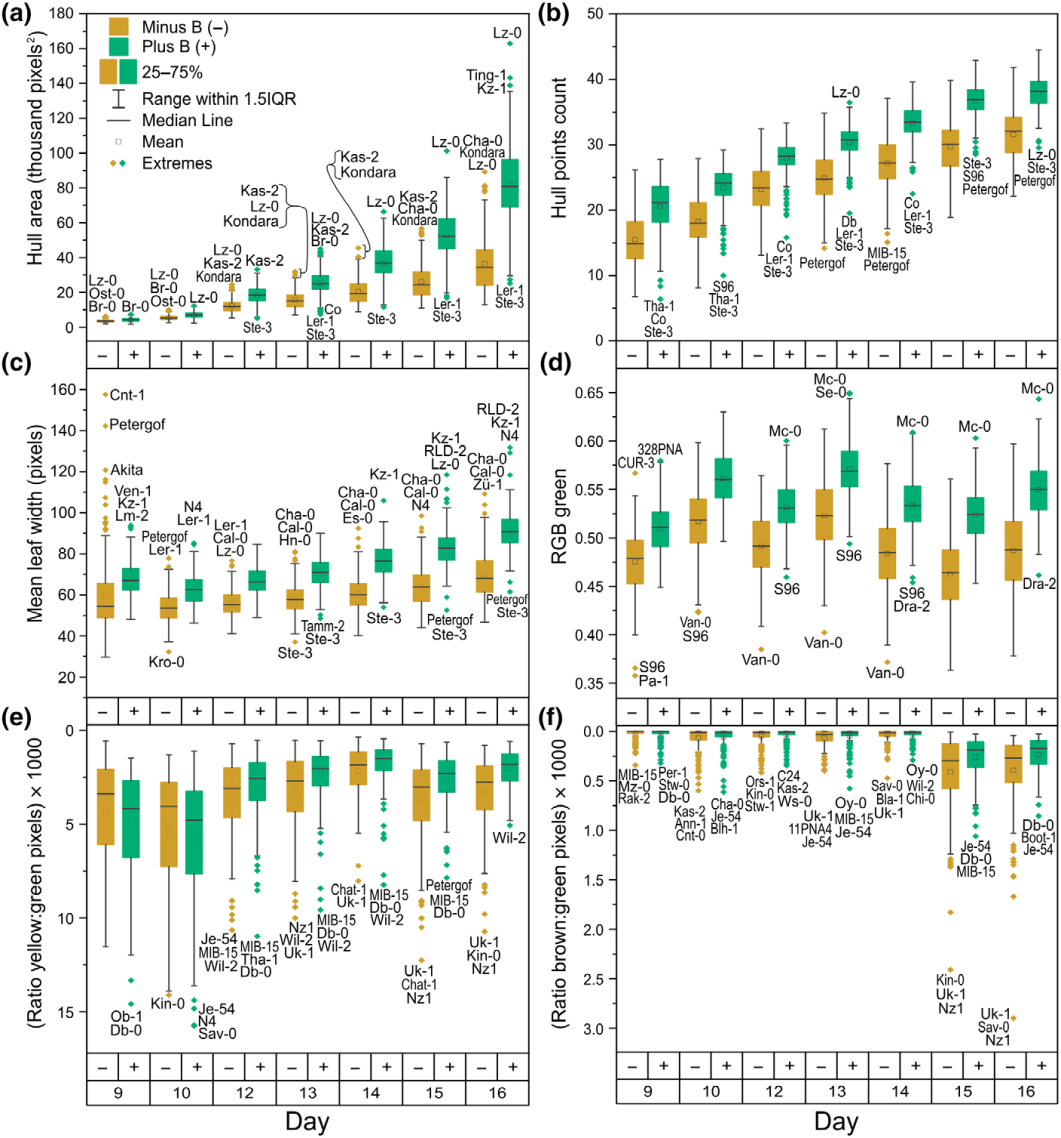
A summary of diverse Arabidopsis accession shoot performance under boron (B)‐deficient compared to B‐sufficient conditions. Shown are the area of the plant convex hull (a), hull points count (b), mean leaf width (c), average intensity of the green channel of the red green blue colour model (RGB green; d), and ratios of yellow (e) and brown (f) to green pixels. Each trait is shown daily across the experimental duration (measurements between 9 and 16 d after sowing) of plants grown in either boron (B)‐deficient (−; < 0.1 mg B kg^−1^ soil) or B‐sufficient (+; 2.5 mg B kg^−1^ soil) conditions in soil in an automated phenotyping experiment. Boxes represent the 25^th^ to 75^th^ percentiles, and whiskers represent the range within 1.5 times the interquartile range (IQR) with any extremes shown. The identities of up to three extreme accessions above or below the non‐extreme data range is indicated next to the corresponding data points where such extremes exist. Note the *Y*‐axis scales for the ratios of yellow : green and brown : green pixels are mirrored since, for these traits, a higher trait score corresponds to a more B‐deficiency sensitive accession.

In order to identify shoot behaviours associated with resilient biomass accumulation under B limitation, particularly those which could be distinguished from early in plant development, we examined diverse traits measured in the automated phenotyping system. The area of the plant convex hull, defined as the area of the smallest convex polygon drawn around each rosette (Table S3), generally followed PLA in terms of the temporal response to B deficiency, namely that distinct differences between B treatments across all accessions first became clear *c*. 12 DAS (Fig. 3a). However, the number of hull points, that is, the number of edge points of the convex hull, already responded to B deficiency from nine DAS (Fig. 3b). All other things equal, a rosette with fewer hull points requires fewer points to draw a convex hull around individual leaves. This implies a greater incidence of lanceolated leaves, which are a typical symptom of B deficiency in Arabidopsis. At nine DAS, plants exposed to B limitation had on average 24.6% fewer hull points than those grown in B‐sufficient conditions. Since PLA (Fig. 2a) and leaf count (Table S1) only marginally differed between B treatments at least until 12 DAS, the observed differences in hull points count between treatments from nine DAS suggest that leaf and rosette shape responded to B‐deficiency before biomass accumulation was impacted. Indeed, leaf width was also already negatively impacted by B limitation at nine DAS, at which point it was 11.4% smaller than in plants grown with sufficient B across accessions (Fig. 3c). Together, these results suggest a B‐limitation induced restriction in leaf cell division and expansion already from early in development, manifesting as the typical lanceolated leaves of B‐deficient Arabidopsis plants.

Colour‐related phenes made up a bigger proportion of the traits differing by more than 20% between B treatments from 9 to 12 DAS (23%) than from 13 to 16 DAS (15%). RGB green values were generally lower across all imaging days in B‐deficient growth conditions, ranging from a 7% decrease (9 DAS) to an 11% decrease at 16 DAS (Fig. 3d). Both the ratios of yellow to green (Fig. 3e) and brown to green (Fig. 3f) pixels also responded to B‐deficiency. However, differences in these traits between B treatments typically first appeared from 15 to 16 DAS; therefore, representing colour changes that occurred later in the experimental duration. In order to assess whether this had an impact on photosynthesis, the photosynthetic operating efficiency was measured at 16 DAS using a FluorCam device. In contrast to our expectations, photosynthetic operating efficiency was on average 4.7% higher in plants subjected to B‐deficient compared to B‐sufficient conditions across accessions (Fig. S3). However, no significant correlations were observed between photosynthetic operating efficiency under B deficiency and shoot FW, shoot DW, or PLA at Day 16. This result suggests that B deficiency only had a marginal effect on photosynthetic operating efficiency within the experimental duration.

### Highly B‐efficient accessions exist within the Arabidopsis population

**Fig. 4:**
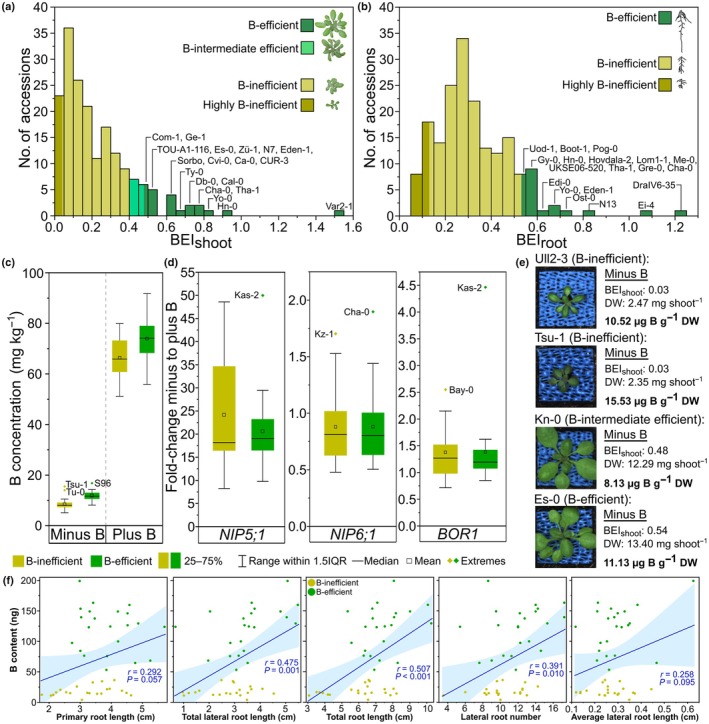
Variation in shoot boron (B) concentration and expression of B transporters in B‐efficient and B‐inefficient Arabidopsis accessions. Histograms in (a) and (b) show the frequency distribution of shoot and root B‐efficiency index (BEI_shoot_ and BEI_root_), respectively, across the population of 185 Arabidopsis accessions. Colour coding indicates the thresholds for grouping accessions into the different B‐efficiency categories. The identities of accessions within the B‐efficient categories for both BEI_shoot_ and BEI_root_ are indicated above the respective bars. (c) Shoot B concentrations of three pools of three representative biological replicates of 43 selected accessions. ‘B‐efficient’ boxes comprise data from 17 and 5 accessions defined as B‐efficient and B‐intermediate efficient, respectively, and ‘B‐inefficient’ boxes comprise data from 3 and 18 accessions defined as B‐inefficient or highly B‐inefficient, respectively. Data is shown of plants grown in soil in both B‐deficient (Minus B) and B‐sufficient (Plus B) growth conditions in an automated plant phenotyping system for 20 d. (d) Fold change in expression of *NIP5;1*, *NIP6;1*, and *BOR1* in B‐deficient compared to B‐sufficient conditions of 15‐d‐old roots of the same 43 accessions grown in an *in vitro* Phytagel‐based modified half‐strength MS media root phenotyping experiment. For all box plots, boxes represent the 25^th^ to 75^th^ percentiles and whiskers represent the range within 1.5 times the interquartile range (IQR) with any extremes shown. The identities of extreme accessions are listed next to the corresponding data points. (e) Images of accessions with similar shoot B concentrations when grown in B‐deficient conditions but contrasting BEI_shoot_ scores (Ull2‐3, Es‐0) and an accession with a low BEI_shoot_ despite a relatively high shoot B concentration when grown in B‐deficient conditions (Tsu‐1) and vice versa (Kn‐0). DW refers to shoot dry weight. (f) Correlations between root architectural traits and B content under B limitation for B‐inefficient (yellow) and B‐efficient (green) accessions. Blue lines represent linear fit regression curves with 95% confidence interval bands shown. Pearson correlation coefficients (*r*) shown in each panel.

In order to identify the most B‐efficient accessions in the population, all accessions were assigned a shoot B‐efficiency index (BEI_shoot_; Fig. 4a; Table S1), which simultaneously considers ratios between FW at 20 DAS and PLA at 16 DAS (eqn 1 in Methods S1). The average BEI_shoot_ was 0.230 and ranged from 0.016 (Kz‐1) to 1.521 (Var2‐1). The Arabidopsis reference accession Col‐0 ranked 156^th^ out of the 185 accessions in the study, with a BEI_shoot_ of 0.057. The BEI_shoot_ scores across accessions exhibited a right‐skewed distribution, with normality rejected by a Shapiro–Wilk test at *P* < 0.001 (Fig. 4a), indicating an enrichment of B‐inefficient accessions within the population suggesting that B‐efficiency is rare in Arabidopsis. Accordingly, 114 accessions (62% of the population) had lower BEI_shoot_ scores than the population‐wide mean average. The 19 accessions of the 90^th^ BEI_shoot_ percentile were considered B efficient (Var2‐1, Hn‐0, Yo‐0, Cha‐0, Tha‐1, Db‐0, Cal‐0, Ty‐0, Sorbo, Cvi‐0, Ca‐0, CUR‐3, TOU‐A1‐116, Es‐0, Zü‐1, N7, Eden‐1, Com‐1 and Ge‐1). These all had BEI_shoot_ values higher than 0.478. Of these accessions, Var2‐1, Hn‐0, Yo‐0, Tha‐1, Ca‐0, Db‐0 and Cha‐0 (7 out of 185 accessions) maintained > 80% shoot FW and DW under B‐deficient compared to B‐sufficient growth conditions, and are therefore suggested to be highly B‐deficiency tolerant accessions. Shoot DWs of Hn‐0, Yo‐0 and Var2‐1 were even 2%, 3% and 33% higher under B‐deficiency than B‐sufficiency, respectively. However, all of these accessions apart from Ca‐0 had below population‐average shoot DW under B‐sufficient conditions. This was in fact true for 13 out of the 19 most B‐efficient (BEI_shoot_ 90^th^ percentile) accessions, indicating that a vigourous biomass formation under B‐deficient conditions does not automatically associate with a vigourous biomass formation under B‐sufficient conditions. Meanwhile, the shoot FW and DW of two B‐efficient accessions, Cal‐0 and Es‐0, fell within the 80^th^ percentile (37 top performing accessions) in B‐sufficient conditions, which suggests that these accessions are not only B‐efficient but also generally vigourous. Eleven accessions with BEI_shoot_ values above 0.400 were considered B‐intermediate efficient (Kn‐0, Bl‐1, Kas‐2, Bs‐1, S96, Liarum, In‐0, Kondara, Je‐54, Zdr‐1 and Co). All of the B‐efficient or B‐intermediate efficient accessions maintained > 63% PLA at day 16 as well as > 62% shoot DW and > 56% shoot FW under B‐deficient compared to B‐sufficient growth conditions (Fig. S4). Additionally, under B‐deficient conditions, all B‐efficient accessions had a shoot FW at experiment end above the population‐wide mean, and 17 of the 19 B‐efficient accessions had a shoot DW and PLA at Day 16 above the population‐wide mean (Fig. S4). The two accessions with below population‐wide mean shoot DW and PLA at day 16 under B‐deficient conditions (Tha‐1 and Db‐0) were still considered to be B efficient due to their strong ability to maintain over 80% of their respective shoot FW, shoot DW and PLA at day 16 under B‐sufficient conditions. Amongst the remaining accessions, we defined the 136 accessions with BEI_shoot_ over the 10^th^ percentile as B‐inefficient lines and the 19 accessions within the 10^th^ percentile of BEI_shoot_ (< 0.043) as highly B‐deficiency sensitive (Ga‐0, Hovdala‐2, Pa‐1, Rmx‐A02, Hau‐0, Tu‐0, Tsu‐1, Ri‐0, Alc‐0, Kin‐0, Nz1, Ull2‐3, Van‐0, Bay‐0, WAR, Duk, RLD‐2, Gel‐1 and Kz‐1). Representative B‐efficient (Es‐0), B‐inefficient (Wag‐3, Wil and Uod‐1) and highly B‐deficiency sensitive (Kin‐0) accessions as well as a *nip5;1* knockout mutant are displayed in Fig. 1a,b, in each case on the carriers as they were grouped in the randomised complete block design (Table S4). The accessions most able to maintain biomass accumulation under B‐deficient conditions are generally those which accumulated below‐average biomass under nutrient‐replete conditions. However, the existence of highly productive accessions in both B‐sufficient and B‐deficient conditions within the population indicates that traits that confer vigour in both scenarios exist in Arabidopsis.

### Boron deficiency decreased shoot B concentrations to a similar extent in both B‐efficient and B‐inefficient accessions

The ionome of 43 accessions was determined using shoot tissue harvested 20 DAS from the automated phenotyping experiment. Boron‐efficient accessions were represented by 17 of the 19 B‐efficient accessions and five of the B‐intermediate efficient accessions. B‐inefficient accessions were represented by 17 of the 19 highly B‐deficiency sensitive accessions and 4 B‐inefficient accessions (Table S1). Boron‐efficient accessions had generally higher B concentrations than B‐inefficient accessions in both B‐sufficient (11% higher, on average) and B‐deficient (40% higher, on average) growth conditions. However, both groups were similarly affected by B deficiency, with an average 84% and 87% reduction in shoot B concentration in B‐efficient and B‐inefficient accessions, respectively, compared to B‐sufficient conditions (Fig. 4c). Under B‐sufficient growth conditions, shoot B concentration in B‐inefficient accessions varied between 51.1 mg kg^−1^ DW (Tsu‐1) and 79.9 mg kg^−1^ DW (WAR), and in B‐efficient accessions between 55.9 mg kg^−1^ DW (Var2‐1) and 91.7 mg kg^−1^ DW (Hn‐0). Shoot B concentrations in B‐deficient growth conditions varied in B‐inefficient accessions between 5.1 mg kg^−1^ DW (Hau‐0) and 15.3 mg kg^−1^ DW (Tsu‐1), and in B‐efficient accessions between 8.1 mg kg^−1^ DW (Kn‐0) and 16.8 mg kg^−1^ DW (S96; Fig. 4c). Average shoot B concentrations in the Arabidopsis reference accession, Col‐0, were 76.0 mg kg^−1^ DW in B‐sufficient conditions and 9.14 mg kg^−1^ DW in B‐deficient conditions. Despite slightly higher average shoot B concentrations in B‐efficient accessions, several individual B‐efficient and B‐inefficient accessions shared similar shoot B concentrations under B deficiency despite exhibiting contrasting B‐deficiency tolerances. For example, the B‐efficient line, Es‐0, achieved a BEI_shoot_ of 0.54 with a B‐deficient shoot B concentration of 11.13 mg kg^−1^ DW, whilst B‐inefficient Ull2‐3 had a similar shoot B concentration of 10.52 mg kg^−1^ DW but only achieved a BEI_shoot_ of 0.03, indicating increased B‐use efficiency in Es‐0 (Fig. 4e). Meanwhile, B intermediate‐efficient Kn‐0 achieved a BEI_shoot_ of 0.48 and a shoot DW of 12.29 mg with a shoot B concentration of only 8.13 mg kg^−1^ DW under B deficiency, whilst B‐inefficient Tsu‐1 only achieved a BEI_shoot_ of 0.03 and a shoot DW of 2.35 mg, despite having a B concentration of 15.53 mg kg^−1^ DW (Fig. 4e). This indicates that tolerance to B deficiency in Arabidopsis is not necessarily linked to higher shoot B concentrations.

Shoot concentrations of other mineral nutrients were generally not affected by B deficiency across the B‐efficient accessions (Figs S5, S6). However, B‐inefficient accessions exhibited lower Mg, P, Ca, Mn, Fe and Zn in B‐deficient conditions, maybe as a result of a general decline in root nutrient uptake and translocation processes. Shoot K concentrations were lower in many of the B‐inefficient accessions compared to B‐efficient accessions in both B‐sufficient (15.2% lower, *P* = 0.003) and B‐deficient (24.6% lower, *P* < 0.001) conditions (Fig. S5; Table S1), potentially indicating an osmotic adjustment component to B efficiency.

We calculated shoot B contents in order to assess how much B plant roots were able to take up and translocate to the shoot. Shoot B contents of plants exposed to B‐deficiency were on average 6.52‐fold higher in B‐efficient than B‐inefficient accessions, due to higher biomass accumulation in the former group (*P* < 0.001; Figs 4f, S7). In B‐sufficient conditions, no differences in B content between these groups were detected. Boron contents among B‐efficient accessions exposed to B deficiency varied 3.74‐fold from 53.28 (Db‐0) to 199.11 (Cal‐0) ng B per plant shoot, and in B‐inefficient accessions 9.02‐fold from 4.04 (Van‐0) to 36.46 (Tsu‐1) ng. The similar B content between B‐efficient Db‐0 and B‐inefficient Tsu‐1 indicates that some accessions are able to continue to grow even with low B accumulation, indicating a high B‐use efficiency.

### Increased lateral root length may be an adaptive response to B limitation

**Fig. 5:**
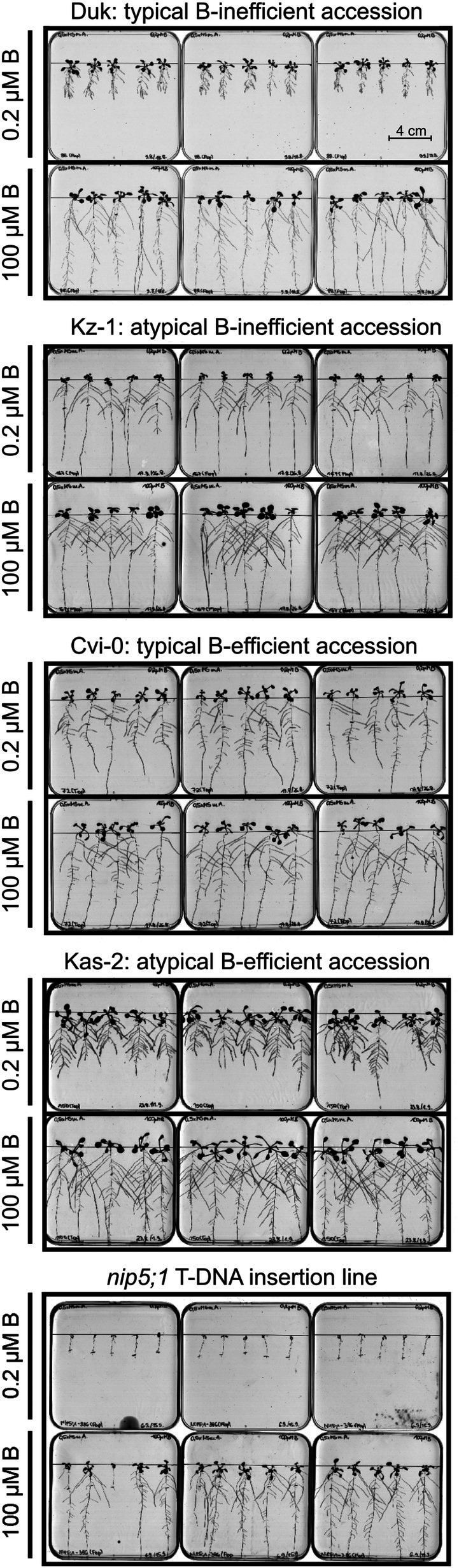
Effects of boron (B) deficiency on Arabidopsis root morphology in seedlings subjected to different B supplies. Example Petri dishes (12 × 12 cm) as used for *in vitro* root phenotyping experiments. Petri dishes contained Phytagel‐based modified half‐strength MS media either adequately (100 μM) or inadequately (0.2 μM) supplied with B. Scans of 15‐d‐old Arabidopsis accessions determined as B efficient or B inefficient in terms of shoot growth are shown; in each case, it is indicated whether root performance under B‐deficient conditions is typical (severely affected for B‐inefficient accessions, marginally affected for B‐efficient accessions) or atypical (severely affected for B‐efficient accessions, marginally affected for B‐inefficient accessions). The B transporter mutant *nip5;1* was included in Petri dish experiments as a reference.

**Fig. 6:**
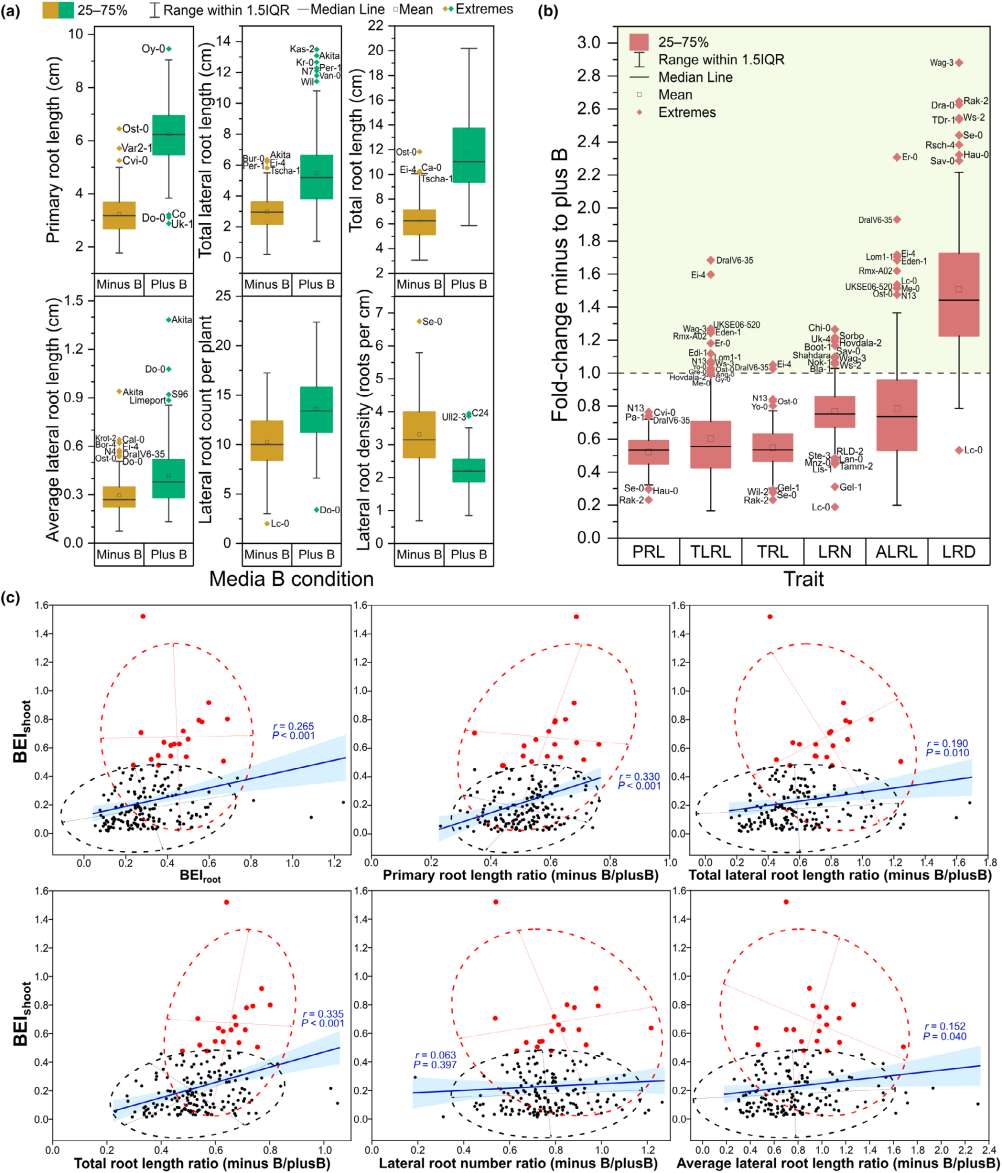
A summary of Arabidopsis accession performance for traits measured in an *in vitro* Phytagel‐based modified half‐strength MS media root phenotyping experiment. (a) Accession variation in root system architecture phenes in boron (B)‐deficient (Minus B; 0.2 μM B) and B‐sufficient (Plus B; 100 μM B) conditions. (b) Fold‐change for each phene for each accession of performance under B‐deficient compared to B‐sufficient conditions. The dashed line at *y* = 1 is shown to indicate the direction of accession response, where data above ‘1’ indicate greater phene values under B‐deficient compared to B‐sufficient conditions. For all box plots, boxes represent the 25^th^ to 75^th^ percentiles and whiskers represent the range within 1.5 times the interquartile range (IQR) with any extremes shown. The identities of extreme accessions are listed next to the corresponding data points. ALRL, average lateral root length; LRD, lateral root density; LRN, lateral root number; PRL, primary root length; TLRL, total lateral root length; TRL, total root length. (c) Correlations of root B‐efficiency Index (BEI_root_) and root architectural trait ratios against shoot B‐efficiency Index (BEI_shoot_). Blue lines represent linear fit regression curves with 95% confidence interval bands shown. Pearson correlation coefficients (*r*) shown in each panel. Dashed ellipses represent 95% confidence ellipses around accessions in the 90^th^ BEI_shoot_ percentile (in red) and all other accessions (in black).

To assess whether root traits can respond to B deficiency as an adaptive response to B limitation and therefore contribute to B accumulation, we determined root system architecture traits of the 185 accessions under B‐sufficient and B‐deficient conditions in a Petri‐dish cultivation system (Fig. 5). The B transporter mutant *nip5;1* was included as a quality control check that B deficiency was indeed induced. As expected, no plants of *nip5;1* significantly continued to grow after transplantation to the B‐deficient growth condition. A direct comparison between *nip5;1* and the accession Col‐0 from which it is derived is summarised in Fig. S1(b). Variation in primary root length (PRL) was similar for B‐sufficient and B‐deficient growth conditions, ranging 3.28‐fold from 2.88 cm (Uk‐1) to 9.46 cm (Oy‐0) and 3.65‐fold from 1.77 cm (Hau‐0) to 6.45 cm (Ost‐0), respectively (Fig. 6a). The Arabidopsis reference accession, Col‐0, exhibited PRLs of 7.27 and 3.86 cm in B‐sufficient and B‐deficient conditions, respectively. Total root length (TRL) varied 3.44‐fold between Wag‐3 (5.87 cm) and Kas‐2 (20.19 cm) in B‐sufficient conditions, and 3.86‐fold between Gel‐1 (3.07 cm) and Ost‐0 (11.83 cm) in B‐deficient conditions (Fig. 6a; Table S1). Variation in total lateral root length (TLRL) was higher in B‐deficient conditions, ranging 29.10‐fold from 0.22 cm (Tamm‐2) to 6.35 cm (Akita) under B‐deficiency and 12.53‐fold from 1.08 cm (Wag‐3) to 13.49 cm (Kas‐2) under B‐sufficiency. Primary root length decreased in all accessions in B‐deficient compared to B‐sufficient conditions, ranging from a 23.6% PRL reduction in N13 to a 76.9% reduction in Rak‐2 (Fig. 6b). Meanwhile, TLRL of 15 accessions was higher in B‐deficient than B‐sufficient conditions, ranging from a 1.32% (Ang‐0) to 68.42% (DraIV6‐35) increase under B deficiency and indicating a possible adaptive response. An increase in average lateral root number (LRN) per plant was observed in 15 accessions under B deficiency, although only two of these were among the accessions with an increase in TLRL (Table S1). Thirty‐eight accessions also exhibited an increase in average lateral root length (ALRL) under B‐deficiency, comprising all 15 accessions that exhibited an increased TLRL (Fig. 6b). Finally, lateral root density (LRD) showed generally higher values in B‐deficient compared to B‐sufficient conditions, due to the generally larger decrease in PRL than LRN under B deficiency.

To assess whether the B‐responsive root parameters affected shoot B uptake and accumulation under B limitation, we performed correlation analyses between the respective traits (Figs 4f, S8). Correlations between root traits and shoot B content were generally more significant than between root traits and shoot B concentration. The strongest relationship observed was between TRL and shoot B content under B limitation (Pearson *r* = 0.507, *P* < 0.001; Fig. 4f). Interestingly, PRL most strongly correlated with shoot B concentration (Pearson *r* = 0.328, *P* = 0.032), whilst TLRL more strongly correlated with shoot B content (Pearson *r* = 0.475, *P* = 0.001). Under B limitation, B‐efficient accessions did not necessarily have higher B concentrations than B‐inefficient accessions (Fig. 4e), whereas all B‐efficient accessions had higher B contents than B‐inefficient accessions (Fig. S7). Therefore, whilst primary roots appear to contribute to B uptake, lateral roots appear to have a bigger impact on maintaining a vigourous shoot growth. This is likely due to them opening up a greater soil volume from which to access a constant supply of B to the developing shoot even when such supply is very low. We also assessed the relationship between root traits and shoot performance under B‐deficiency. The ratio of PRL under B‐deficient compared to B‐sufficient conditions was more closely correlated to BEI_shoot_ than lateral root related traits, with a Pearson *r* of 0.330 (*P* < 0.001), compared to 0.190 for TLRL (*P* = 0.010), 0.063 for lateral root number (LRN; not‐significant at *P* < 0.05) and 0.152 for ALRL (*P* = 0.040; Fig. 6c). However, B‐efficient accessions could be more strongly distinguished by their ability to maintain lateral root traits: the ratio of TLRL under B‐deficient compared to B‐sufficient conditions was 30.9% bigger on average in B‐efficient accessions than all other accessions, and 22.4% bigger for ALRL (Fig. 6c). Meanwhile, the LRN ratio was only 5.92% higher in B‐efficient compared to B‐inefficient accessions, indicating that extending lateral roots once formed is more important for B efficiency than investing in new lateral roots.

Accessions were assigned a root B‐efficiency index (BEI_root_) which considers the ratios of obtained PRL and TLRL values in B‐deficient compared to B‐sufficient growth conditions. The average BEI_root_ was 0.323 and ranged from 0.054 (Rak‐2) to 1.243 (DraIV6–35; Fig. 4b). The BEI_root_ for the Arabidopsis reference accession, Col‐0, was 0.228. Similar to BEI_shoot_ scores, BEI_root_ scores exhibited a right‐skewed distribution, with normality rejected by a Shapiro–Wilk test at *P* < 0.001 indicating an enrichment of accessions within the population whose primary and lateral root growth is highly sensitive to B limitation. We classified accessions of the 90^th^ BEI_root_ percentile (> 0.52) as B‐efficient (19 accessions; Table S1). Five accessions, Cha‐0, Eden‐1, Tha‐1, Hn‐0 and Yo‐0, were in the 90^th^ percentile of both BEI_shoot_ and BEI_root_ and so were considered highly B‐deficiency tolerant. Four accessions, Van‐0, Kz‐1, RLD‐2 and Gel‐1, were in the 10^th^ percentile of both BEI_shoot_ and BEI_root_ and so were considered highly B‐deficiency sensitive. Two accessions, Uod‐1 and Hovdala‐2, were in the 90^th^ percentile of BEI_root_ but in the 10^th^ percentile of BEI_shoot_. By contrast, none of the accessions with the most resilient shoot biomass accumulation under B deficiency (BEI_shoot_ 90^th^ percentile) had highly B‐deficiency sensitive roots (BEI_root_ 10^th^ percentile), and all but two of these accessions (Cal‐0 and Com‐1) had TRL within the top 50^th^ percentile. This indicates that accessions with high shoot performance under B‐deficiency also had root systems that were not severely inhibited. Whilst the Petri‐dish cultivation system was optimised for measuring root rather than shoot responses, we also measured shoot DW at experiment end for all accessions grown in B‐sufficient and B‐deficient conditions in the Petri‐dish system. Under B deficiency, shoot DW ranged 7.50‐fold from 0.078 to 0.585 mg per plant, and under B sufficiency it ranged 3.82‐fold from 0.186 to 0.710 mg per plant. A significant correlation was observed between PLA at day 10 as measured in the automated phenotyping experiment and shoot DW as measured in the Petri‐dish experiment (*r* = 0.284; Fig. S9). PLA at day 10 was selected as the most suitable biomass‐related trait from the automated phenotyping experiment to compare with shoot DW from the agar plate experiment, as shoot DW from the agar plate experiment was measured after plants had been exposed to the different B conditions for 10 d. Whilst the specific ranking of shoot biomass‐related traits in most accessions was not conserved between the growth systems, several extreme B‐efficient or B‐deficiency sensitive accessions clearly had above or below average performance, respectively, under B deficiency in both growth systems, confirming variation in B efficiency is stable in this population.

### Accessions with contrasting tolerance to B deficiency show neither differences in the expression of B transporters nor their amino acid sequence

Transcript abundance of three physiologically relevant B transporters *NIP5;1*, *NIP6;1* and *BOR1* (Matthes *et al*., 2020) was determined in roots of seedlings of the same 43 accessions selected for shoot ionome analysis (Figs 4d, S10; Table S5). *NIP5;1* was upregulated by B deficiency in all accessions, ranging from 8.25‐fold to 49.98‐fold upregulation. *NIP5;1* was upregulated 22.70‐fold under B deficiency in the reference accession, Col‐0. Average upregulation did not significantly differ between the B‐efficient (20.62‐fold) and B‐inefficient (24.17‐fold) accessions (*P* = 0.24). Both groups contained accessions with higher (Kas‐2, B efficient, 49.98‐fold; Duk, B inefficient, 48.59‐fold) and lower (Ca‐0, B efficient, 9.76‐fold; Rld‐2, B inefficient, 8.25‐fold) upregulation. *NIP6;1* was not particularly differentially expressed in response to B deficiency, with no accessions exhibiting greater than 2‐fold up‐ or downregulation. *BOR1* was on average 1.38‐fold upregulated under B‐deficient conditions but varied 5.25‐fold among B‐efficient accessions from 0.85 to 4.46 and 3.56‐fold among B‐inefficient accessions from 0.72 to 2.55 (Fig. 4d). There was no difference in the *BOR1* expression response between B‐efficient and B‐inefficient accessions (*P* = 0.98). No significant correlations were detected between expression levels of any of the three transporters and BEI_shoot_ (Fig. S11). In addition, root expression levels of these transporters did not correlate with shoot B concentration or content in B‐deficient growth conditions (Fig. S11), indicating that additional factors contribute to differences in B concentration between accessions. Kas‐2, a B‐intermediate efficient accession, had the highest upregulation amongst all accessions of both *NIP5;1* and *BOR1* in B‐deficient conditions (49.98‐fold and 4.46‐fold, respectively), indicating a possible B‐transporter driven adaptive response in this accession.

Additionally, we performed a haplotype analysis in the coding sequences of NIP5;1, NIP6;1 and BOR1 to test whether accession‐specific amino acid sequence variations, potentially altering B transport capabilities, associate with shoot B contents or BEI_shoot_ values. Across 104 accessions for which haplotype data were available in the SNP★ar tool used, two, two and three non‐synonymous amino acid substitutions were identified in NIP5;1, NIP6;1 and BOR1, respectively (Fig. S12a). None of the accessions with amino acid substitutions in NIP5;1 were classified as either B efficient or B inefficient in this study. Both substitutions in NIP6;1 existed in both efficient and inefficient accessions. Meanwhile, two out of the three substitutions in BOR1 existed in accessions with low (10^th^ percentile) but not high (90^th^ percentile) BEI_root_ scores, but at frequencies too low to confirm a role in B efficiency. In addition, none of BEI_shoot_ (Fig. S12b), BEI_root_ (Fig. S12c), shoot B concentration (Fig. S12d) or shoot B content (Fig. S12e) were significantly different between haplotype groups, indicating that the substitutions existing within the accessions studied did not contribute to B efficiency.

### Boron‐efficient accessions cluster phylogenetically but geographically only to very localised regions

**Fig. 7:**
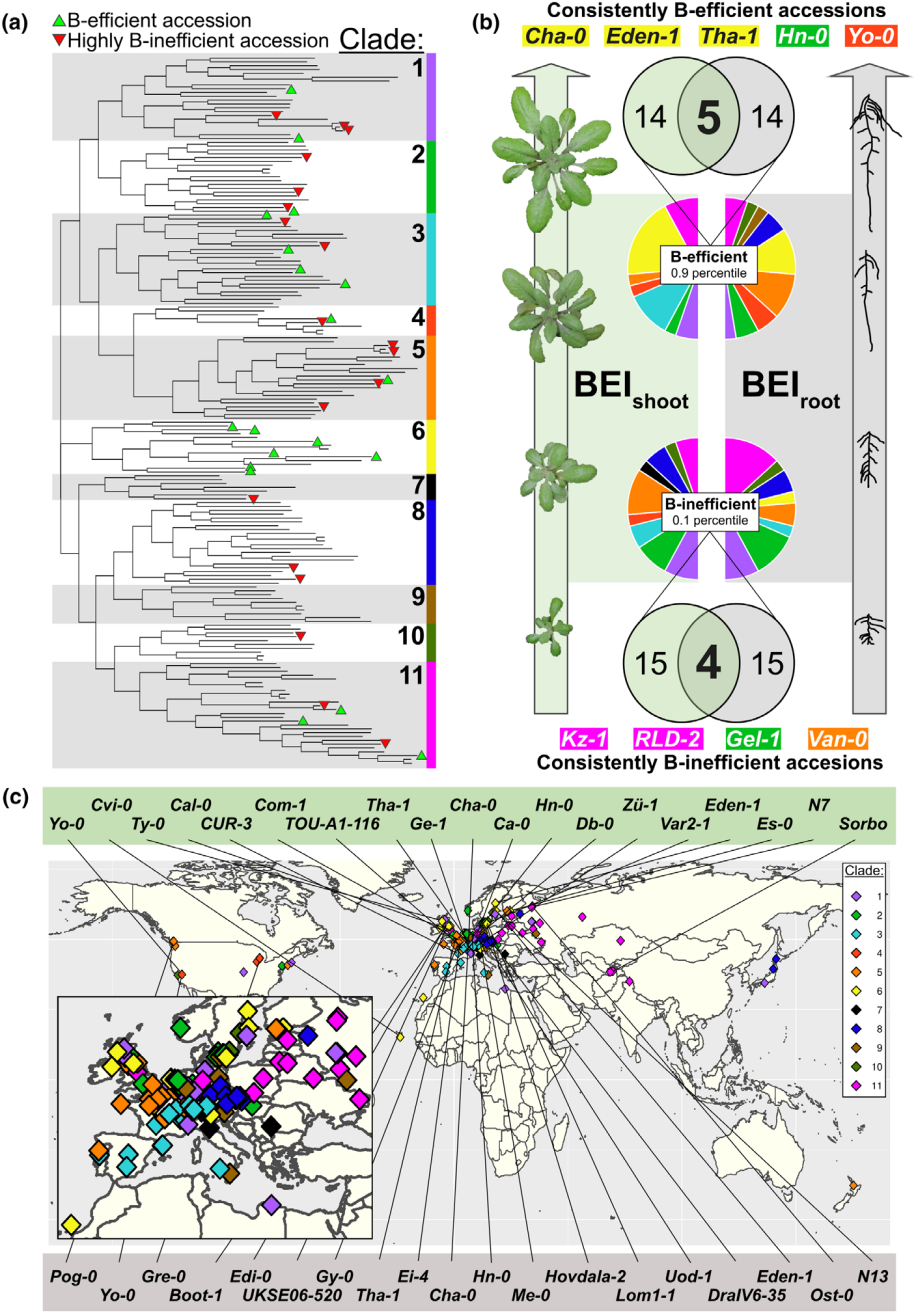
Phylogeny and geography of boron (B)‐efficiency in Arabidopsis. (a) Dendrogram showing phylogenetic relationships of the 185 Arabidopsis accessions, with 11 manually assigned clades indicated. Green triangles represent B‐efficient accessions of the shoot B‐efficiency Index (BEI_shoot_) 90^th^ percentile (*n* = 19). Red triangles represent B‐inefficient accessions of the BEI_shoot_ 10^th^ percentile (*n* = 19). (b) Proportion of accessions of the BEI_shoot_ and BEI_root_ 10^th^ and 90^th^ percentiles that exist within each phylogenetic clade. Colour‐coding in pie charts represents phylogenetic clades as indicated in (a). Sizes of slices of pie reflect the proportion of the 10^th^ (lower pie) or 90^th^ (upper pie) percentile accessions that were grouped into each clade. Accessions listed at the top or bottom of the panel are those that were ranked consistently B‐efficient or B‐inefficient, respectively, in both shoot and root datasets, colour‐coded by phylogenetic clade as indicated in (a). (c) Location of sampling point of all 185 accessions colour‐coded by phylogenetic clade as indicated in (a). Inset in (c) is a zoomed image centered around European accessions. Top performing accessions (90^th^ percentiles) of the BEI_shoot_ (top, green background) or BEI_root_ (bottom, grey background) are indicated.

We employed a phylogenetic clustering approach based on 214 051 SNPs to identify shared genetic bases and evolutionary origins of B efficiency in Arabidopsis (Fig. S13). Eleven distinct clades were detected (Fig. 7a). Seven of the 14 accessions within clade 6 were defined as B efficient; therefore, representing a 4.87‐fold enrichment of B‐efficient accessions above what would be expected by chance within this clade. This included three out of the five accessions defined as B efficient based on both BEI_shoot_ and BEI_root_ scores (Fig. 7b). Five of the remaining seven accessions in clade 6 were also within the 70^th^ BEI_shoot_ percentile and were therefore also relatively B efficient. Interestingly, the two B‐deficiency sensitive Finnish accessions Tamm‐2 and Tamm‐27 (BEI_shoot_ of 0.11 and 0.08, respectively) also fell within this clade. The B‐efficient accession Es‐0 (BEI_shoot_ of 0.54) of clade 6 was sampled only *c*. 70 km away from Tamm‐2 and Tamm‐27 (Table S1) suggesting a shared common ancestor but a more recent divergence in their B efficiency. Three other accessions from clade 6, Eden‐1, Var2‐1 and Ost‐0, originated in Sweden; therefore, relatively nearby to the previously mentioned accessions of this clade, further suggesting a shared recent ancestor for these accessions. Areas of Scandinavia were characterised as having B‐deficient soils (Shorrocks, 1997), which may have provided a selection pressure for adapting to low B environments. Clade 3 contains four accessions characterised as B efficient (CUR‐3, TOU‐A1‐116, Zü‐1 and Ge‐1), as well as two accessions defined as B‐intermediate efficient accessions (Bs‐1 and Co). Two of the five accessions that had consistently low BEI scores in both shoots and roots, Kz‐1 (from Kazakhstan) and RLD‐2 (from Iran), clustered in clade 11 (Fig. 7b). The poor performance of these genotypes in B‐deficient conditions may indicate a low selection pressure for B‐efficiency at their places of origin.

The geographic origin of accessions is shown in Fig. 7(c). A clustering of accessions from clade 6 can be seen around Nordic countries. In general, phylogenetic clades clustered into rough geographic regions. All the 11 distinct phylogenetic groups comprise differently B‐efficient accessions partly originating only from different regions of the same country. This suggests that localised conditions with low B‐availability led to multiple localised and independent B‐efficiency adaptations in Arabidopsis.

### Phenotypic response profiling revealed 12 adaptive root and shoot performance patterns under B deficiency

**Fig. 8:**
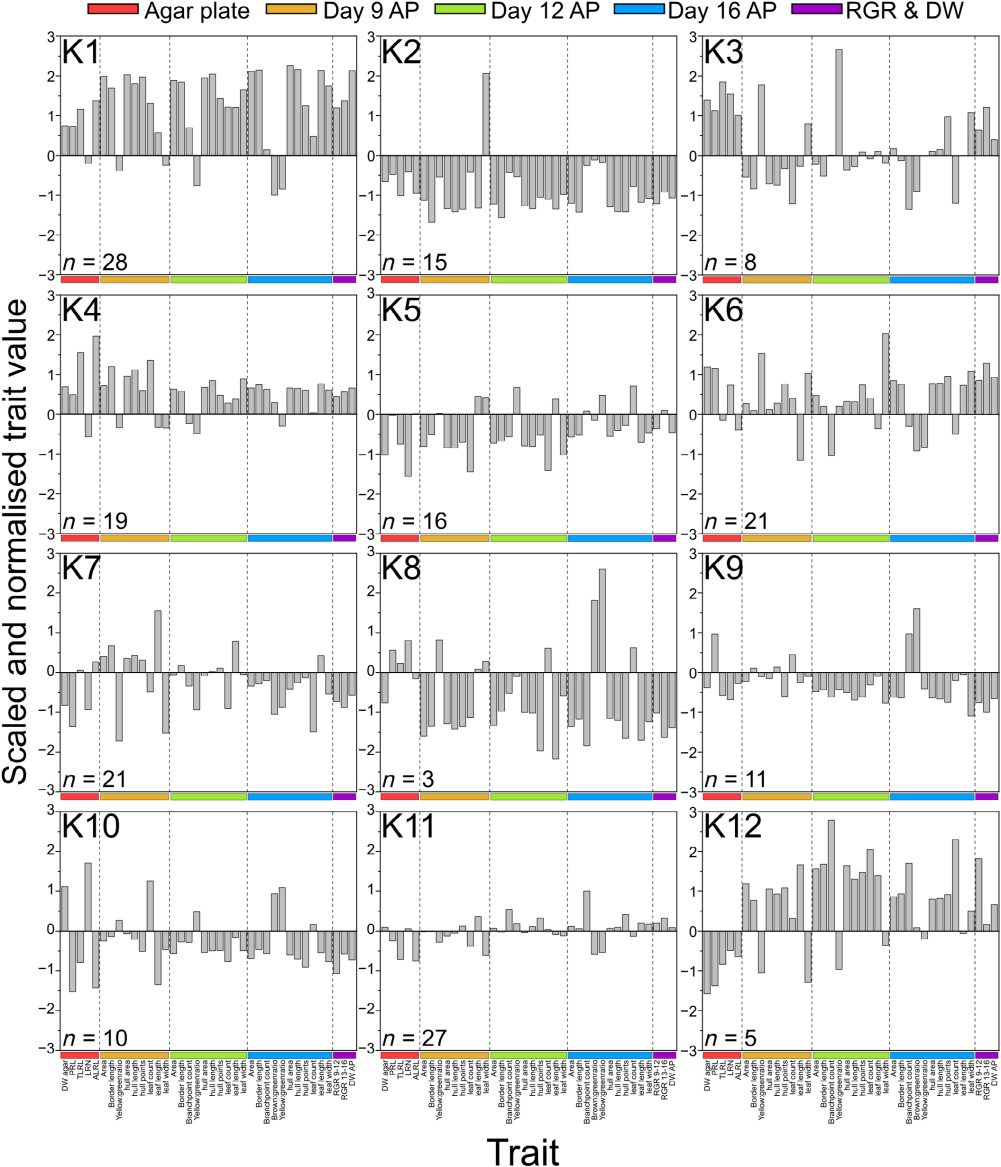
Centered and scaled Arabidopsis root and shoot trait ratios between boron (B)‐deficient and B‐sufficient growth conditions of the 38 traits included in *k*‐means clustering. The average trait ratios of the 12 *k‐*clusters (K1–K12) are shown separately, with the number of Arabidopsis accessions included within each *k*‐cluster (*n*) indicated. Centering was performed by subtracting the trait ratio mean from each accession ratio. Scaling was performed by dividing this value by the trait ratio SD. ALRL, average lateral root length; AP, automatic phenotyping experiment; DW, dry weight; LRN, lateral root number; PRL, primary root length; RGR, relative growth rate; TLRL, total lateral root length.

To identify shared physiological responses to B deficiency across the range of measured traits, we grouped accessions using *k*‐means clustering whereby each accession was grouped into the *k*‐cluster with the nearest mean across the range of input trait data. The aim was to identify root and shoot physiological patterns that associate with high biomass formation resilience to B limitation via an unsupervised machine learning approach. We selected 38 traits to represent root and shoot responses to B limitation (Fig. S14). Centred and scaled means of the 38 trait ratios for the accessions included in each cluster are presented in Fig. 8. Cluster 1 contains 28 accessions that together had the highest average DW ratio in the automated phenotyping experiment compared to other clusters (Fig. 8). Eleven of the 19 accessions in the BEI_shoot_ 90^th^ percentile grouped into cluster 1. Accessions within this cluster had relatively high performance across traits. The *k*‐mean of LRN within cluster 1 was close to zero, and therefore, this trait was not associated with the high B efficiency among accessions of this cluster. However, all other root traits had relatively high *k*‐means, most notably ALRL and TLRL. Both ALRL and TLRL were strongly correlated across the accessions, and both generally correlated with shoot biomass accumulation (Fig. S15). Clusters 3 (8 accessions) and 4 (19 accessions) also exhibited a high *k*‐mean TLRL and a higher‐than‐average shoot DW ratio. However, different B‐efficiency mechanisms appear to be present in other *k*‐clusters. Cluster 6 (21 accessions) is characterised by a relatively good shoot performance and only average TLRL and ALRL under B‐deficient conditions but a high average PRL and LRN. Cluster 12 (5 accessions) also maintained a relatively good shoot performance under B‐deficiency, but with generally poor root performance. Meanwhile, cluster 2 (15 accessions) had low *k*‐means across most trait ratios. All accessions grouped within this cluster were also in the bottom 50^th^ percentile for BEI_shoot_ (all < 0.14). Taken together, this analysis suggests that maintaining a large root system under B‐deficient conditions and in particular producing long lateral roots, appears to be an efficient strategy to maintain shoot productivity, as is also evident from correlations between root traits and shoot DW of plants grown in B‐deficient conditions (Fig. S16).

### Genome‐wide association study identified a QTL on chromosome 4 that controls resilience of multiple traits to B deficiency

**Fig. 9:**
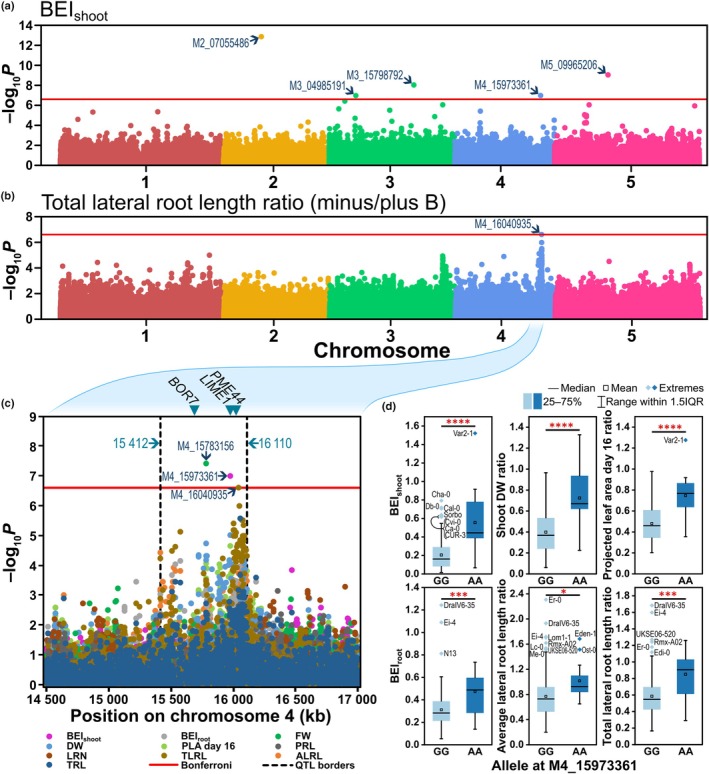
Analysis of genome‐wide single nucleotide polymorphism (SNP) associations with shoot and root trait performance under boron (B)‐deficient compared to B‐sufficient conditions within 185 Arabidopsis accessions. (a) Genome‐wide SNP associations with B‐efficiency index of the shoot (BEI_shoot_). (b) Genome‐wide SNP associations with the ratio of total lateral root length (TLRL) under B‐deficient compared to B‐sufficient conditions. (c) Genome‐wide SNP associations with BEI_shoot_, B‐efficiency index of the root (BEI_root_), and ratios of trait performance under B‐deficient compared to B‐sufficient conditions of shoot fresh (FW) and dry (DW) weight, projected leaf area (PLA) at day 16, primary root length (PRL), lateral root number (LRN), total lateral root length (TLRL), average lateral root length (ALRL) and total root length (TRL) within a loci of multiple trait associations on chromosome 4. Horizontal red lines in (a–c) represent the Bonferroni corrected significance threshold at *P* < 0.05. Marker names are listed where marker‐trait associations exceeded this threshold, with arrows pointing to the relevant marker. A QTL region between 15 412 and 16 110 kb on chromosome 4 was defined, within which marker‐trait associations were observed for multiple root and shoot traits (c). The QTL borders were defined by the positions of the outermost markers with −log_10_ *P* values of at least 4, +/− 3.365 kb (half linkage disequilibrium decay value of the original population; Meyer *et al*., 2021). The positions of three candidate genes from within this region, *BOR7*, *LIME1* and *PME44*, are shown above as indicated by triangles. (d) Effects of alternative alleles at marker position M4_15973361 (the lead marker for BEI_shoot_ within the defined QTL on chromosome 4) on BEI_shoot_, BEI_root_ and a selection of shoot and root performance under B‐deficient compared to B‐sufficient conditions ratio traits. Stars above boxes represent trait differences depending on allele at *, *P* < 0.05; ***, *P* < 0.001; ****, *P* < 0.0001 as tested using individual one‐way ANOVAs. Boxes represent the 25^th^ to 75^th^ percentiles and whiskers represent the range within 1.5 times the interquartile range (IQR) with any extremes shown.

To identify genetic loci controlling shoot and root resilience to B‐deficient conditions, we employed a genome‐wide association (GWA) approach based on 201 572 SNPs (Meyer *et al*., 2021). Significant marker‐trait associations were identified for BEI_shoot_ (5), and for ratios of shoot FW (7) and TLRL (1) under B‐deficient compared to B‐sufficient conditions (Figs 9a,b, S17, S18; Tables 1, S6), in each case with ‐log_10_ *p* values above those which were expected by chance (Figs S19, S20). Whilst significant trait associations among the 13 GWA analyses shown in Figs S17 and S18 were limited to these three traits, we noticed additional distinct association peaks around a locus of chromosome 4 for shoot DW, PLA at day 16, BEI_root_, ALRL and TRL that co‐localised with significant marker‐trait associations for BEI_shoot_, shoot FW and TLRL (Fig. 9c). We defined a B‐efficiency QTL between position 15 412 038 and 16 110 376 on chromosome 4, defined by the positions of the outermost markers with −log_10_ *P* values of at least 4, ±3.365 kb (half linkage disequilibrium decay value of the original population; Meyer *et al*., 2021; Fig. 9c). Within this locus lie the B transporter encoding gene *BOR7* (AT4G32510), and the pectin methylesterase encoding gene *PME44* (AT4G33220). The ‘S‐adenosyl‐l‐methionine‐dependent methyltransferases superfamily protein’ encoding gene *LIME1* (AT4G33110) is also suggested as a strong candidate within this locus, as the leading SNP associated with BEI_shoot_ at this locus (M4_15973361) falls within a coding region of this gene.

**Table 1:** Significant marker‐trait associations identified through genome‐wide association analyses.

Marker name	Associated with	Prevalence of B‐efficient allele (%)	Average BEI_shoot_ with:	
			B‐efficient allele	B‐inefficient allele
M2_00667596	Shoot FW	44	0.276 ± 0.247	0.192 ± 0.163
M2_07055486	BEI_shoot_, shoot FW	7	0.499 ± 0.386	0.209 ± 0.174
M3_00880333	Shoot FW	36	0.331 ± 0.266	0.174 ± 0.138
M3_04985191	BEI_shoot_	6	0.565 ± 0.386	0.209 ± 0.172
M3_15798792	BEI_shoot_, shoot FW	81	0.251 ± 0.212	0.138 ± 0.161
M3_16664417	Shoot FW	53	0.279 ± 0.226	0.179 ± 0.178
M4_15783156	Shoot FW	8	0.524 ± 0.251	0.206 ± 0.184
M4_15973361	BEI_shoot_	7	0.555 ± 0.391	0.205 ± 0.164
M4_16040935	Total lateral root length	5	0.459 ± 0.273	0.217 ± 0.196
M5_06440519	Shoot FW	29	0.325 ± 0.277	0.189 ± 0.160
M5_09965206	BEI_shoot_	17	0.404 ± 0.333	0.195 ± 0.150

For each marker, the prevalence of the allele with the highest boron (B)‐efficiency index of the shoot (BEI_shoot_) score is given, along with average BEI_shoot_ scores for each allele ±SD. FW, fresh weight.

We assessed the effects of allelic variation in the lead SNP for BEI_shoot_ within the defined QTL on chromosome 4 (M4_15973361). Across the whole population, 7% of the accessions (13 of 185) possessed the less common AA allele, rising to 32% of accessions in the 90^th^ percentile for BEI_shoot_. In addition to BEI_shoot_ (*P* < 0.0001), significant allelic effects were identified for the ratios of trait performance under B‐deficient compared to B‐sufficient conditions of shoot DW (*P* < 0.0001), PLA at day 16 (*P* < 0.0001), BEI_root_ (*P* = 0.0007), ALRL (*P* = 0.0104) and TLRL (*P* = 0.0003; Fig. 9d). This suggests either that variation in a single locus confers both root and shoot resilience to B‐deficient conditions, or that such allelic variation confers, for example, root resilience to B deficiency, which in turn contributes to shoot resilience through better B access or B‐uptake efficiency. Additionally, we performed a protein haplotype analysis in the amino acid sequences of the candidate genes for B efficiency, BOR7 (Fig. S21), PME44 (Fig. S22) and LIME1 (Fig. S23) to test whether accession‐specific amino acid sequence variations associate with B efficiency. Across 104 accessions for which haplotype data were available in the SNP★ar tool used, 10 protein haplotypes were identified in BOR7 (Fig. S21a). Accessions possessing the haplotype BOR7g exhibited 66% higher BEI_shoot_ scores, on average, compared to the reference protein haplotype (BOR7a; Fig. S21b), whilst accessions possessing BOR7j exhibited 131% higher BEI_shoot_ scores (Fig. S21b) and 115% higher BEI_root_ scores (Fig. S21c), on average, compared to the reference haplotype. Eight protein haplotypes were identified for PME44 (Fig. S22a), including one which was associated with 129% higher BEI_shoot_ scores (PME44c; Fig. S22b). Meanwhile, 13 protein haplotypes were identified for LIME1 (Fig. S23a), one of which was associated with 246% higher BEI_shoot_ scores (Fig. S23b) and 143% higher BEI_root_ scores (LIME1e; Fig. S23c) and another of which was associated with 190% higher BEI_shoot_ scores (LIME1g; Fig. S23b). These therefore are strong candidates for which allelic variation within their amino acid sequences may contribute to variation in B efficiency in Arabidopsis.

Interestingly, 13 of the 19 most B‐efficient accessions based on BEI_shoot_ did not possess the more B‐efficient allele at locus M4_15973361. This indicates that whilst this locus may contribute to B efficiency, other loci are also of importance. Indeed, several other marker‐trait associations were identified for BEI_shoot_, the most highly associated of which was identified on chromosome 2 (M2_07055486; Fig. 9a) and which was also significantly associated with shoot FW (Fig. S17). A gene contributing to the elongation of fatty acids which are precursors for the synthesis of cuticular waxes, aliphatic suberins and membrane lipids (AT2G16280; Kim *et al*., 2013), and which therefore could contribute to maintaining plant tissue integrity, was identified 2.5 kb upstream of the associated SNP. The marker M2_00667596, which associated with shoot FW (Fig. S17), is 12.6 kb downstream of AT2G02470, which acts as a regulator of root hair formation under phosphate stress (Chandrika *et al*., 2013). The trichome birefringence‐like genes AT3G14850 and AT5G19160, which are related to genes involved in the synthesis and deposition of secondary wall cellulose, were 10.2 kb downstream of M3_04985191 (associated with BEI_shoot_) and 7.5 kb upstream of M5_06440519 (associated with shoot FW), respectively (Fig. S17). These each form additional B‐efficiency‐conferring candidate genes. In summary, whilst a single locus on chromosome 4 appears to promote both root and shoot tolerance to B limitation, it is clear that B efficiency is a complex trait to which multiple, independent loci contribute.

## Discussion

### Boron efficiency is rare amongst Arabidopsis accessions and does not correlate with tolerance to other abiotic stresses

Nutrient efficiency in plants can be defined in numerous ways. ‘Agronomic efficiency’ refers to plant production of harvestable product per unit of nutrient applied (Brouder & Volenec, 2023) and is most relevant for field‐scale studies of crop species. ‘Recovery efficiency’ is defined as the proportion of nutrient taken up compared to that which is theoretically available. Meanwhile, ‘physiological efficiency’ describes the ability of plants to convert the acquired nutrients into biomass and is a product of nutrient partitioning and the environment. In this study, we analysed B limitation responses of 185 highly diverse Arabidopsis accessions that already vary widely in their biomass accumulation in nutrient replete conditions. Identification of a suitable B‐efficiency metric that considers not only the performance of accessions in B‐deficient conditions compared to the population mean but also compared to the maximum genotypic potential of each accession was therefore far from trivial. Our identification of B‐efficient accessions, as defined as those with the highest population‐wide BEI_shoot_ and BEI_root_ scores, is mostly based on a measure of physiological efficiency, and specifically on the ability of an accession to maintain its native biomass accumulation as observed in nutrient‐replete conditions. This decision was taken based on multiple previous studies (Xue *et al*., 1998; Stangoulis *et al*., 2000a,b) which demonstrated that the vegetative responses of different genotypes to B deficiency were a good indicator of genotypic variation in B efficiency.

Of the 185 accessions studied here, we classified the 19 which were within the 90^th^ BEI_shoot_ percentile as B efficient. However, only three accessions, Var2‐1, Hn‐0 and Tha‐1, maintained both PLA at 16 DAS and shoot DW above 90% of their level under B‐sufficient conditions when exposed to B deficiency. We therefore consider B efficiency to be rare in Arabidopsis. Boron efficiency is also rare in *B. napus* and wheat (Anantawiroon *et al*., 1997; Pommerrenig *et al*., 2018). Most natural soils in the world are not B limited. Instead, B deficiency is mostly associated with environmental conditions such as longer‐lasting rainfalls (causing B leaching) or droughts (causing limited B flux towards roots) which affect B availability only spatiotemporally (Gupta *et al*., 1985; Shorrocks, 1997; Brdar‐Jokanović, 2019; Landi *et al*., 2019). Therefore, there may not have been strong selection pressures to develop B‐deficiency tolerance in most natural plant populations.

The mechanisms controlling B efficiency appear to be unique to B in Arabidopsis, as opposed to mechanisms generally conferring tolerance to hydromineral stress conditions. The accession we identified with the highest BEI_shoot_, Var2‐1, was not among the most Zn‐efficient accessions in the study of Campos *et al*. (2017). On the contrary, the most Zn‐efficient accessions, Tsu‐0 and Col‐0, both had BEI_shoot_ scores within the bottom 20^th^ percentile. In addition, the accessions Tsu‐0, Mt‐0, Oy‐0, Col‐0, Ct‐1 and Stw‐0, which have been implicated in exhibiting tolerance to N deficiency or identified as ideotypes for yield and seed quality (Chardon *et al*., 2012), were all far from B‐deficiency tolerant in our study. There were also no clear correlations between B efficiency and N‐limitation tolerance among accessions included both here and in the study of Ikram *et al*. (2012), nor between B efficiency and mild drought tolerance (Chen *et al*., 2021). However, Cvi‐0, which was one of the most B‐efficient accessions identified here, was also identified as one of the most drought‐tolerant accessions by Bouchabke *et al*. (2008). Thus, whilst isolated examples of tolerance to multiple abiotic stresses may exist within Arabidopsis, tolerance to B limitation generally does not relate to other individual mineral stresses.

No differences in photosynthetic operating efficiency between B‐efficient and B‐inefficient accessions were observed here. By contrast, Fe limitation has been linked to reduced photosynthetic efficiency as well as increased transpiration (El Amine *et al*., 2023), and K deficiency to both reduced photosynthetic efficiency and transpiration in soybean (Wang *et al*., 2015). Iron deficiency is also linked to reduced photosystem II efficiency in Arabidopsis (Robe *et al*., 2020).

### Lateral roots contribute most to B efficiency among root traits

Boron limitation induces an instantaneous detrimental effect on primary root elongation, root system architecture, mitotic activity of the primary root meristem and root functioning (Abreu *et al*., 2014; Liu *et al*., 2021). Primary root length was the most negatively impacted root trait measured in the present study, probably linked to reduced cell elongation, since B crosslinks cell wall pectin molecules and B starvation results in cell wall malformations (Wimmer *et al*., 2020). In a previous study, the PRL of Col‐0 was up to 39% shorter in an environment with no added B compared to control conditions, whilst LRD and growth slightly increased (Gruber *et al*., 2013). In support of the latter, 38 accessions here increased their ALRL under B deficiency, suggesting a response to low B that favours root foraging. In many soils, more B is found in the topsoil, where it is associated with soil organic matter (Gupta *et al*., 1985). Increasing LRL may therefore represent a favourable strategy to acquire more B where soil concentrations are low. Interestingly, the accession Var2‐1, which had the highest BEI_shoot_, displayed the second longest PRL under B‐deficient growth conditions but the second shortest TLRL among accessions here. This suggests that in Var2‐1, lateral roots did not contribute to B efficiency, therefore revealing variation in B‐efficiency strategies between accessions. The observed plasticity in root responses suggests that distinct root responses exist in Arabidopsis and that results obtained for the reference accession, Col‐0, may not always reflect species‐wide behaviours.

A previous study characterized Ler as B efficient and Col‐4 as B inefficient (Zeng *et al*., 2007). Whilst Ler‐1 did not belong to the accessions which we defined to be B‐efficient, Ler‐1 was still in the 75^th^ percentile of BEI_shoot_ scores. Meanwhile, Col‐0 was one of the most B‐sensitive accessions here. However, whilst our study suggested a higher TRL in B‐efficient compared to B‐inefficient accessions, in the study of Zeng *et al*. (2007), the root DW of the B‐inefficient Col‐4 was, independent of the B status, higher than that of Ler. This once more suggests that different adaptations leading to B efficiency exist within the Arabidopsis gene pool, or that different accessions can have very different B efficiencies even if closely related.

### Boron efficiency evolved independently in different regions

Our genetic and geographic clustering analyses indicate that not all identified B‐efficient accessions originated from a single B‐efficient ancestor. Hotspots for B efficiency exist, most notably in Scandinavian countries. The B‐efficient accessions Var2‐1, Es‐0 and Eden‐1 from Sweden, Finland and Sweden, respectively, cluster together both genetically and geographically. Three of the remaining four B‐efficient accessions of clade 6 also originated from Europe, and one from Cape Verde. Clade 3 contains a further six European accessions defined here as either B efficient or B‐intermediate efficient, originating from Switzerland (3), France (2) or Portugal (1). Interestingly, clade 3 additionally includes Alc‐0 and Pa‐1 that were amongst the most B‐deficiency sensitive accessions identified. Indeed, each of the seven clades containing accessions in the 90^th^ percentile for BEI_shoot_ (Fig. 7a) also contains B‐sensitive accessions, and therefore B efficiency appears to have evolved independently in each case. A similar spatially localised evolution of *Brassicaceae* tolerance to mineral stress conditions has been described in the evolution of Cd or Zn hyperaccumulators (Hanikenne *et al*., 2008). At the same time, the existence of highly genetically related B‐efficient accessions across wider geographic ranges indicates relatively few evolutionary events leading to B efficiency, which spread out over time before being enriched through evolutionary selection within localised regions of low B availability.

### In most accessions, B efficiency is neither associated with increased B transporter expression nor B transporter sequence variation

Shoot B concentrations of the B‐efficient accessions were on average 41% higher than in the B‐inefficient accessions under B‐deficient growth conditions. Remarkably, on average, no differences in the expression of the B transporter genes *AtNIP5;1*, *AtNIP6;1*, or *AtBOR1*, which are known, in contrast to other B transporters such as BOR2 or BOR7, to impact on Arabidopsis shoot B content, were observed between B‐efficient and B‐inefficient accessions. This is in contrast to results observed within a population of 19 accessions, within which Zn‐deficiency tolerant accessions exhibited increased expression of Zn transporters when grown under Zn deficiency (Campos *et al*., 2017). This is also in contrast to results obtained in *B. napus*, in which *BOR1* and *NIP5;1* orthologues were strongly linked to vegetative and reproductive performance in B‐efficient lines (Hua *et al*., 2016; Zhang *et al*., 2017; Liu *et al*., 2024). This may either suggest that B transporters play a larger role in B efficiency after the onset of flowering or that different B‐efficiency mechanisms exist in the Arabidopsis population studied here. That shoot B contents of plants grown in B‐deficient conditions were on average 552% higher in B‐efficient than B‐inefficient accessions supports the latter hypothesis. Being able to maintain a bigger root system could contribute to B‐acquisition in B‐efficient accessions. Indeed, the B‐efficient accessions produced 13.9% longer root systems under B deficiency than the other accessions, on average (Table S1), and TRL correlated with shoot B content (Pearson *r* = 0.507, *P* < 0.001; Fig. 4f).

Additional mechanisms besides effective B uptake must contribute to the B‐deficiency tolerance, as even B‐efficient accessions suffered an 84% drop in shoot B concentration under B deficiency, compared to an 87% drop in B‐inefficient accessions. In addition, several accessions with similar shoot B concentrations when grown in B‐deficient conditions exhibited completely different tolerances to B‐deficiency (Fig. 4e). In support of this, the Arabidopsis mutant *lbt* was shown to be much more B‐deficiency tolerant than wild‐type Col‐0, without any observed differences in shoot B concentrations (Huai *et al*., 2018). The higher shoot K concentration observed in many B‐efficient accessions (Fig. S5) may indicate an osmotic adjustment component to B efficiency. Indeed, Arabidopsis K transporter knock‐out mutants exhibited decreased tolerance to water deficit (Osakabe *et al*., 2013). Further experimental work will be required to confirm a possible role of K nutrition in B‐deficiency tolerance.

### Candidate genes for B efficiency in Arabidopsis include a B transporter and genes involved in maintaining structural cell wall organisation

Eleven markers were significantly associated with BEI_shoot_ or B‐limitation resilience of shoot FW or TLRL. For each marker, we designated the marker allele with the highest average BEI_shoot_ as a B‐efficiency allele (Table 1). No single accession possessed B‐efficiency alleles at all of these loci. However, the more B‐efficiency alleles an accession had, the more likely it was to be B efficient. All 19 of the most B‐efficient accessions based on BEI_shoot_ had at least three B‐efficiency alleles, whereas none of the least B‐efficient accessions had more than three, and five of these had none. Four accessions, namely Var2‐1, Yo‐0, Hn‐0 and Tha‐1, had at least eight B‐efficiency alleles. These were all very highly B‐efficient accessions with BEI_shoot_ scores of at least 0.78. Boron efficiency is therefore a complex trait, requiring suitable alleles at multiple loci for the highest tolerance to B limitation. Indeed, tolerance to low B stress was also described to be a quantitative trait following an assessment of 101 recombinant inbred lines derived from only two parent accessions (*Ler* and Col‐4; Zeng *et al*., 2007), and therefore it is not surprising that we have identified multiple quantitative loci contributing to B efficiency in a population of 185 Arabidopsis accessions. Seven of the B‐efficiency alleles identified here were enriched within phylogenetic clade 6, which had the highest incidence of B‐efficient accessions. One marker on chromosome 3 (M3_04985191) and three on chromosome 4 (M4_15783156, M4_15973361 and M4_16040935) were more than five‐fold enriched in this clade compared to their incidence across the whole population. Notably, Tamm‐2 and Tamm‐27, which were grouped into clade 6 despite both being relatively B‐sensitive accessions, each had the B‐sensitive alleles at these marker positions. This may indicate that B‐efficiency evolved in this clade after it diverged from the rest of the population and then proliferated within it.

We identified a high‐confidence QTL for B efficiency on chromosome 4. The existence of co‐localising marker‐trait associations for both shoot and root resilience to B limitation suggests either that a single molecular mechanism controls both root and shoot B efficiency, or that maintaining root productivity and exploration under B limitation leads to a more resilient shoot. The two most promising candidate genes identified within this QTL are *BOR7* and *PME44*. BOR7 is a close relative of BOR1, which is a B‐efflux transporter involved in B export from pericycle cells into the root stele (Takano *et al*., 2002). As B efficiency was not linked to B concentration in this study, BOR7 may fine‐tune spatiotemporal allocation of B to specific cell types or cell wall regions rather than influencing the total B accumulation of shoots or roots. Yet, *BOR7* was defined as selectively expressed in pollen (Becker *et al*., 2003). However, we previously observed that two *Brassica napus* orthologues of *BOR7* were relatively stably expressed across multiple shoot tissues and growth stages, in both B‐sufficient and B‐deficient conditions (Verwaaijen *et al*., 2023), and therefore *BOR7* remains a worthy candidate of further study. In particular, two protein haplotypes of BOR7 were identified to be associated with higher average BEI_shoot_ scores than the reference haplotype, one of which was also associated with higher BEI_root_ scores (Fig. S21). These therefore form candidate B‐efficiency haplotypes of BOR7. So far, this analysis was limited to the 104 accessions shared between this study and the SNP★ar tool. Expanding this analysis to additional accessions that share these haplotypes, using the 15 and 4 accessions with the candidate B‐efficient BOR7 haplotypes as predictive accessions for general haplotype responses to B deficiency, will be an important next step towards confirming the contribution of these haplotypes to B efficiency. *PME44* is a member of the pectin methylesterase family, members of which play a key role in modifying and maintaining the mechanical strength of primary cell walls. Boron itself has a major role in cross‐linking RG‐II monomers in the pectin fraction of the primary cell wall, maintaining cell wall structural integrity. It is therefore a reasonable hypothesis that allelic or expressional variation in PMEs might be able to offset the negative structural impacts of B deficiency. Indeed, a protein haplotype of PME44 was associated with higher BEI_root_ scores (Fig. S22). In addition, mutations in *PME44* led to increased susceptibility of Arabidopsis plants to *Pseudomonas syringae* (Bethke *et al*., 2014), indicating that despite likely genetic redundancy within the *PME* family, this member is likely to play an active role in cell wall integrity, and is a major candidate gene for the variation in B‐deficiency tolerance observed in this study.

### Conclusion

Our analyses provide experimental evidence for phenotypic plasticity in root and shoot responses to B deficiency in a diverse panel of Arabidopsis. We have identified highly B deficiency‐tolerant accessions that share general root and shoot resilience to B limitation. Across accessions in B‐limited conditions, a vigourous root system and in particular the ability to maintain lateral root length is associated with shoot biomass accumulation. Variation in expression and amino acid sequence of major B transport proteins was not associated with either B uptake or B efficiency, and the small differences in shoot B concentration between B‐efficient and B‐inefficient accessions indicate a low B requirement of B‐efficient accessions. Close phylogenetic clustering between individual B‐efficient and B‐inefficient accessions suggests that small genetic changes can have a large effect on B efficiency. Genome‐wide association analyses identified a locus on chromosome 4 associated with both shoot and root trait resilience to B limitation, either suggesting a shared molecular basis to B efficiency in these tissues or that a B‐efficient root system contributes to a B‐resilient shoot system. Our detailed dataset represents the basis for successive molecular, phenotypic and metabolic analyses to further dissect B‐efficiency mechanisms in Arabidopsis.

## Competing interests

None declared.

## Author contributions

GPB conceived the study. GPB, MDB and TDA wrote the first paper draft. All authors prepared, read and approved the final manuscript. The B‐deficiency tolerance screens in *in vitro* and *in terra* growth conditions and related experiments were conducted and supervised mainly by MDB and GPB, but with the help of all other authors. Proteoform analysis was conducted by GPB, SK and TDA. Phylogenetic and bioinformatic analysis was performed by TDA. Genome‐wide association analyses were performed by RCM and TDA. Work related to the automated imaging/phenotyping and subsequent analyses has been performed by AJ, MDB, TDA, TA and GPB. RCM processed and amplified the Arabidopsis accessions and provided the genotypic data. HT performed and analysed the photosynthetic operating efficiency experiments. TDA, GPB, MDB, NW, TA and AJ analyzed and interpreted the data.

## Disclaimer

The New Phytologist Foundation remains neutral with regard to jurisdictional claims in maps and in any institutional affiliations.

## Supporting information


**Fig. S1** Visual comparison of the response of the Arabidopsis accession Col‐0 and the boron (B) transporter mutant *nip5;1* to B deficiency.
**Fig. S2** Correlations between Arabidopsis shoot area and shoot dry weight across different measurement days and imaging methods.
**Fig. S3** Range in photosynthetic operating efficiencies of Arabidopsis accessions in both boron (B)‐deficient and B‐sufficient growth conditions.
**Fig. S4** Range of measured projected leaf area at 16 d after sowing, shoot fresh weight and shoot dry weight across 185 Arabidopsis accessions.
**Fig. S5** Shoot concentrations of plant macro‐nutrients of boron (B)‐efficient and B‐inefficient Arabidopsis accessions in B‐sufficient or B‐deficient growth conditions.
**Fig. S6** Shoot concentrations of plant micro‐nutrients of boron (B)‐efficient and B‐inefficient Arabidopsis accessions in B‐sufficient or B‐deficient growth conditions.
**Fig. S7** Shoot boron (B) contents per plant of B‐efficient and B‐inefficient Arabidopsis accessions grown in B‐sufficient or B‐deficient growth conditions.
**Fig. S8** Correlation between root architectural traits and shoot boron (B) concentration and content under B‐deficient conditions.
**Fig. S9** Correlations between Arabidopsis biomass traits measured in the soil‐based automated phenotyping experiment and the Phytagel‐based agar plate experiment.
**Fig. S10** Variation in boron (B) transporter expression of B‐efficient and B‐inefficient Arabidopsis accessions in B‐sufficient or B‐deficient growth conditions.
**Fig. S11** Correlations of boron (B) transporter expression against BEI_shoot_, and shoot B concentration and content under B‐deficient conditions.
**Fig. S12** Association between the amino acid sequence haplotypes of the Arabidopsis boron (B) transport proteins AtNIP5;1, AtNIP6;1 and AtBOR1 and B efficiency and B uptake.
**Fig. S13** Full phylogenetic dendrogram of 185 Arabidopsis accessions.
**Fig. S14** Trait reduction pipeline for *k*‐means analysis.
**Fig. S15** Correlations table for all 38 Arabidopsis boron‐dependent shoot and root traits included in *k*‐means analysis.
**Fig. S16** Correlations between root architectural traits and shoot dry weight of plants grown in boron‐deficient conditions.
**Fig. S17** Genome‐wide SNP associations with selected shoot trait ratios between boron (B)‐deficient and B‐sufficient conditions.
**Fig. S18** Genome‐wide SNP associations with selected root trait ratios between boron (B)‐deficient and B‐sufficient conditions.
**Fig. S19** Quantile‐quantile (QQ) plots comparing expected with observed ‐log_10_ *p* values obtained from shoot trait‐based genome‐wide association analyses.
**Fig. S20** Quantile‐quantile (QQ) plots comparing expected with observed ‐log_10_ *p* values obtained from root trait‐based genome‐wide association analyses.
**Fig. S21** Association between the amino acid sequence haplotypes of the Arabidopsis boron (B) transport protein BOR7 (AT4G32510) and B efficiency.
**Fig. S22** Association between the amino acid sequence haplotypes of the Arabidopsis protein PME44 (AT4G33220) and boron (B) efficiency.
**Fig. S23** Association between the amino acid sequence haplotypes of the Arabidopsis protein LIME1 (AT4G33110) and boron (B) efficiency.
**Methods S1** Detailed experimental methodology.

**Table S1** Accessions used in Arabidopsis root and shoot experiments, sampling location, clustering based on *k*‐means analysis and analysis of phylogenetic relationships, and mean trait data for all phenes on all measurement days.
**Table S2** Relative SD of trait data for all measurement days from the Arabidopsis automated phenotyping experiment.
**Table S3** Description of parameters measured within the Arabidopsis automated phenotyping experiment.
**Table S4** Randomised block design used for carriers in the Arabidopsis automated plant phenotyping system. ‘XXX’ refers to accessions that were removed from subsequent analysis due to poor germination or missing data.
**Table S5** RT‐qPCR primer sequences.
**Table S6** Results of the genetic association analyses.
Please note: Wiley is not responsible for the content or functionality of any Supporting Information supplied by the authors. Any queries (other than missing material) should be directed to the *New Phytologist* Central Office.

## Data Availability

The data that support the findings of this study are available in Tables S1, S2, S6.
