# Supplementary material for: Arabidopsis thaliana exhibits wide within‐species variation in tolerance to boron limitation and root and shoot trait resilience associate with a pleiotropic locus
Source: New Phytol. 2025 Sep 15;248(5):2444–65. doi: 10.1111/nph.70570 (PMC12589721; doi:10.1111/nph.70570)
Supplement: Supplementary file 1 — Fig. S1 Visual comparison of the response of the Arabidopsis accession Col‐0 and the boron (B) transporter mutant nip5;1 to B deficiency. Fig. S2 Correlations between Arabidopsis shoot area and shoot dry weight across different measurement days and imaging methods. Fig. S3 Range in photosynthetic operating efficiencies of Arabidopsis accessions in both boron (B)‐deficient and B‐sufficient growth conditions. Fig. S4 Range of measured projected leaf area at 16 d after sowing, shoot fresh weight and shoot dry weight across 185 Arabidopsis accessions. Fig. S5 Shoot concentrations of plant macro‐nutrients of boron (B)‐efficient and B‐inefficient Arabidopsis accessions in B‐sufficient or B‐deficient growth conditions. Fig. S6 Shoot concentrations of plant micro‐nutrients of boron (B)‐efficient and B‐inefficient Arabidopsis accessions in B‐sufficient or B‐deficient growth conditions. Fig. S7 Shoot boron (B) contents per plant of B‐efficient and B‐inefficient Arabidopsis accessions grown in B‐sufficient or B‐deficient growth conditions. Fig. S8 Correlation between root architectural traits and shoot boron (B) concentration and content under B‐deficient conditions. Fig. S9 Correlations between Arabidopsis biomass traits measured in the soil‐based automated phenotyping experiment and the Phytagel‐based agar plate experiment. Fig. S10 Variation in boron (B) transporter expression of B‐efficient and B‐inefficient Arabidopsis accessions in B‐sufficient or B‐deficient growth conditions. Fig. S11 Correlations of boron (B) transporter expression against BEIshoot, and shoot B concentration and content under B‐deficient conditions. Fig. S12 Association between the amino acid sequence haplotypes of the Arabidopsis boron (B) transport proteins AtNIP5;1, AtNIP6;1 and AtBOR1 and B efficiency and B uptake. Fig. S13 Full phylogenetic dendrogram of 185 Arabidopsis accessions. Fig. S14 Trait reduction pipeline for k‐means analysis. Fig. S15 Correlations table for all 38 Arabidopsis boron‐dep [file NPH-248-2444-s002.pdf]

New Phytologist **Supporting Information**

Article title: *Arabidopsis thaliana* exhibits wide within-species variation in tolerance to boron limitation and root and shoot trait resilience associate with a pleiotropic locus

Authors: Thomas D. Alcock, Manuela Désirée Bienert, Astrid Junker, Rhonda C. Meyer, Henning Tschiersch, Sreelekha Kudamala, Nicolaus von Wirén, Thomas Altmann, Gerd Patrick Bienert

Article acceptance date: 27 August 2025

The following Supporting Information is available for this article:

Fig. S1 Visual comparison of the response of the Arabidopsis accession Col-0 and the boron (B) transporter mutant *nip5;1* to B deficiency.

Fig. S2 Correlations between Arabidopsis shoot area and shoot dry weight across different measurement days and imaging methods.

Fig. S3 Range in photosynthetic operating efficiencies of Arabidopsis accessions in both boron (B)-deficient and B-sufficient growth conditions.

Fig. S4 Range of measured projected leaf area at 16 days after sowing, shoot fresh weight and shoot dry weight across 185 Arabidopsis accessions.

Fig. S5 Shoot concentrations of plant macro-nutrients of boron (B)-efficient and B-inefficient Arabidopsis accessions in B-sufficient or B-deficient growth conditions.

Fig. S6 Shoot concentrations of plant micro-nutrients of boron (B)-efficient and B-inefficient Arabidopsis accessions in B-sufficient or B-deficient growth conditions.

Fig. S7 Shoot boron (B) contents per plant of B-efficient and B-inefficient Arabidopsis accessions grown in B-sufficient or B-deficient growth conditions.

Fig. S8 Correlation between root architectural traits and shoot boron (B) concentration and content under B-deficient conditions.

Fig. S9 Correlations between Arabidopsis biomass traits measured in the soil-based automated phenotyping experiment and the Phytagel-based agar plate experiment.

Fig. S10 Variation in boron (B) transporter expression of B-efficient and B-inefficient Arabidopsis accessions in B-sufficient or B-deficient growth conditions.

Fig. S11 Correlations of boron (B) transporter expression against BEI_{shoot} and shoot B concentration and content under B-deficient conditions.

Fig. S12 Association between the amino acid sequence haplotypes of the Arabidopsis boron (B) transport proteins AtNIP5;1, AtNIP6;1 and AtBOR1 and B efficiency and B uptake.

Fig. S13 Full phylogenetic dendrogram of 185 Arabidopsis accessions.

Fig. S14 Trait reduction pipeline for *k*-means analysis.

Fig. S15 Correlations table for all 38 Arabidopsis boron-dependent traits included in *k*-means analysis.

Fig. S16 Correlations between root architectural traits and shoot dry weight of plants grown in boron-deficient conditions.

Fig. S17 Genome-wide SNP associations with selected shoot trait ratios between boron (B)-deficient and B-sufficient conditions.

Fig. S18 Genome-wide SNP associations with selected root trait ratios between boron (B)-deficient and B-sufficient conditions.

Fig. S19 Quantile-quantile (QQ) plots comparing expected with observed $-\log_{10}p$ values obtained from shoot trait-based genome-wide association analyses.

Fig. S20 Quantile-quantile (QQ) plots comparing expected with observed $-\log_{10}p$ values obtained from root trait-based genome-wide association analyses.

Fig. S21 Association between the amino acid sequence haplotypes of the Arabidopsis boron (B) transport protein BOR7 (*AT4G32510*) and B efficiency.

Fig. S22 Association between the amino acid sequence haplotypes of the Arabidopsis protein PME44 (*AT4G33220*) and boron efficiency.

Fig. S23 Association between the amino acid sequence haplotypes of the Arabidopsis protein LIME1 (*AT4G33110*) and boron efficiency.

Table S5 RT-qPCR primer sequences [note tables S1 – S4 and table S6 are included as supplementary spreadsheets]**.**

Methods S1 Detailed experimental methodology.

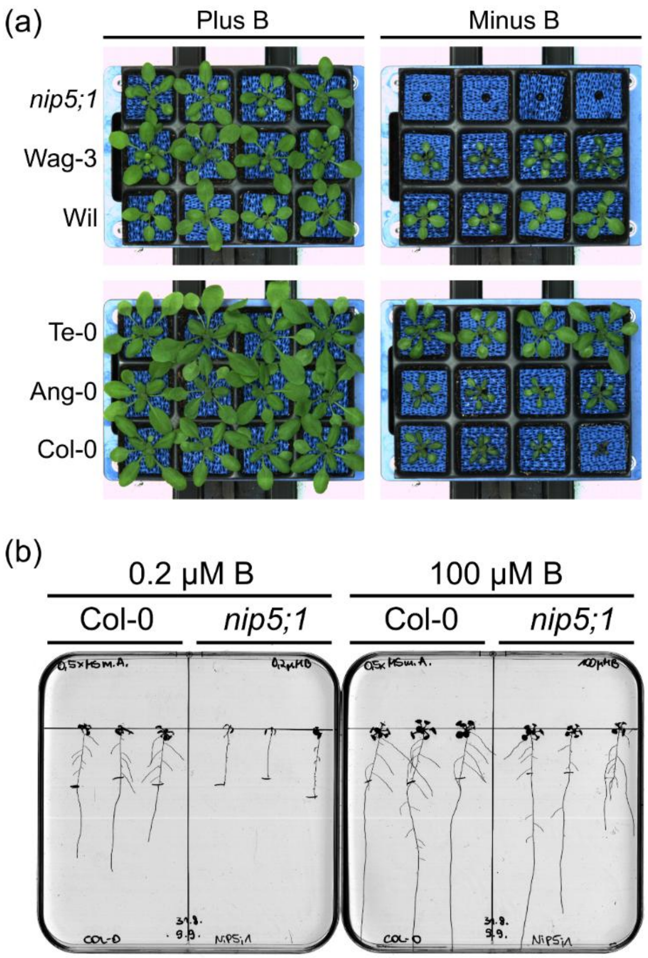

Fig. S1 Visual comparison of the response of the Arabidopsis accession Col-0 and the boron (B) transporter mutant *nip5;1* to B deficiency. (a) Images of plant growth systems used in the automated phenotyping system showing shoot performance of Col-0 and *nip5;1* to B-sufficient (2.5 mg B kg^{-1} soil; "Plus B") and B-deficient (< 0.1 mg B kg^{-1} soil; "Minus B") soil conditions. The other genotypes shown are included due to placement within the same growth tray. Images were taken 20 days after sowing. (b) Effects of B deficiency on Arabidopsis root morphology in Col-0 and *nip5;1* seedlings grown on Petri dishes containing a Phytagel-based growth media. The growth media contained modified half-strength Murashige and Skoog (MS) media either adequately (100 µM B) or inadequately (0.2 µM B) supplied with B. Scans show fifteen-day-old Arabidopsis accessions exposed to the contrasting B concentration treatments for 10 days.

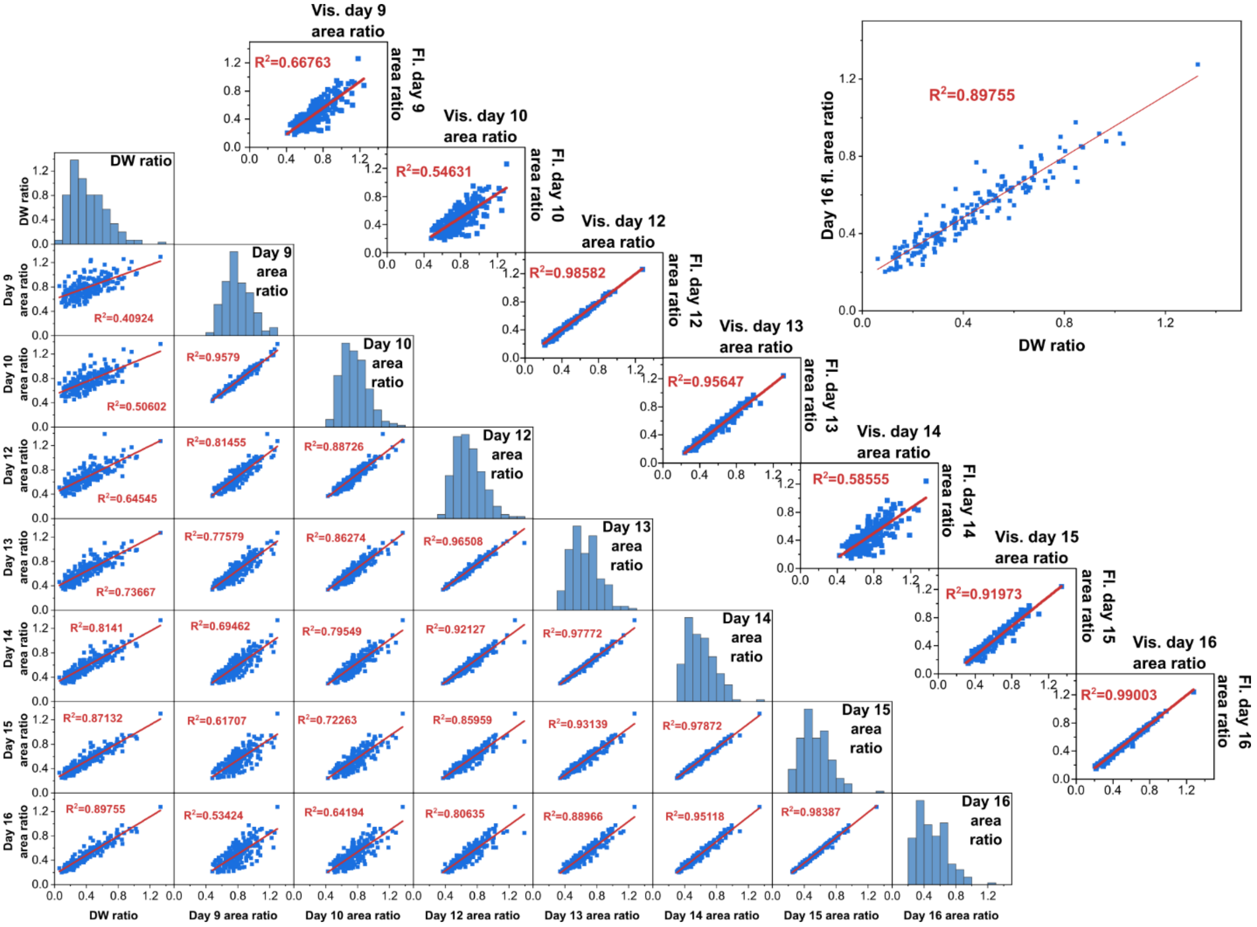

Fig. S2 Correlations between Arabidopsis shoot area (top view images) ratio between boron (B)-deficient (< 0.1 mg B kg-soil^{-1}) and B-sufficient (2.5 mg B kg-soil^{-1}) growth conditions on different imaging days (based on fluorescence imaging), between visible light and fluorescence-based imaging, and between area at day 16 (fluorescence imaging) and dry weight (DW) at experiment end (20 days after sowing). Values shown area squared Peason correlation coefficients (R^2).

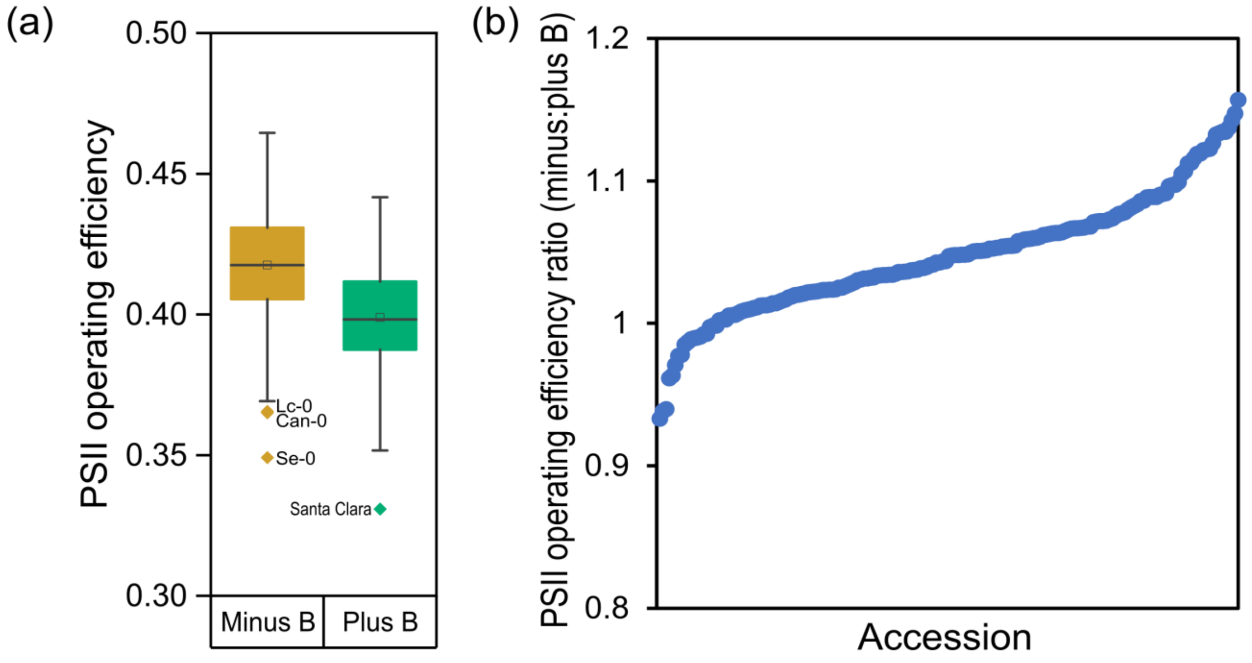

Fig. S3 Range in photosynthetic operating efficiency of 185 Arabidopsis accessions. Shown are data from plants grown in boron (B)-deficient (Minus B; < 0.1mg B kg-soil^{-1}) and B-sufficient (Plus B; 2.5 mg B kg-soil^{-1}) growth conditions (a), and the ratio in performance between growth conditions for each accession (b). PSII refers to photosystem II. Boxes in (a) represent the 25th to 75th percentiles and whiskers represent the range within 1.5 times the interquartile range (IQR) with extreme accessions indicated next to the respective data points. The lines and squares within the boxes in (a) represent the median and the mean, respectively.

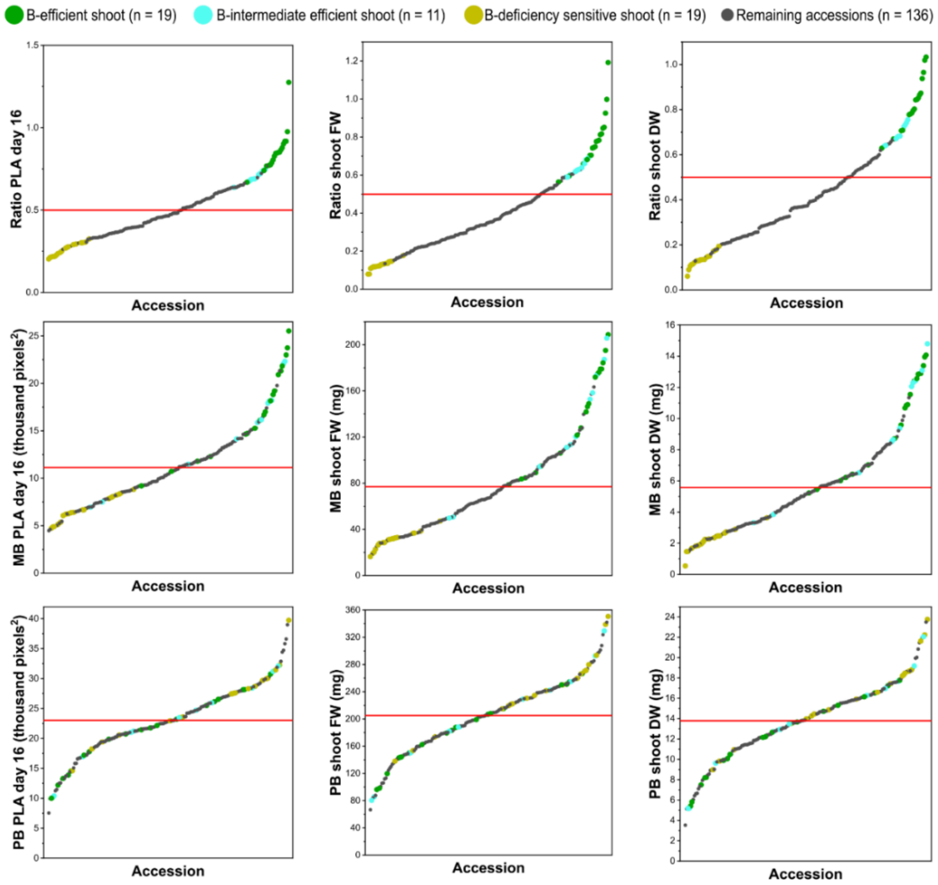

Fig. S4 Range of measured projected leaf area (PLA) at 16 days after sowing, shoot fresh weight (FW) and shoot dry weight (DW) across 185 Arabidopsis accessions grown in either boron (B)-sufficient (2.5 mg B kg^{-1} soil; "PB") or B-deficient (< 0.1 mg B kg^{-1} soil; "MB") soil-based conditions. Shown are the ratios of measurements on B-deficient compared to B-sufficient conditions for each accession for each of the three traits, and the actual trait measurements for each accession on B-deficient and B-sufficient conditions. In each panel, accessions are sorted in ascending order of measured trait values. The accessions of the 90th percentile of the B efficiency index of the shoot (BEI_{shoot}; B-efficient shoot), with BEI_{shoot} values over 0.400 (B-intermediate efficient shoot), and of the 10th percentile of BEI_{shoot} (B-deficiency sensitive shoot) are highlighted as shown in the figure legend. The horizontal red line in each panel represents the population-wide arithmetic mean for each trait or ratio shown.

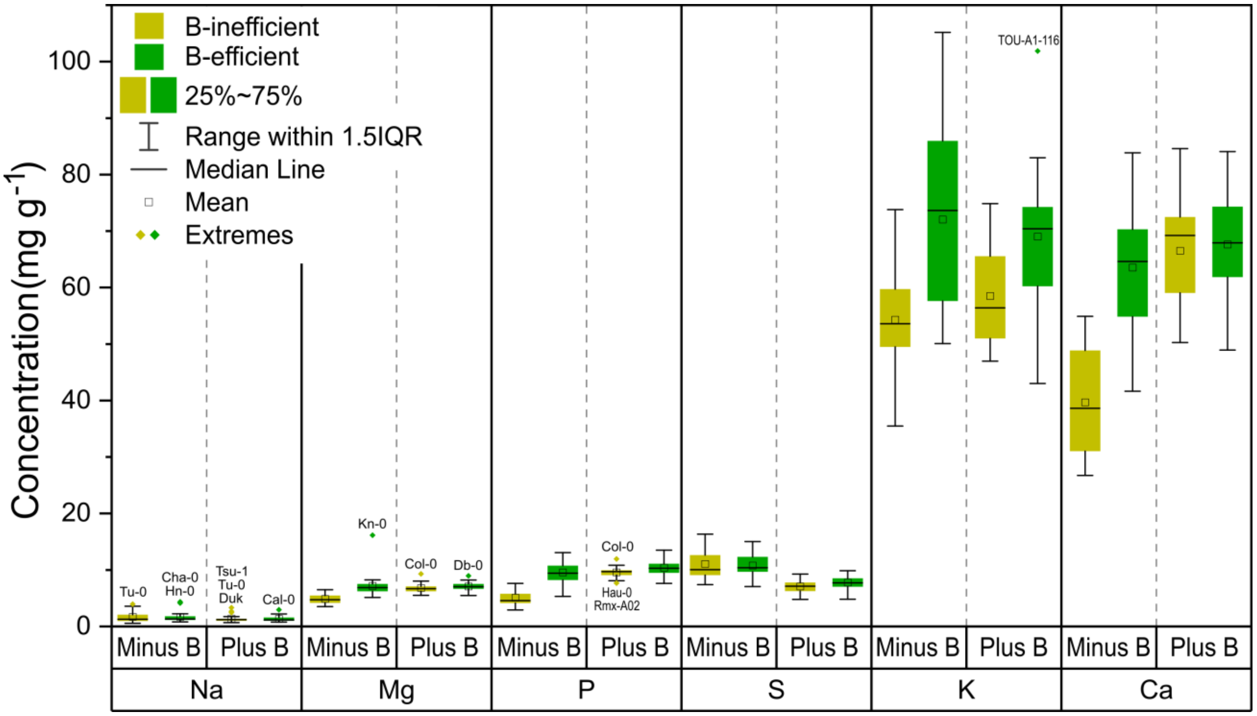

Fig. S5 Shoot concentrations of plant macro-nutrients of 43 Arabidopsis accessions (20 days old) considered boron (B)-efficient (22) or B-inefficient (21) in B-deficient (Minus B; < 0.1 mg B kg-soil^{-1}) or B-sufficient (Plus B; 2.5 mg B kg-soil^{-1}) growth conditions. Boxes represent the 25th to 75th percentiles and whiskers represent the range within 1.5 times the interquartile range (IQR) with extreme accessions indicated above or below the respective data points.

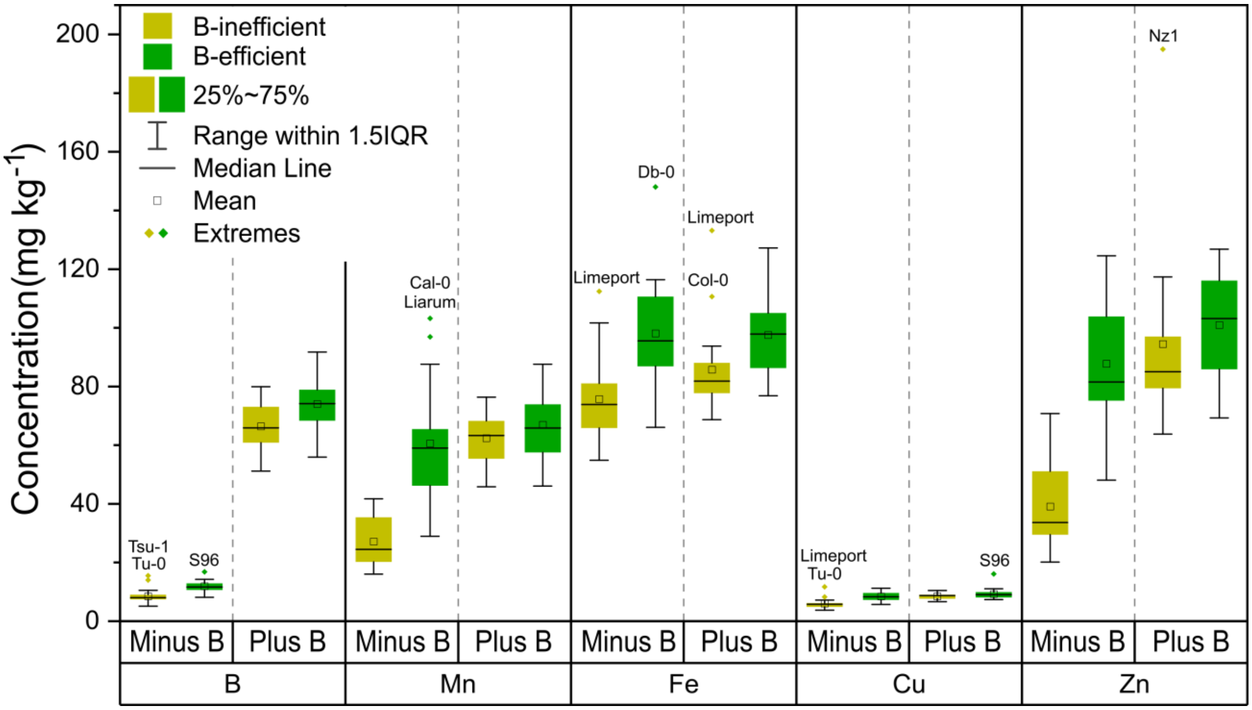

Fig. S6 Shoot concentrations of plant micro-nutrients of 43 Arabidopsis accessions (20 days old) considered boron (B)-efficient (22) or B-inefficient (21) in B-deficient (Minus B; < 0.1 mg B kg-soil^{-1}) or B-sufficient (Plus B; 2.5 mg B kg-soil^{-1}) growth conditions. Boxes represent the 25th to 75th percentiles and whiskers represent the range within 1.5 times the interquartile range (IQR) with extreme accessions indicated above the respective data points.

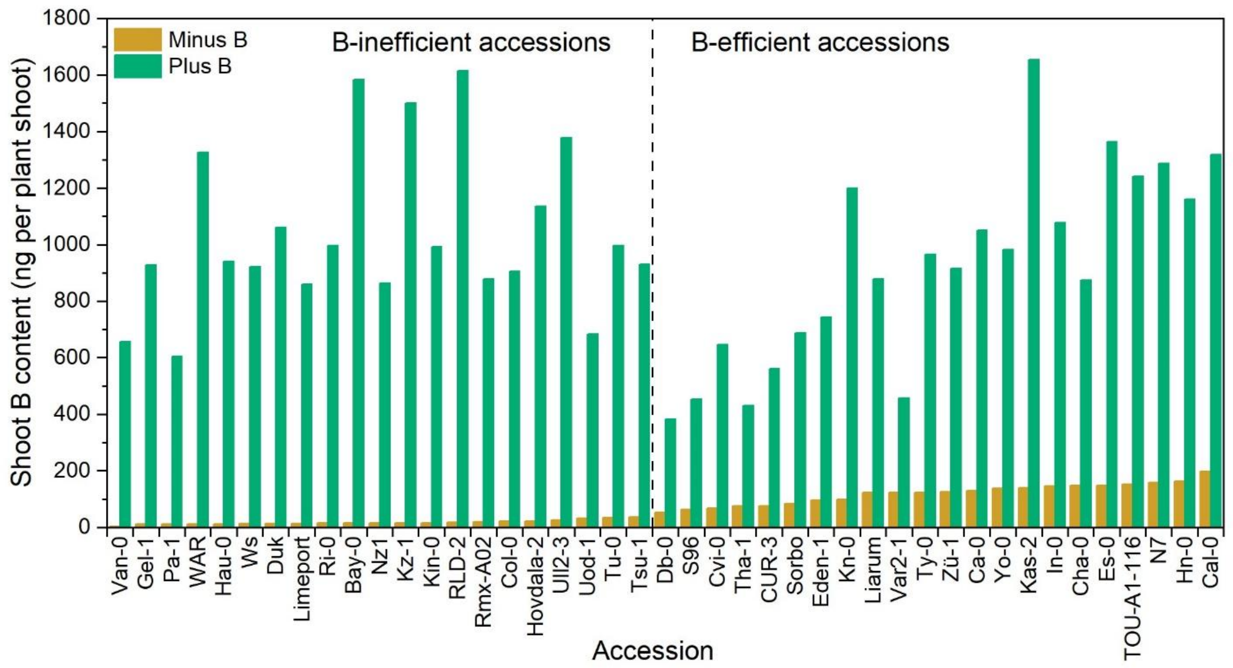

Fig. S7 Shoot boron (B) contents per plant of 43 Arabidopsis accessions considered B-efficient (22) or B-inefficient (21) grown in B-deficient (Minus B; < 0.1 mg B kg-soil^{-1}) or B-sufficient (Plus B; 2.5 mg B kg-soil^{-1}) growth conditions. Accessions are sorted from left to right in order of ascending B content in B-deficient growth conditions. B-inefficient (left-side) and B-efficient (right-side) accessions are separated by the dashed line.

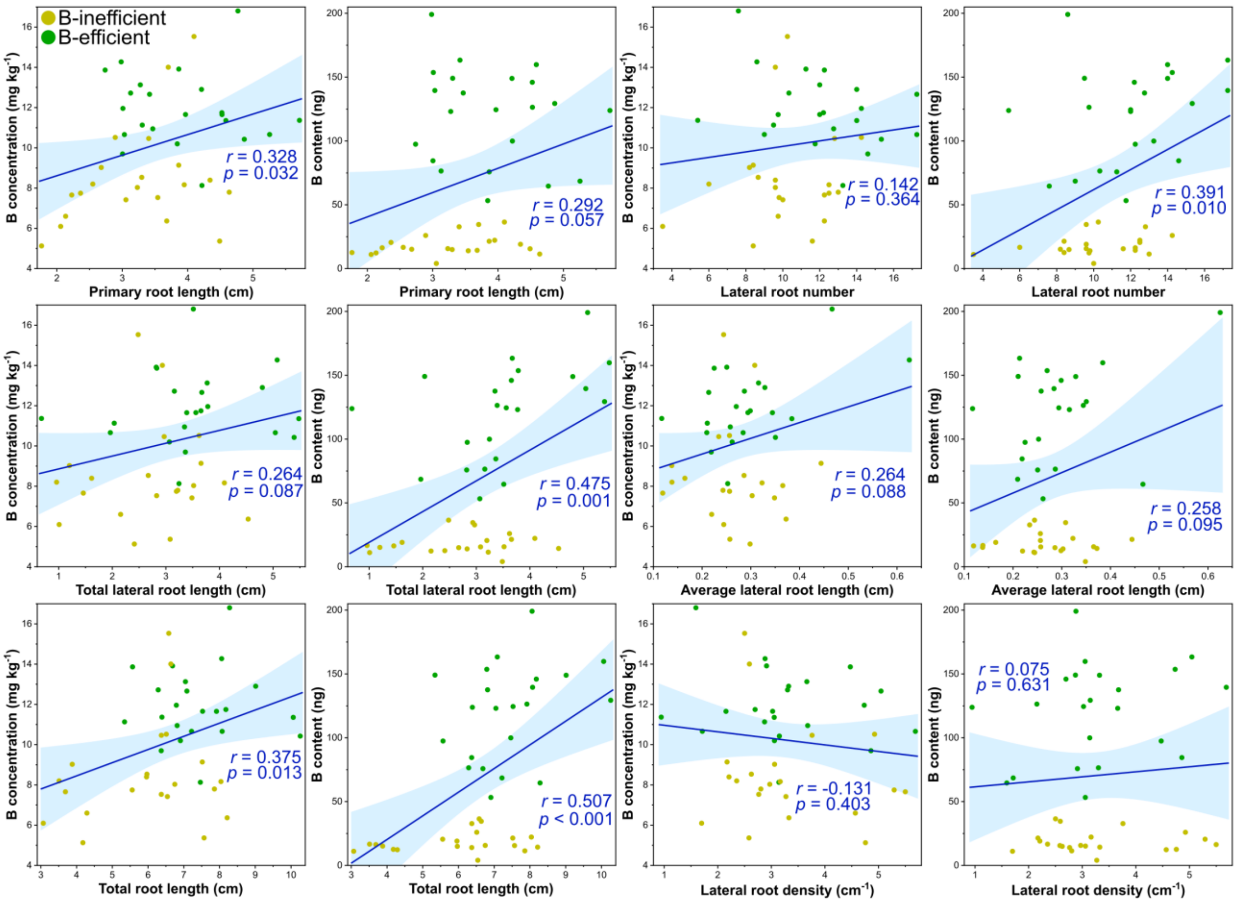

Fig. S8 Correlations between root architectural traits and shoot boron (B) concentration and content of 43 Arabidopsis accessions considered B-efficient (22) or B-inefficient (21) and grown under B-deficient conditions. Blue lines represent linear fit regression curves with 95% confidence interval bands shown. Pearson correlation coefficients (*r*) shown in each panel.

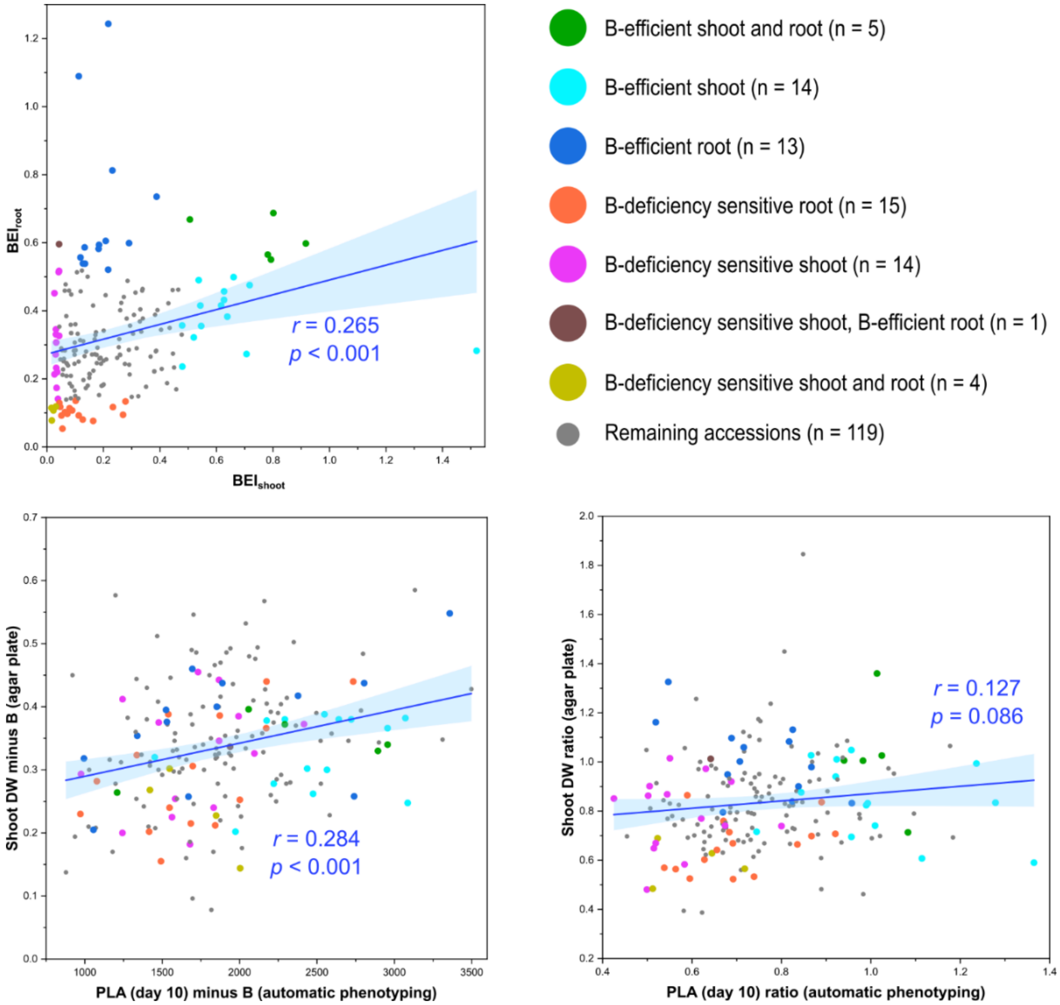

Fig. S9 Correlations between Arabidopsis biomass traits measured in the soil-based automated phenotyping experiment and the Phytagel-based agar Petri-dish experiment. Shown are correlations between the shoot and root boron (B) efficiency index scores (BEI_{shoot} and BEI_{root}), projected leaf area (PLA) at day 10 from the automated phenotyping experiment and shoot dry weight (DW) from the Petri-dish experiment under B-deficient conditions (minus B), and PLA at day 10 from the automated phenotyping experiment and shoot DW from the Petri dish experiment as ratios of trait scores under B-deficient compared to B-sufficient conditions. PLA at day 10 was selected as the most suitable biomass-related trait from the automated phenotyping experiment to compare with shoot DW from the Petri dish experiment, as shoot DW from the

Petri dish experiment was measured after plants had been exposed to the different B conditions for 10 days. Each dot represents a single accession, where accessions that were grouped into different B efficiency groupings based on BEI_{shoot} and/or BEI_{root} are colour-coded as summarised in the legend. Blue lines represent linear fit regression curves with 95% confidence interval bands shown. Pearson correlation coefficients (*r*) shown in each panel.

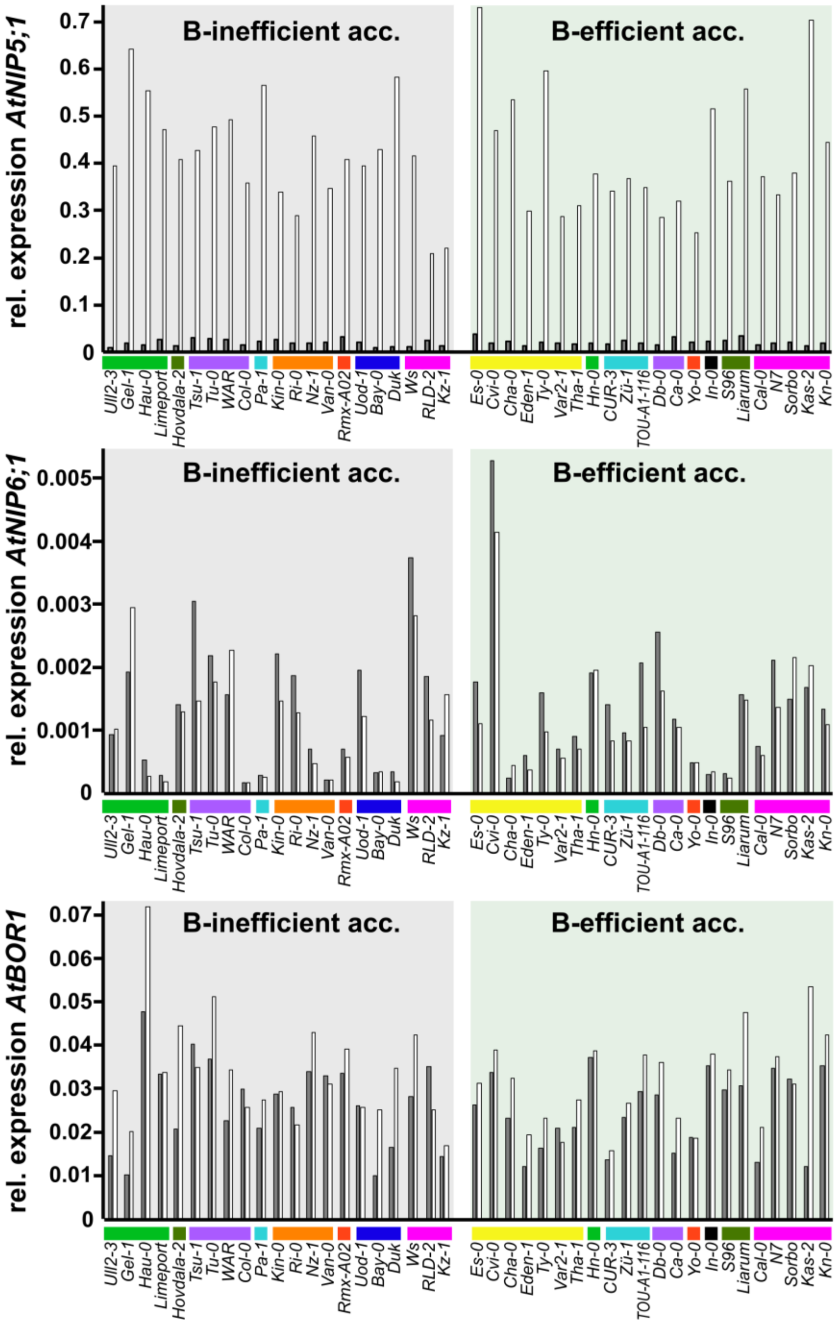

Fig. S10 Variation in boron (B) transporter (*NIP5;1*, *NIP6;1* and *BOR1*) relative expression levels in 15-day old roots of 43 Arabidopsis accessions considered B-efficient or B-inefficient grown in B-sufficient (grey bars; 100 µM B) or B-deficient (white bars; 0.2 µM B) growth conditions quantified by RT-qPCR analysis. Colours by accession names represent different phylogenetic clades.

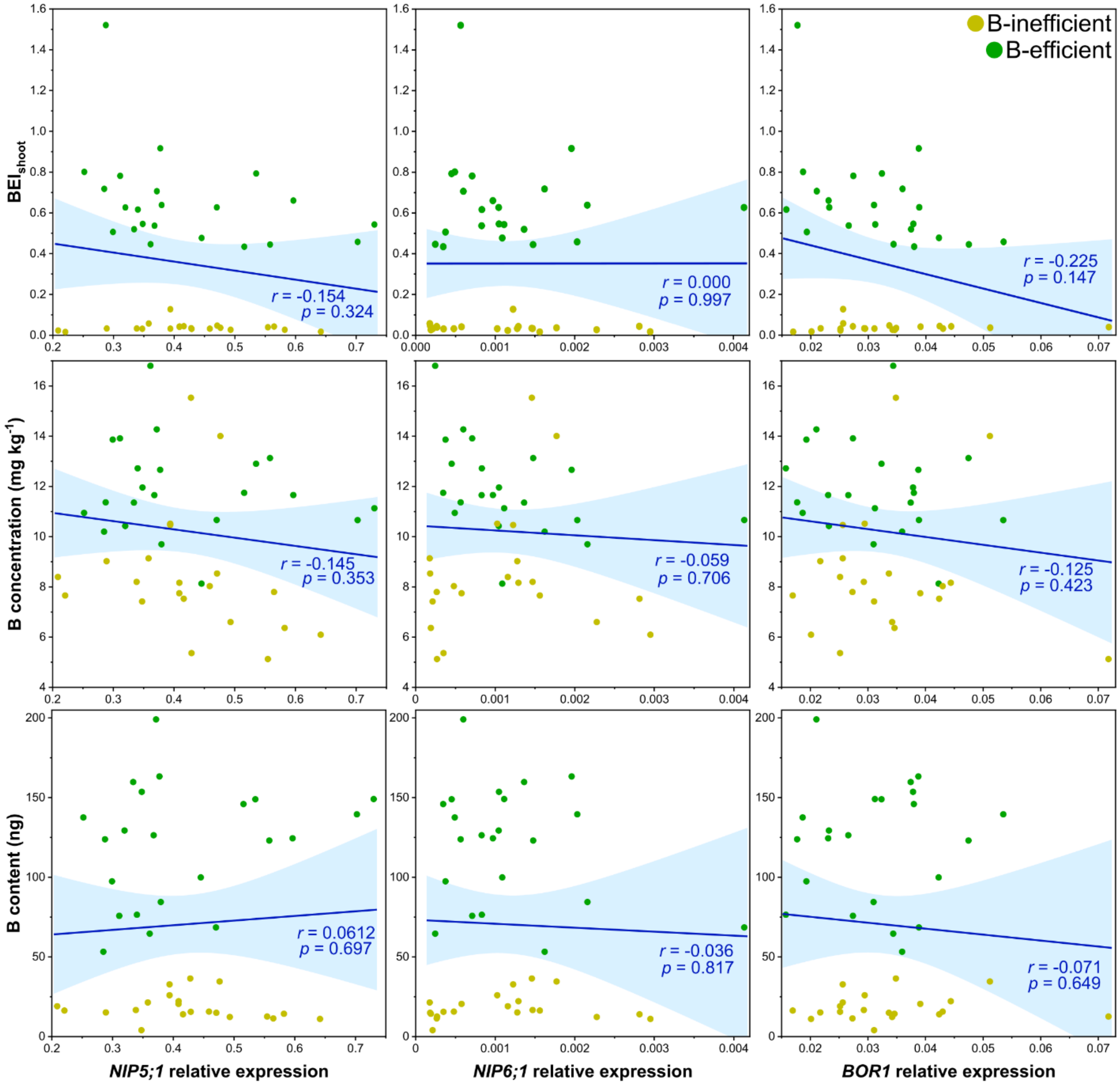

Fig. S11 Correlations of boron (B) transporter (*NIP5;1*, *NIP6;1* and *BOR1*) relative expression levels against B efficiency index of the shoot (BEI_{shoot}) and shoot B concentration and content of 43 Arabidopsis accessions considered B-efficient (22) or B-inefficient (21) and grown under B-deficient conditions. Blue lines represent linear fit regression curves with 95% confidence interval bands shown. Pearson correlation coefficients (*r*) shown in each panel.

(a)

Boron Transporter	Nucleotide Substitution	Amino acid Substitution	Percentage (1135 Accessions)	BEI_{shoot} 0.9 Percentile efficient	BEI_{shoot} 0.1 Percentile inefficient	BEI_{root} 0.9 Percentile efficient	BEI_{root} 0.1 Percentile inefficient
AtNIP5;1	A - G	M_{12} to V_{12}	0.44	-	-	-	-
	G – A	V_{299} to I_{299}	0.09	-	-	-	-
AtNIP6;1	G – A	G_{193} to D_{193}	28.90	Com-1<br>Yo-0	Hau-0<br>Kin-0<br>Ull2-3<br>Van-0	Boot-1<br>Pog-0<br>Yo-0	CIBC-17<br>Lm-2<br>Se-0<br>Van-0
	G – A	V_{269} to I_{269}	24.05	Eden-1<br>Zü-1	Hovdala-2<br>War	Eden-1<br>Edi-0<br>Hovdala-2	Rsch-4<br>Tamm-2<br>Tsu-0<br>Wil-2
AtBOR1	T – G	V_{69} to G_{69}	0.09	-	-	-	Tamm-2
	C – A	D_{399} to E_{399}	0.26	-	-	-	Wil-2
	C – A	L_{548} to I_{548}	37.27	Cal-0<br>Com-1<br>Cvi-0<br>Eden-1<br>Tha-1<br>Var2-1	Duk<br>Ga-0<br>Hau-0<br>Ull2-3<br>Van-0	Boot-1<br>Eden-1<br>Lom1-1<br>Me-0<br>Tha-1	Lis-1<br>Lm-2<br>Ms-0<br>Rsch-4<br>Se-0<br>Tsu-0<br>Van-0

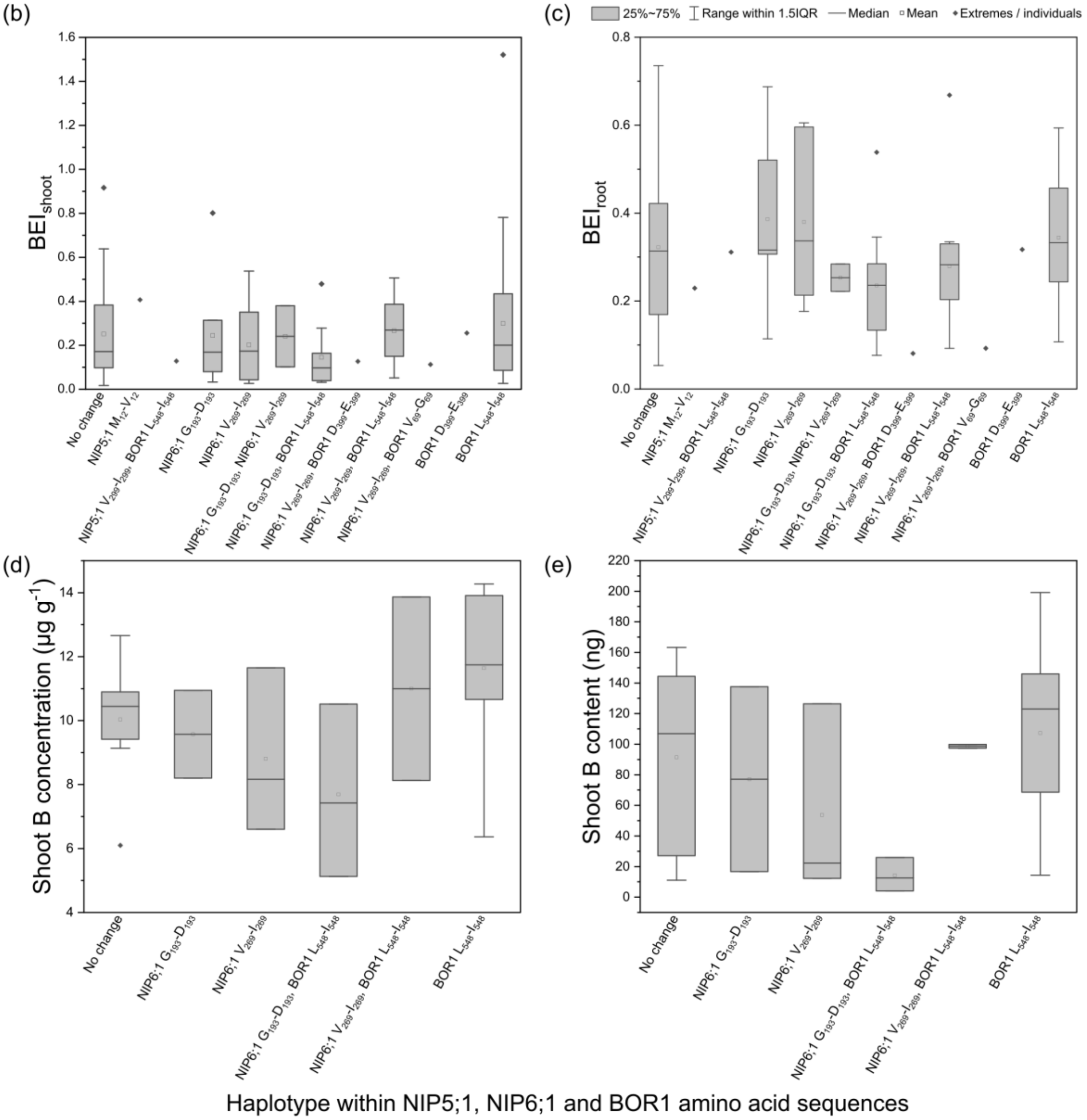

Fig. S12 (figure caption on next page)

Fig. S12 (previous page) Association between the amino acid sequence haplotypes of the Arabidopsis boron (B) transport proteins NIP5;1, NIP6;1 and BOR1 and B efficiency and B uptake. Included in this analysis are the 104 accessions that are included in both the 185 accession panel used in this study and the 1135 accessions with haplotype data in the SNP★ar tool. (a) Amino acid variants in accessions of the shoot and root B efficiency index score (BEI_{shoot} or BEI_{root}) 90th (B-efficient) or 10th (B-inefficient) percentiles. The NIP5;1 amino acid sequences of the accessions investigated were shared with that of Col-0 with the exception of Petergof (V_{299} to I_{299}) and Zdr-1 (M_{12} to V_{12}). The frequencies of these proteoforms indicated that these protein variants are rare. Moreover, they were not associated with B-efficiency. The NIP6;1 variants G_{193} to D_{193} and V_{269} to I_{269} also exist among the accessions of the current study. However, these variants occur in accessions with both high and low shoot and root Boron Efficiency Index scores. The variants V_{69} to G_{69} (one accession; Tamm-2) and D_{399} to E_{399} (two accessions; Stw-0, Wil-2) exist within BOR1 within accessions with a low BEI_{root}, but at frequencies too low to confirm a role in B efficiency. In addition, 49 of the investigated accessions carry a L_{548} to I_{548} exchange in BOR1, which is present both in B-efficient and B-inefficient accessions. (b-e) Variation in BEI_{shoot} (b), BEI_{root} (c), shoot B concentration (d) and shoot B content (e) in within amino acid haplotypes of NIP5;1, NIP6;1 and BOR1. Diamond shapes represent extreme accessions outside of 1.5 times the interquartile range where at least four data-points are available for each haplotype, or individual values where data for only a single accession exist for a given haplotype. None of trait scores were significantly different at $p < 0.05$ between the different haplotypes for any of the investigated proteins.

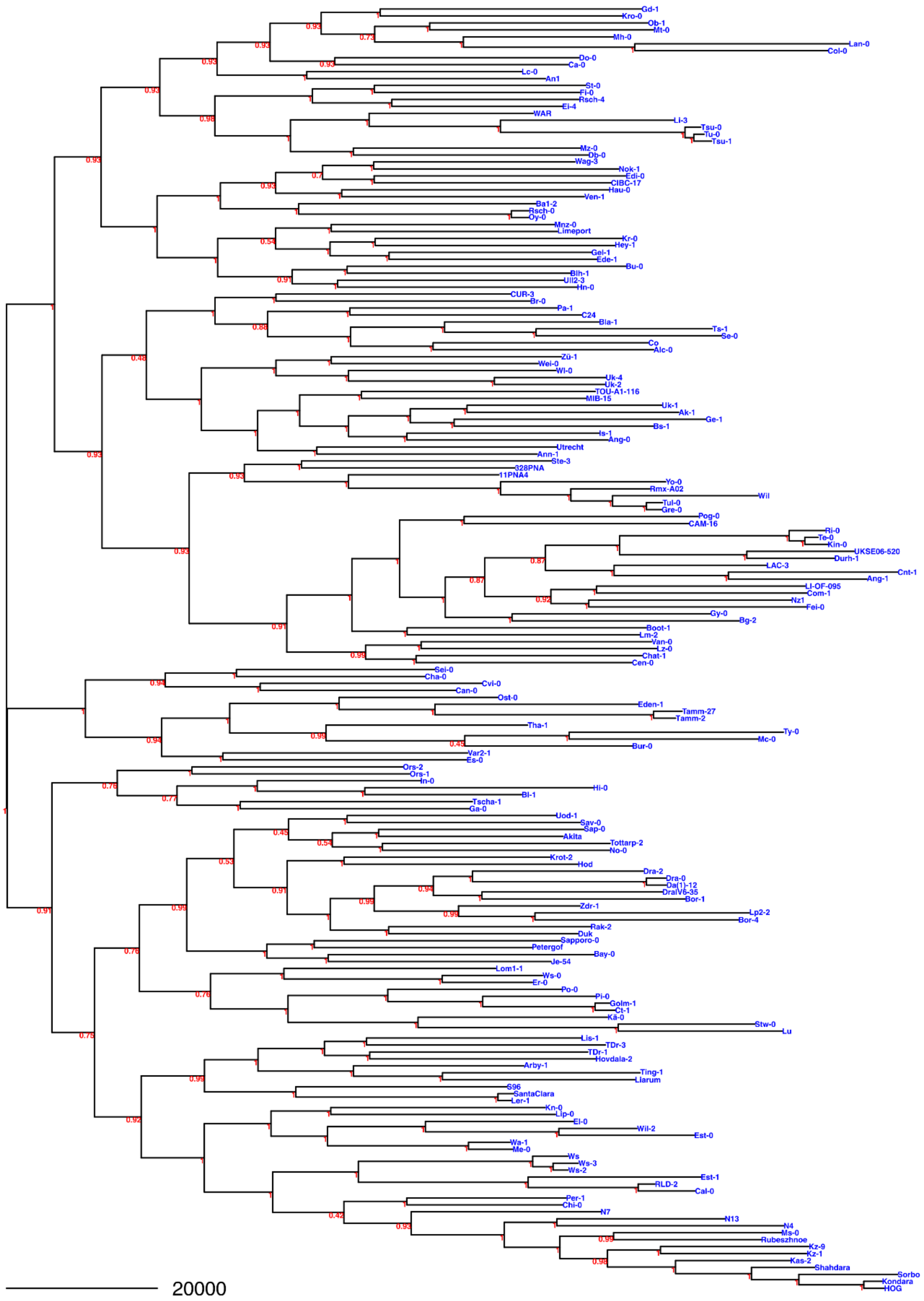

Fig. S13 Dendrogram showing the phylogenetic relationship of the 185 Arabidopsis accessions.

Accession names are displayed in blue. Numbers in red at junctions refer to bootstrap scores.

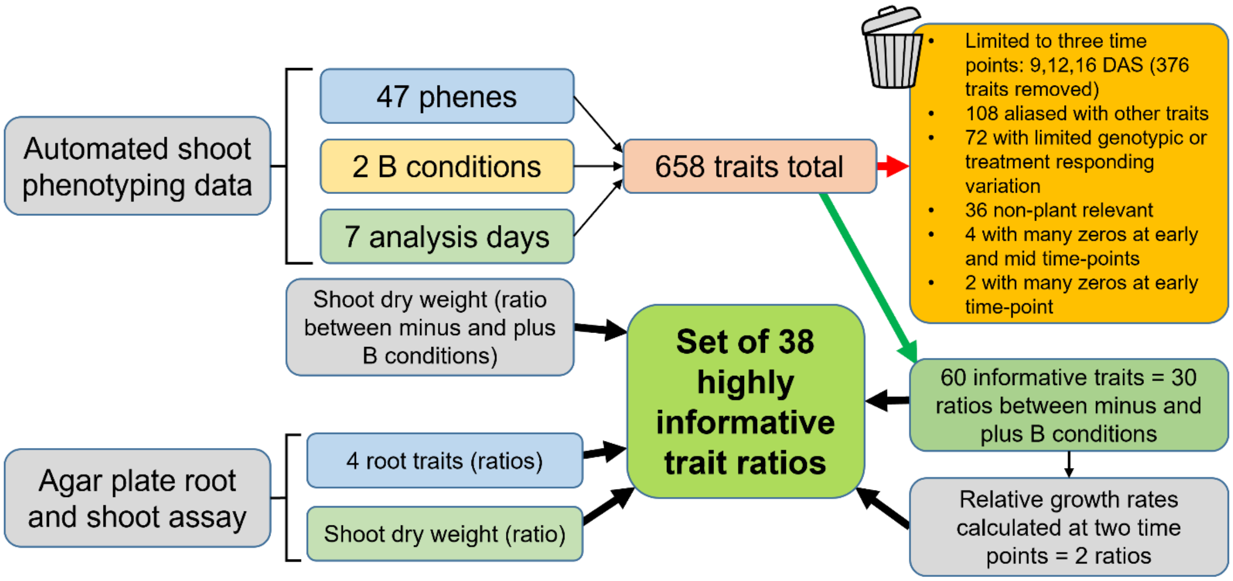

Fig. S14 Pipeline to reduce Arabidopsis traits measured across experiments to 38 informative traits for *k*-means analysis. DAS refers to days after sowing. B refers to boron.

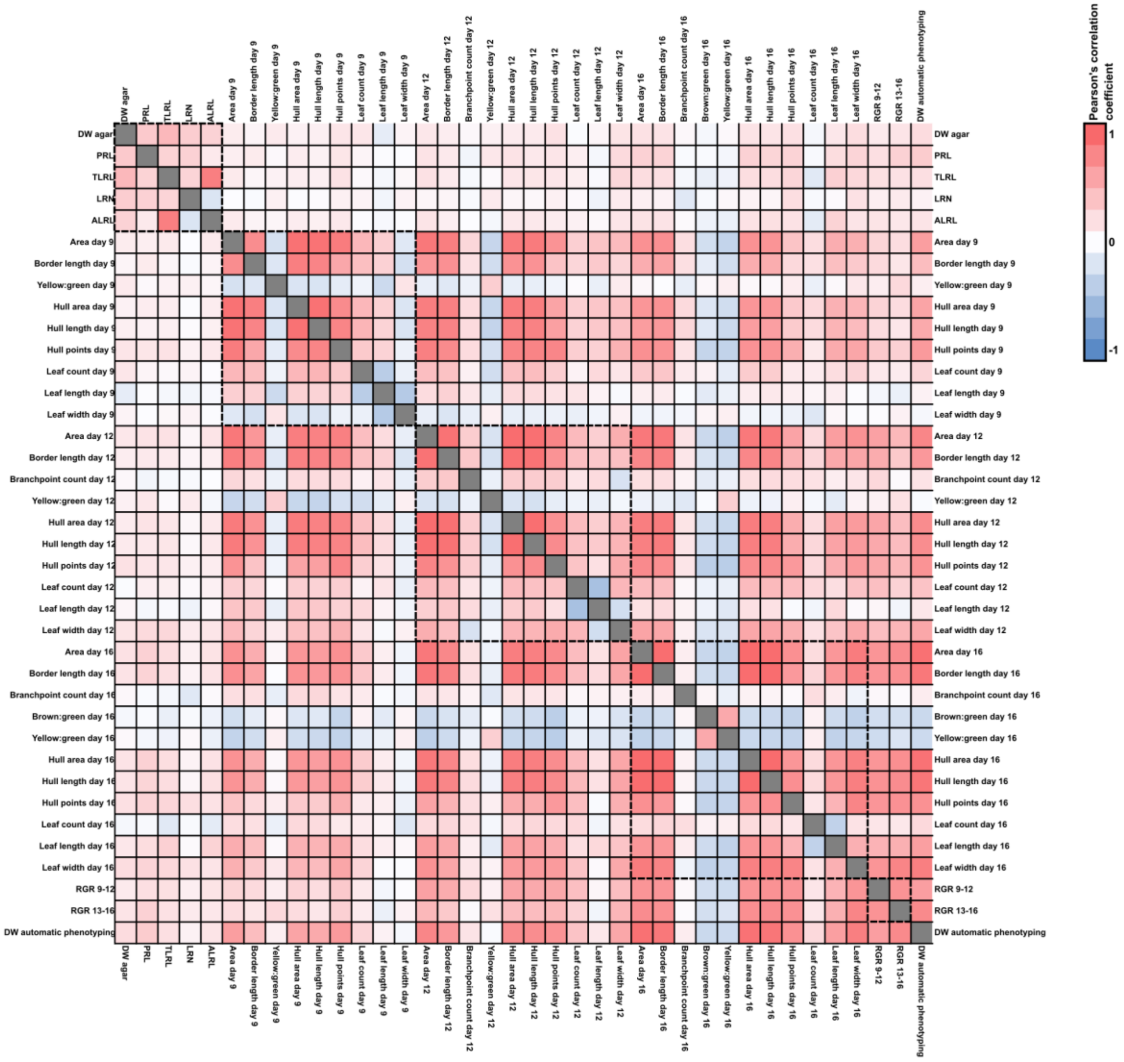

Fig. S15 Correlations table for all 38 Arabidopsis root and shoot traits included in *k*-means analysis. Cells are colour-coded by Pearson correlation coefficient, where deeper red colours represent a stronger positive correlation, and deeper blue colours represent a stronger negative correlation. Dotted line boxes are drawn around trait groups corresponding to root traits, automatic phenotyping by imaging day, and relative growth rate (RGR) data, to allow easier visualization of comparisons within trait groups. DW refers to shoot dry weight, PRL to primary root length, TLRL to total lateral root length, LRN to lateral root number, and ALRL to average lateral root length.

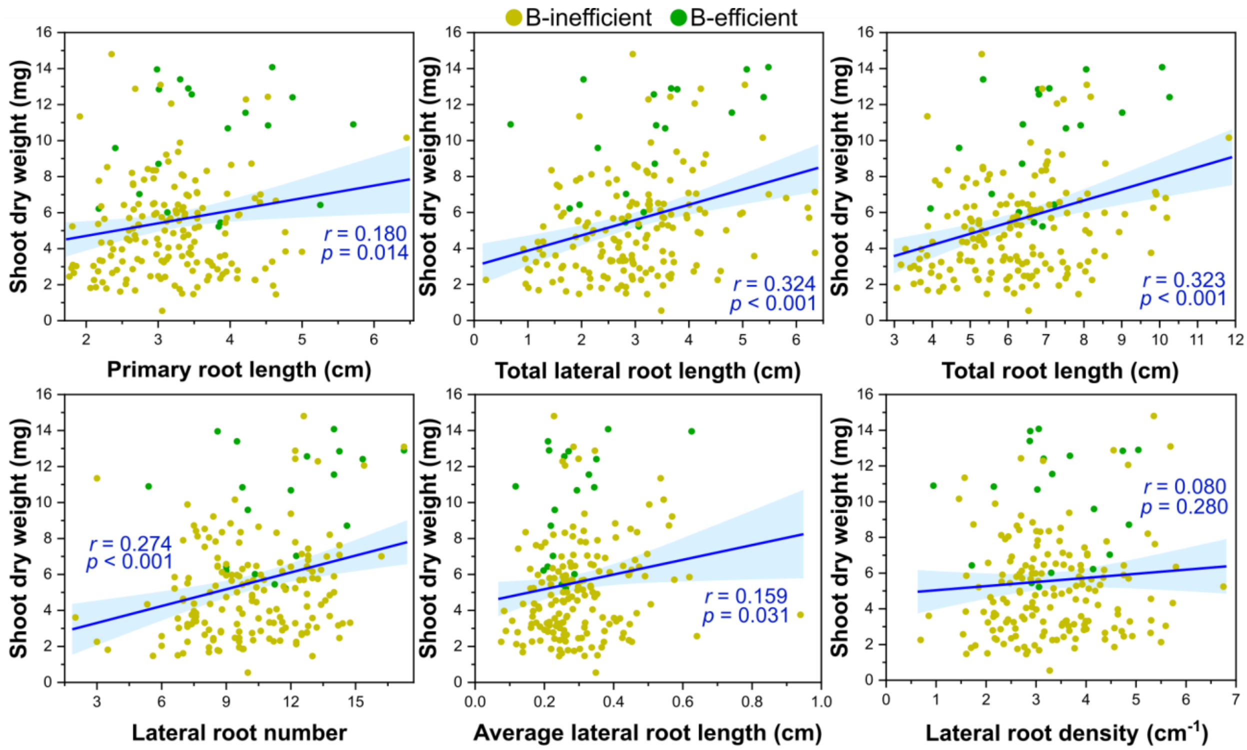

Fig. S16 Correlations between root architectural traits and shoot dry weight of 185 Arabidopsis accessions grown in boron (B)-deficient conditions. Blue lines represent linear fit regression curves with 95% confidence interval bands shown. Pearson correlation coefficients (*r*) shown in each panel.

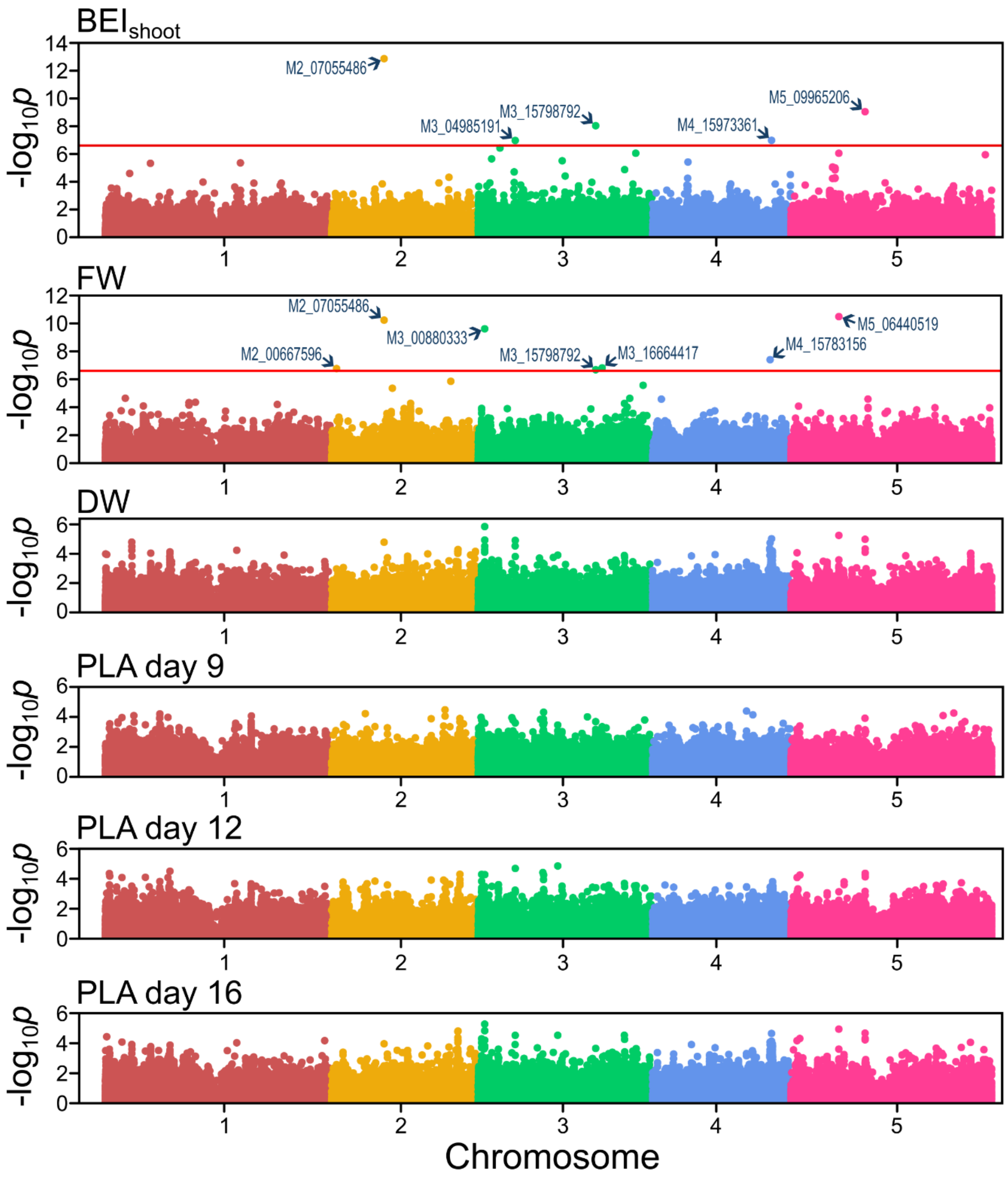

Fig. S17 Genome-wide SNP associations with boron (B)-efficiency index of the Arabidopsis shoot (BEI_{shoot}) and ratios of trait performance under B-deficient compared to B-sufficient conditions of shoot fresh (FW) and dry (DW) weight, projected leaf area (PLA) at day 9, PLA at day 12 and PLA at day 16. Where present, horizontal red lines represent the Bonferroni corrected significance threshold at $p < 0.05$. Marker names are listed where marker-trait associations exceeded this threshold, as indicated by blue arrows.

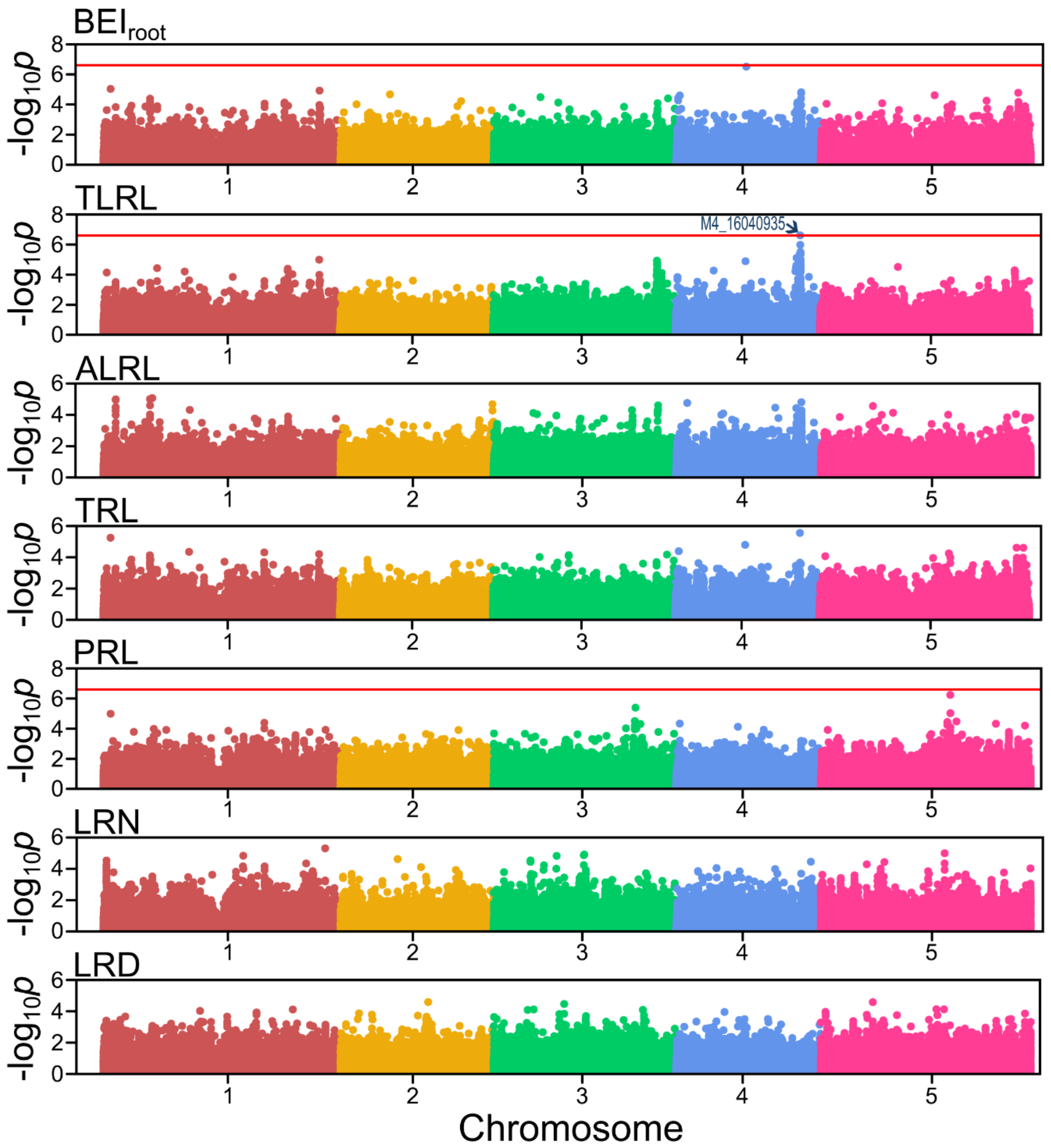

Fig. S18 Genome-wide SNP associations with boron (B)-efficiency index of the Arabidopsis root (BEI_{root}) and ratios of trait performance under B-deficient compared to B-sufficient conditions of total lateral root length (TLRL), average lateral root length (ALRL), total root length (TRL) primary root length (PRL), lateral root number (LRN) and lateral root density along the primary root (LRD). Where present, horizontal red lines represent the Bonferroni corrected significance threshold at $p < 0.05$. Marker names are listed where marker-trait associations exceeded this threshold, as indicated by blue arrows.

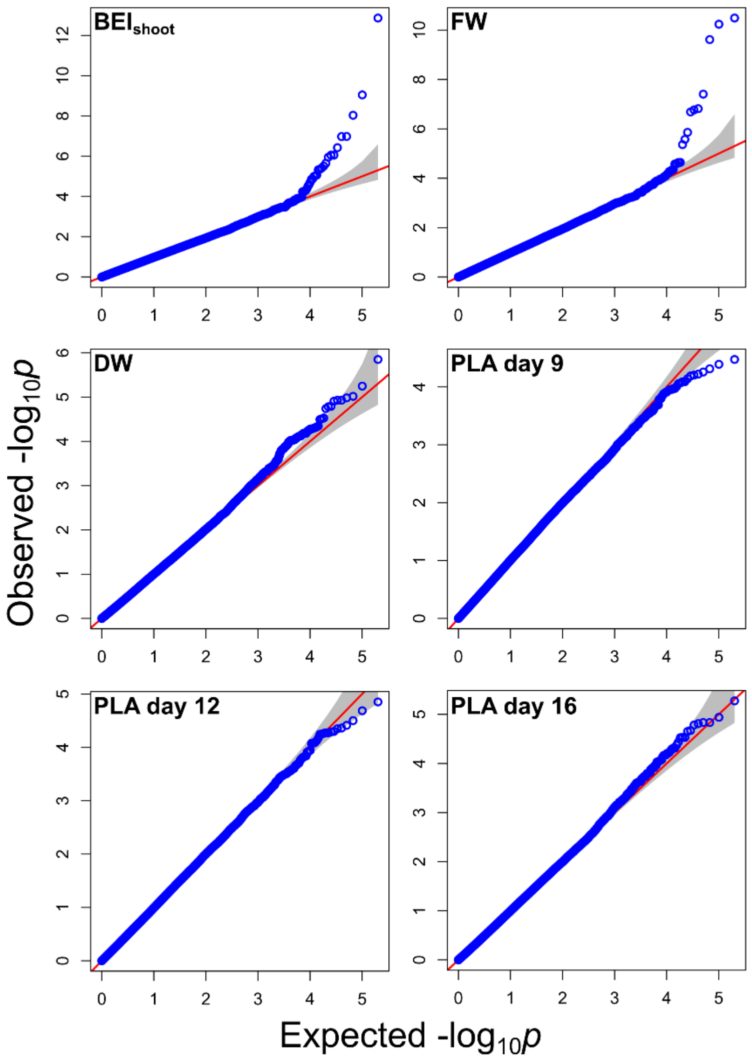

Fig. S19 Quantile-quantile (QQ) plots comparing expected with observed -$\log_{10}p$ values obtained from genome-wide association analysis of boron (B)-efficiency index of the Arabidopsis shoot (BEI_{shoot}) and ratios of trait performance under B-deficient compared to B-sufficient conditions of shoot fresh (FW) and dry weight (DW) and projected leaf area (PLA) at days 9, 12 and 16.

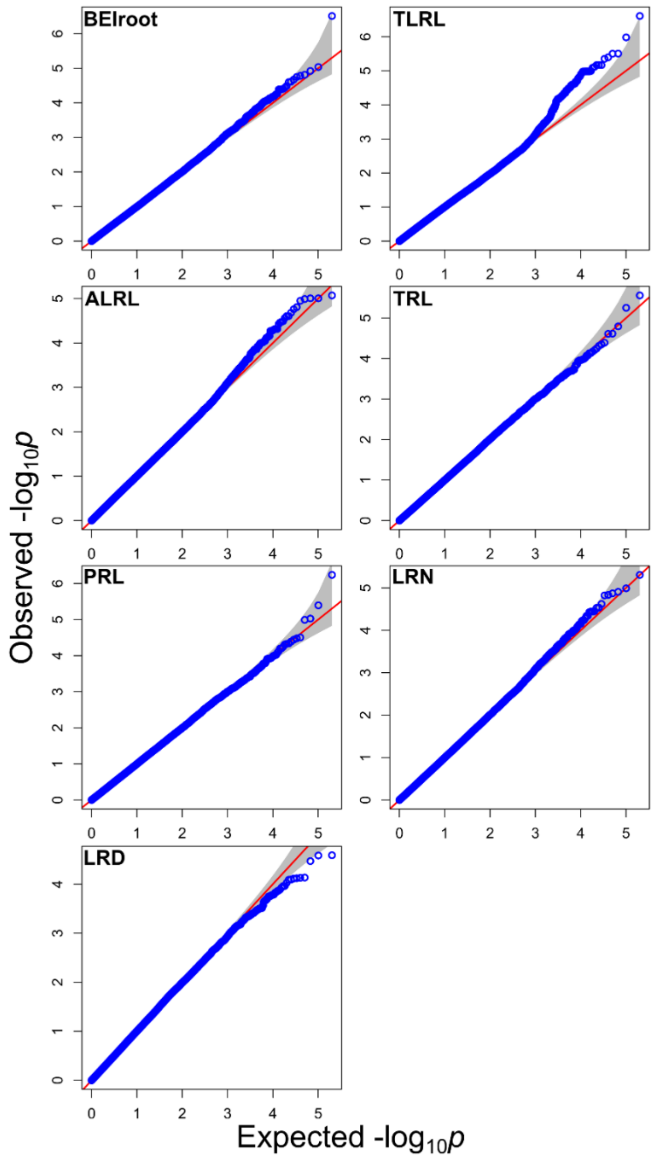

Fig. S20 Quantile-quantile (QQ) plots comparing expected with observed $-\log_{10}P$ values obtained from genome-wide association analysis of boron (B)-efficiency index of the Arabidopsis root (BEI_{root}) and ratios of trait performance under B-deficient compared to B-sufficient conditions of total lateral root length (TLRL), average lateral root length (ALRL), total root length (TRL), primary root length (PRL), lateral root number (LRN) and lateral root density (LRD).

(a)

Gene	Haplotype	Number of accessions (of 104)	BEI_{shoot} 90th Percentile efficient	BEI_{shoot} 10th Percentile inefficient	BEI_{root} 90th Percentile efficient	BEI_{root} 10th Percentile inefficient	Amino acid substitutions	Percentage (1135 Accessions)
BOR7	a	75	Ca-0<br>Yo-0<br>Zü-1<br>Var2-1	Duk<br>Hovdala-2<br>Ga-0<br>Kin-0<br>Ull2-3<br>Van-0<br>WAR	Hovdala-2<br>Lom1-1<br>Uod-1<br>Boot-1<br>Edi-0<br>Pog-0<br>Yo-0<br>Gy-0	CIBC-17<br>Ms-0<br>TAMM-2<br>Lm-2<br>Rsch-4<br>Tsu-0<br>Van-0<br>Hod<br>Lis-1<br>Rak-2	-	-
	b	3	-	-	-	-	L_{27} to F_{27}<br>Q_{583} to P_{583}	2.20%<br>39.56%
	c	2	-	-	-	Wil-2	L_{27} to F_{27}	2.20%
	d	1	-	Gel-1	-	Gel-1	G_{160} to E_{160}<br>I_{553} to T_{553}<br>Q_{583} to P_{583}<br>N_{609} to K_{609}<br>A_{639} to P_{639}	0.35%<br>0.79%<br>39.56%<br>0.88%<br>0.88%
	e	1	-	Hau-1	-	-	M_{388} to I_{388}	2.64%
	f	1	-	-	-	-	V_{404} to M_{404}<br>Q_{583} to P_{583}	0.09%<br>39.56%
	g	15	Cal-0<br>Com-1<br>Es-0<br>Tha-1	-	Gre-0<br>Tha-1	Se-0	Q_{583} to P_{583}	0.3956
	h	1	Cvi-0	-	-	-	Y_{637} to S_{637}	0.09%
	i	1	Sorbo	-	-	-	N_{328} to T_{328}<br>Q_{583} to P_{583}	0.09%<br>39.56%
	j	4	Eden-1<br>Hn-0	-	Eden-1<br>Hn-0<br>Me-0<br>Ost-0	-	I_{235} to T_{235}<br>L_{244} to V_{244}<br>Q_{583} to P_{583}<br>S_{610} to P_{610}<br>L_{642} to M_{642}	5.11%<br>5.11%<br>39.56%<br>5.02%<br>5.02%

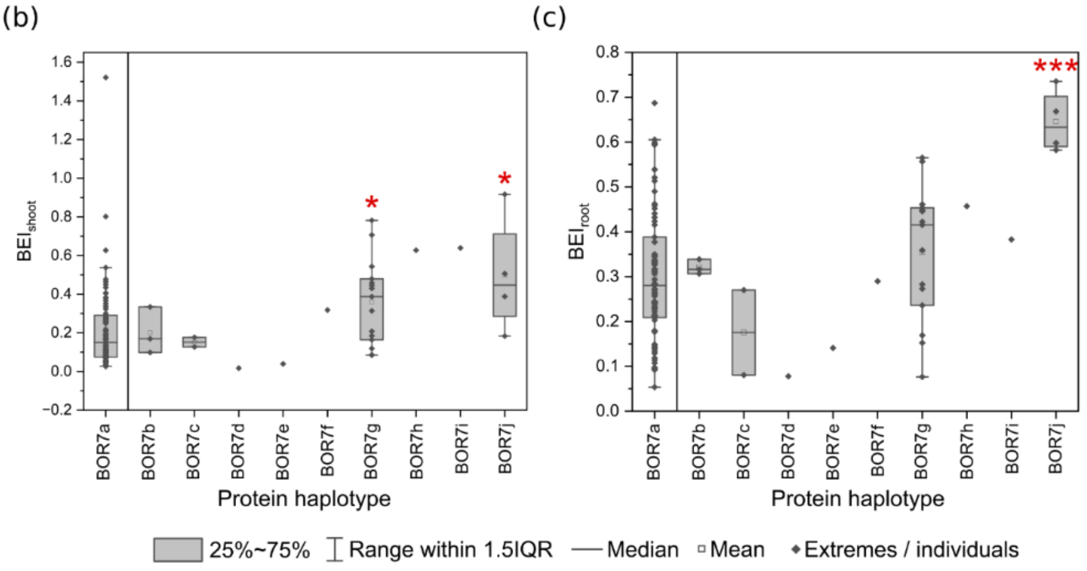

Fig. S21 (figure caption on next page)

Fig. S21 (previous page) Association between the amino acid sequence haplotypes of the Arabidopsis boron (B) transport protein BOR7 (*AT4G32510*) and B efficiency. Included in this analysis are the 104 accessions that are included in both the 185 accession panel used in this study and the 1135 accessions with haplotype data in the SNP★ar tool. (a) Protein haplotypes for BOR7 with the number of accessions exhibiting each haplotype (of 104 accessions) shown, along with lists of accessions of the shoot and root B efficiency index score (BEI_{shoot} or BEI_{root}) 90th (B-efficient) or 10th (B-inefficient) percentiles that exhibit each haplotype. Amino acid substitutions associated with each haplotype are listed, along with the incidence of each substitution across the entire 1135 accession panel included in the SNP★ar tool. (b,c) Variation in BEI_{shoot} (b) or BEI_{root} (c) between different protein haplotypes of BOR7. Stars represent significantly different haplotype BEI_{shoot} or BEI_{root} scores compared to the reference haplotype (BOR7a), where * represents $p < 0.05$.and *** represents $p < 0.001$.

(a)

Gene	Haplotype	Number of accessions (of 104)	BEI_{shoot} 90th Percentile efficient	BEI_{shoot} 10th Percentile inefficient	BEI_{root} 90th Percentile efficient	BEI_{root} 10th Percentile inefficient	Amino acid substitutions	Percentage (1135 Accessions)
PME44	a	9	Ca-0	-	-	-	-	-
	b	4	-	-	-	Ms-0	L_{249} to F_{249}<br>F_{331} to Y_{331}	0.88%<br>92.42%
	c	8	Eden-1<br>Cal-0<br>Hn-0<br>Yo-0	-	Eden-1<br>Hn-0<br>Yo-0	-	F_{331} to Y_{331}<br>Y_{524} to N_{524}	92.42%<br>17.53%
	d	73	Sorbo<br>Com-1<br>Es-0<br>Tha-1<br>Zü-1<br>Var2-1	Hovdala-2<br>Ga-0<br>Kin-0<br>Ull2-3<br>Gel-1<br>Hau-0<br>Van-0<br>WAR	Hovdala-2<br>Lom1-1<br>Uod-1<br>Boot-1<br>Edi-0<br>Gre-0<br>Me-0<br>Tha-1<br>Gy-0<br>Ost-0	CIBC-17<br>Se-0<br>TAMM-2<br>Gel-1<br>Rsch-4<br>Tsu-0<br>Van-0<br>Wil-2<br>Hod<br>Lis-1<br>Rak-2	F_{331} to Y_{331}	92.42%
	e	1	-	Duk	-	-	F_{331} to Y_{331}<br>I_{363} to L_{363}	92.42%<br>0.09%
	f	1	Cvi-0	-	-	-	F_{331} to Y_{331}<br>I_{356} to V_{356}	92.42%<br>0.09%
	g	7	-	-	Pog-0	Lm-2	G_{167} to V_{167}<br>F_{331} to Y_{331}<br>Y_{524} to N_{524}	7.67%<br>92.42%<br>17.53%
	h	1	-	-	-	-	G_{167} to V_{167}<br>F_{331} to Y_{331}	7.67%<br>92.42%

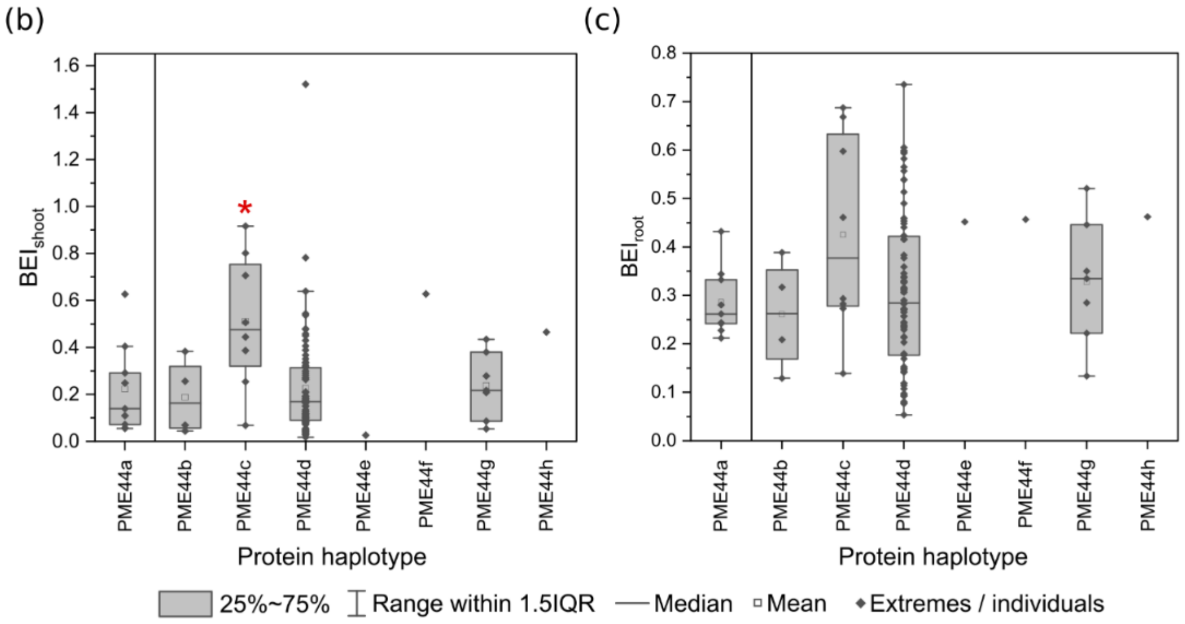

Fig. S22 (figure caption on next page)

Fig. S22 (previous page) Association between the amino acid sequence haplotypes of the Arabidopsis protein PME44 (*AT4G33220*) and boron (B) efficiency. Included in this analysis are the 104 accessions that are included in both the 185 accession panel used in this study and the 1135 accessions with haplotype data in the SNP★ar tool. (a) Protein haplotypes for PME44 with the number of accessions exhibiting each haplotype (of 104 accessions) shown, along with lists of accessions of the shoot and root B efficiency index score (BEI_{shoot} or BEI_{root}) 90th (B-efficient) or 10th (B-inefficient) percentiles that exhibit each haplotype. Amino acid substitutions associated with each haplotype are listed, along with the incidence of each substitution across the entire 1135 accession panel included in the SNP★ar tool. (b,c) Variation in BEI_{shoot} (b) or BEI_{root} (c) between different protein haplotypes of PME44. Stars represent significantly different haplotype BEI_{shoot} or BEI_{root} scores compared to the reference haplotype (PME44a), where * represents p < 0.05.and *** represents p < 0.001.

(a)

Gene	Haplotype	Number of accessions (of 104)	BEI_{shoot} 90th Percentile efficient	BEI_{shoot} 10th Percentile inefficient	BEI_{root} 90th Percentile efficient	BEI_{root} 10th Percentile inefficient	Amino acid substitutions	Percentage (1135 Accessions)
LIME1	a	26	Ca-0<br>Zü-1	Kin-0<br>Van-0	Edi-0<br>Gy-0	Van-0<br>Lis-1<br>Rak-2	-	-
	b	1	-	-	-	-	M_{22} to K_{22}<br>S_{55} to F_{55}	21.59%<br>0.44%
	c	7	-	-	Pog-0	Lm-2<br>Wil-2	M_{22} to K_{22}<br>K_{308} to E_{308}	21.59%<br>65.55%
	d	1	Cvi-0	-	-	-	M_{22} to K_{22}<br>I_{181} to V_{181}<br>K_{308} to E_{308}	21.59%<br>0.53%<br>65.55%
	e	10	Eden-1<br>Cal-0<br>Hn-0<br>Tha-1<br>Yo-0<br>Var2-1	-	Eden-1<br>Hn-0<br>Tha-1<br>Yo-0	-	M_{22} to K_{22}<br>I_{28} to L_{28}<br>K_{308} to E_{308}	21.58%<br>10.57%<br>65.55%
	f	9	-	Hovdala-2<br>Ga-0	Hovdala-2	-	R_{94} to K_{94}	3.52%
	g	2	Sorbo	-	-	-	K_{308} to E_{308}<br>G_{338} to A_{338}	65.55%<br>1.41%
	h	40	Com-1<br>Es-0	Duk<br>Ull2-3<br>Gel-1<br>Hau-0<br>WAR	Uod-1<br>Boot-1<br>Gre-0<br>Me-0	CIBC-17<br>Ms-0<br>Se-0<br>TAMM-2<br>Gel-1<br>Rsch-4<br>Tsu-0<br>Hod	K_{308} to E_{308}	65.55%
	i	3	-	-	Lom1-1	-	A_{240} to T_{240}	1.41%
	j	2	-	-	-	-	E_{192} to Q_{192}<br>K_{308} to E_{308}	0.18%<br>65.55%
	k	1	-	-	-	-	C_{138} to S_{138}<br>S_{276} to A_{276}	2.38%<br>1.76%
	l	1	-	-	Ost-0	-	I_{28} to L_{28}<br>K_{308} to E_{308}	10.57%<br>65.55%
	m	1	-	-	-	-	D_6 to G_6<br>K_{308} to E_{308}	0.09%<br>65.55%

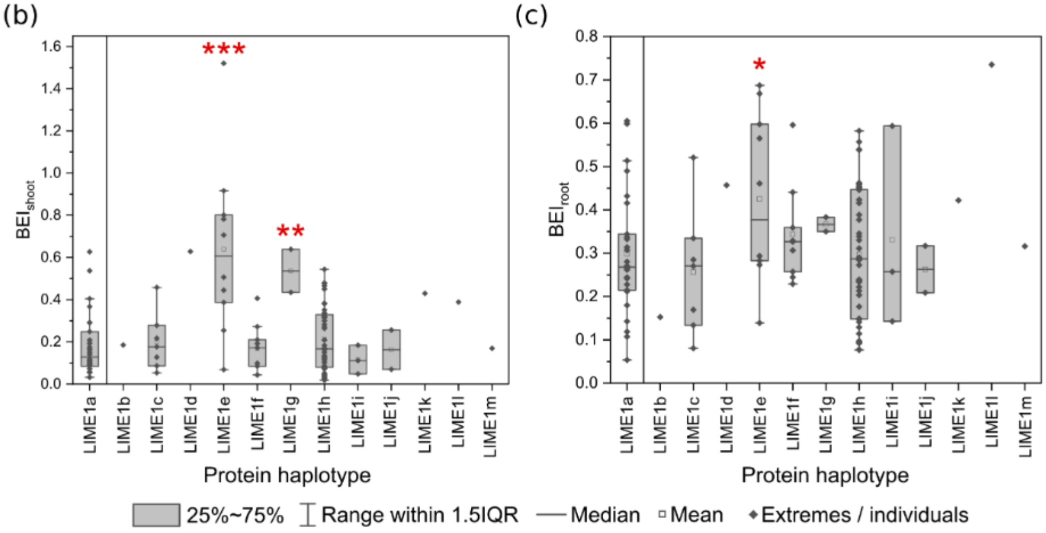

Fig. S23 (figure caption on next page)

Fig. S23 (previous page) Association between the amino acid sequence haplotypes of the Arabidopsis protein LIME1 (*AT4G33110*) and boron (B) efficiency. Included in this analysis are the 104 accessions that are included in both the 185 accession panel used in this study and the 1135 accessions with haplotype data in the SNP★ar tool. (a) Protein haplotypes for LIME1 with the number of accessions exhibiting each haplotype (of 104 accessions) shown, along with lists of accessions of the shoot and root B efficiency index score (BEI_{shoot} or BEI_{root}) 90th (B-efficient) or 10th (B-inefficient) percentiles that exhibit each haplotype. Amino acid substitutions associated with each haplotype are listed, along with the incidence of each substitution across the entire 1135 accession panel included in the SNP★ar tool. (b,c) Variation in BEI_{shoot} (b) or BEI_{root} (c) between different protein haplotypes of LIME1. Stars represent significantly different haplotype BEI_{shoot} or BEI_{root} scores compared to the reference haplotype (LIME1a), where * represents $p < 0.05$.and *** represents $p < 0.001$.

Table S5 Primer sequences for RT-qPCR amplification of boron transporter encoding genes and the used reference gene (AtEF1a).

**Primer**	**Sequence**
AtNIP5;1 forward	CACCGATTTTCCCTCTCCTGAT
AtNIP5;1 reverse	GCATGCAGCGTTACCGATTA
AtNIP6;1 forward	GGCAATGGTTACAGCCGGAT
AtNIP6;1 reverse	GGAGCTGAGACGCTTATTGGTT
AtBOR1 forward	AATCTCGCAGCGGAAACG
AtBOR1 reverse	TGGAGTCGAACTTGAACTTGTC
AtEF1a forward	CCTTGGTGTCAAGCAGATGA
AtEF1a reverse	TGAAGACACCTCCTTGATGATTT

Methods S1 Detailed methods used in both soil-substrate and in vitro cultivation systems, and downstream analysis and processing stages.

Plant material and growth

A total of 185 *Arabidopsis thaliana* (L.) Heynh. accessions were selected based on their expected genotypic variation and on their diverse origin representing sites expected to vary greatly in soil B availability (Table **S1**). *Atnip5;1* knock-down mutant (SALK_122287; NASC: N622287) seeds were obtained from The European Arabidopsis Stock Centre (Loughborough, United Kingdom) and used as a control for B-deficiency treatments.

Plant cultivation on soil-substrate in an automated plant phenotyping system

The automated phenotyping experiment took place from 23/03/2016 to 13/04/2016 at the IPK Gatersleben (51° 49' 28.9" N, 11° 16' 44.9" E). This was performed using an automated plant transport and imaging system (IPK automated plant phenotyping system for small plants) situated in a controlled environment plant growth chamber (Junker et al., 2015). Measured parameters are described in Table **S3**. Growth chamber conditions were set to long-day conditions (16 h day/8 h night) at 20/18 °C, 60/75 % relative humidity, and ~180 µmol light intensity light intensity. A total of 384 plant carriers were used, each consisting of 12 growth units of 4x4 cm (length x width) arranged in a 3x4 pattern (Fig. **1**). Carriers were arranged in 48 blocks of eight carriers situated across 12 conveyor belt lanes (six on each side of the plant growth chamber; Table **S4**). The 185 selected *A. thaliana* accessions were represented across twelve biological replicates per accession and treatment (B-deficient; "-B" and B-sufficient; "+B"). Accessions were each distributed across six carriers, three of which were subjected to -B (< 0.1 mg-B kg-substrate^{-1}) conditions, and three of which were subjected to +B conditions (~2.4 mg B / kg substrate). On each carrier of 12 growth units, four plants of each of three accessions were cultivated. Replicate carriers were distributed throughout the system using a randomised complete block design with three replicates (Table **S4**).

Growth substrate used was a B-free (< 0.1 mg-B kg-substrate^{-1}) white-peat volcanic clay mixture (from here named zerosoil-substrate) with a dry matter of ca. 30 %. Zerosoil-substrate was supplemented with 0.5% $CaCO_3$ and 0.3% CaO (w/w) by mixing 20 kg batches of soil with 1 L of $CaCO_3$ (100 g L^{-1}) and 1 L of CaO (60 g L^{-1}) in a clean cement mixer (Lescha Typ SBM P 150L) for 10 min. Thereafter, zerosoil-substrate was manually mixed every second day to allow even drying of the substrate. Around 1-2 weeks after preparation, clumps were sieved out. The following 100x micro- and 100x macronutrient stock solutions were prepared: NH_4NO_3 60 g L^{-1}, KH_2PO_4 40 g L^{-1}, K_2SO_4 6 g L^{-1}, $MgSO_4$ 20 g L^{-1}, $CuSO_4$ 1.3 g L^{-1}, $ZnSO_4$ 1.3 g L^{-1}, $MnCl_2$ 4 g L^{-1}, $(NH_4)_6Mo_7O_{24}$ x 4 H_2O 8.5 mg L^{-1}, NaFeEDTA 0.7 g L^{-1} and H_3BO_3 1.4 g L^{-1}. From these stocks, an 8x-concentrated nutrient solution was prepared for all nutrients excluding B and 250 mL were mixed per 1 kg sieved zerosoil-substrate in a cement mixer with a pressure sprayer. No H_3BO_3 stock was applied to zerosoil-substrate used for the B-deficient treatment. For the B-sufficient treatment, H_3BO_3 was added to reach a final soil concentration of 2.5 mg B kg-soil^{-1}. This soil concentration was confirmed by a soil analysis performed by AGROLAB Boden- und Pflanzenbewertungsdienst GmbH using method VDLUFA 1, A13.1.1, which measured an average soil B concentration of 2.43 mg kg^{-1} at experiment start. At the same time, the B-deficient soil was determined to contain B concentrations lower than the detection limit of 0.1 mg kg^{-1}. Each 4x4 cm growth unit was filled with ca. 17 g processed zerosoil-substrate.

Seeds of the 185 *A. thaliana* accessions were spread out on wet filter paper and left to pre-germinate in the dark at 20°C overnight, and then were sown out onto the zerosoil-substrate in each growth unit. In order to initiate germination, carriers underwent a stratification period of 3 days at 5°C in the dark (75% relative humidity) following seed sowing. After this, carriers were transferred to the plant growth chamber. Conditions were set to long-days (16h day/8h night) at 16/14°C, 75% relative humidity, for two days at 120 µmol light intensity and for two days at 180 µmol light intensity. After this, the temperature was increased to 20/18°C, with all other conditions kept the same. Plants were watered every day using an automated weighing and watering system to readjust to ca. 70% field capacity using ultrapure Milli-Q water (Merck, Milli-

Q IQ7000) pre-treated with the B-chelator Amberlite IRA-743 (3 g L^{-1}; Sigma) to prevent any B contamination.

Between 5 and 16 days after sowing (DAS), but excluding 11 DAS, top view images of all individual plants were taken daily in the visible range of the light spectrum (VIS), and of static fluorescence signals (FLUOR) as described previously in Junker et al. (2015). At the end of the experiment (20 DAS), shoot fresh weights (FW) of three pools of three representative plants per accession were measured by cutting the shoot directly above the substrate level and weighing. Dry weights were additionally quantified for each pool after drying the material at 60°C for 5 days. In each case, the average FW or DW per plant was calculated by dividing the weight of the pools by the number of plants included in each pool. The relative shoot B efficiency indices (BEI_{shoot}) of the 185 *A. thaliana* accessions were calculated as per equation [1]:

$$[1] \qquad BEI_{shoot} = \frac{FW_{B0}}{FW_{B1}} \times \frac{PLA_{B0}}{PLA_{B1}}$$

where FW_{B0} is the mean FW under -B conditions, FW_{B1} is the mean FW under +B conditions, PLA_{B0} is the mean projected leaf area in -B conditions based on fluorescence imaging ($pixels^2$), and PLA_{B1} is the mean projected leaf area in +B conditions.

Relative growth rates (RGR) were calculated for individual plants on both a daily basis and in three day intervals between 9 and 12 DAS, and 13 and 16 DAS, as per equation [2]:

$$[2] \qquad RGR = \frac{ln\,(S_2) - ln\,(S_1)}{t_2 - t_1}$$

where ln is the natural logarithm, S_1 is the size at time one, S_2 is the size at time two, t_1 is time point one and t_2 is time point 2.

Photosynthetic operating efficiency of all plants was measured 16 DAS using the FluorCam device (Photon Systems Instruments, Brno, Czech Republic) installed in the automated plant phenotyping system for small plants at a photosynthetically active radiation of 590 µmol m^{-2} s^{-1} during measurements as described in Tschiersch et al. (2017).

Image and data processing

The Integrated Analysis Platform (IAP) was used for processing and automated feature extraction of images taken by the automated plant phenotyping system (Klukas et al., 2014). The multi-sensor setups at IPK (VIS, FLUO, NIR, FluorCam) supported the assessment of phenes corresponding to plant architecture, plant colouration, and levels of fluorophores, as well as efficiency of photosystem II. A total of 47 automatic imaging phenes were captured in the main database (Table **S3**). Automated phenotyping data logged before day 9 were removed due to large amounts of missing data (plants were too small to be detected by the imaging system at earlier stages), and after day 16 because of overlapping rosettes. Across all 4,608 initial experimental units, 258 were removed completely from further analysis due to plants having not germinated or having died during the experiment, or due to the existence of more than one plant per growth unit. Data were removed from an additional 16 experimental units on specific measurement days due to poor imaging data on that day, for example due to plants not being visible. Individual trait outliers were determined separately for -B and +B datasets, and were defined as values lying above five standard deviations above the mean or below five standard deviations below the mean, for each trait measured on each day. Arithmetic means were calculated for each phene for each Arabidopsis accession in each B treatment on each measurement day.

Root phenotyping of *in vitro* cultivated plants

Square Petri dishes (120 x 120 x 17mm, vented; Greiner) were used as growth containers for the *A. thaliana in vitro* cultures. The experiment included the 185 selected accessions (Table **S1**) represented across five plants per Petri dish per accession and treatment (-B and +B). Seeds of each accession were surface sterilised and then cold stratified at 4°C for two days. They were then subjected to a pre-culture for five days in an autoclaved, modified half-strength Murashige and Skoog (½ MS) solidified medium comprising 625 µM KH_2PO_4, 750 µM $MgSO_4$, 1.5 mM $CaCl_2$, 9.4 mM KNO_3, 1 mM NH_4NO_3, 75 µM $FeNa_2EDTA$, 0.055 µM $CoCl_2$, 0.05 µM $CuSO_4$, 50 µM $MnSO_4$, 0.5 µM Na_2MoO_4, 15 µM $ZnSO_4$, 2.5 µM KI, 1 mM MES, 0.5% Sucrose, pH 5.5 (KOH), 1% Phytagel

(Sigma-Aldrich Co. LLC). No B was specifically added to pre-culture media but nor was it specifically excluded. After cold stratification/pre-culture, five seedlings per accession were carefully transferred with forceps to autoclaved, solidified main-culture media with the same composition as the pre-culture medium but additionally containing 0.2 µM boric acid (-B) or 100 µM boric acid (+B). All main-culture media used was treated overnight with the B-chelator Amberlite IRA-743 (3 g/L) prior to autoclaving to remove all traces of B from the media. All Amberlite was sieved out of the growth media before autoclaving. The above-described boric acid concentrations were supplied to the media after Amberlite treatment and autoclaving, using separately autoclaved boric acid stock solutions.

Petri dishes were wrapped around the vented edges with Micropore tape and placed vertically in a Percival Scientific CU chamber (CLF Climatics) with a photoperiod of 16 h light at 22°C (120 µmol m^{-2} sec^{-1}) and 8 h dark at 19°C. All Petri dishes were re-positioned every second day to avoid positioning effects in the growth chamber. After 10 days of growth on the main-culture media, Petri dishes were scanned using an Expression 11000XL scanner (Epson). Shoots were then carefully separated from roots with forceps. After 3 days of drying at 65°C, the dry weights of pools of 3-5 shoots per accession and treatment were determined. Root systems were combed to prevent any overlap between roots prior to scanning. Primary root length (PRL), total lateral root length (TLRL), average lateral root length (ALRL) and lateral root number (LRN) were determined by analyzing the scanned images using EZ-Rhizo software (Shahzad et al., 2018). The relative root B efficiency indices (BEI_{root}) of the 185 *A. thaliana* accessions were calculated as per equation [3]:

$$[3] \qquad BEI_{root} = \frac{PRL_{B0}}{PRL_{B1}} \times \frac{TLRL_{B0}}{TLRL_{B1}}$$

where PRL_{B0} is the mean primary root length under -B conditions, PRL_{B1} is the mean primary root length under +B conditions, $TLRL_{B0}$ is the mean total lateral root length in -B conditions, and $TLRL_{B1}$ is the mean total lateral root length in +B conditions. Twelve plants per accession and condition were used for the calculation of the mean root trait values.

Mineral elemental analysis

Shoot ionomes of plants grown on both B treatments in zerosoil-substrate in the automated plant phenotyping system were determined for three pools of three representative biological replicates of 43 selected *A. thaliana* accessions after harvest at 20 DAS. Accessions were selected based on their BEI_{shoot} scoring (equation [1]): 17 B-efficient accessions of the 90th percentile (Var2-1, Hn-0, Yo-0, Cha-0, Tha-1, Db-0, Cal-0, Ty-0, Sorbo, Cvi-0, Ca-0, CUR-3, TOU-A1-116, Es-0, Zü-1, N7, Eden-1), 5 B-intermediate efficient accessions with BEI_{shoot} above 0.4 (Kn-0, Kas-2, Liarum, S96, In-0), three B-inefficient accessions (Col-0, Limeport, Uod-1) and 18 highly B-inefficient accessions of the 10th percentile (Bay-0, Duk, Gel-1, Hau-0, Hovdala-2, Kin-0, Kz-1, Nz1, Pa-1, Ri-0, Rld-2, Rmx-A02, Tsu-1, Tu-0, Ull2-3, Van-0, WAR, Ws). Plant material was sampled and dried at 65°C for one week. Approximately 5-10 mg of plant dry matter was digested in nitric acid (HNO_3) using a high-performance microwave reactor (UltraClave IV; MLS GmbH, Leutkirch, Germany). Elemental analysis was performed with a sector field high-resolution ICP-MS (Element 2, Thermo Fisher Scientific, Waltham, MA, USA) using software v.3.1.2.242 (Eggert and von Wirén, 2013). The elements B, Na, Mg, P, S, K, Ca, Mn, Fe, Cu and Zn were quantified.

RNA extraction, cDNA synthesis, and real-time quantitative PCR

Approximately 10-40 mg *A. thaliana* plant material from the accessions as selected above was harvested from 15-day old plant roots, immediately frozen in liquid nitrogen and ground with a pestle and mortar in liquid nitrogen. Total RNA was extracted using NucleoSpin RNA Plant Kits and DNase I treatment according to the manufacturer's instructions (Macherey-Nagel, Germany). Complementary DNA (cDNA) was synthesised from 1000 ng total RNA per sample using MuLV-Reverse Transcriptase (Fermentas, Germany) in a total volume of 20 µl and diluted to 1:20 with nuclease free water. RT-qPCR was performed in a 384 well thermocycler (CFX384 TouchTM Real-Time PCR Detection System, Bio-Rad) using the GoTaq qPCR Mastermix (Promega, USA). Per reaction, 2 µl diluted cDNA were used. Three identically treated biological replicates were analysed. The thermocycler protocol was as follows: 3 min at 95°C, followed by 45 cycles of 10 s at 95°C, and 50 s at 58 or 60°C. For the generation of a standard curve, aliquots of the above-described cDNA samples of diverse tissue pools of the corresponding growth conditions were

used in a mixture. These mixtures were serially diluted (1 to 1, 1 to 2, 1 to 4, 1 to 8, 1 to 16, 1 to 32, and 1 to 64) with nuclease free water to generate standard curve templates and to determine PCR efficiencies for each primer pair. Assays producing PCR efficiencies > 80% < 115% were used for expression data analyses. Relative expression of *NIP5;1*, *NIP6;*1, and *BOR1* using the reference gene *AtEF1a* was determined by qPCR as previously published (Takano et al., 2006; Tanaka et al., 2008) using primers summarised in Table **S5**.

Phylogenetic and *in silico* parameter analysis

A maximum parsimony phylogenetic tree of the 185 Arabidopsis accessions was generated based on 214,051 Single Nucleotide Polymorphisms (SNPs; Horton et al., 2012, Atwell et al., 2010) using the R package "phangorn" (Schliep, 2011) and implementing the parsimony ratchet (Nixon, 1999). Accessions were manually assigned to one of 11 clades within the tree. B-efficient genotypes were plotted onto a geographic map using the function geom_map in the package "ggplot2", using the map "world_coordinates" as a background. Accessions were colour-coded to the phylogenetic clade determined in the previously described analysis. *k*-means clustering was used to group accessions by shared behavior across multiple shoot and root traits using the stats package in the software R. *k*-means clustering was based on a total of 38 traits, which were all ratios of the mean accession performance under -B compared to +B conditions. Of the 54 phenes retained from the automated phenotyping system, 11 were selected to represent shoot development for *k*-means clustering. These comprised projected leaf area, plant border length, branchpoint count, hsv h brown:green pixel ratio, hsv h yellow:green pixel ratio, hull area, hull length, hull points count, leaf count, mean leaf length, and mean leaf width. The remaining 43 phenes were excluded due to being aliased with another included phene (18), having limited variation either between genotypes or between growth conditions (15), concerning a non-plant relevant shape (e.g. rectangle area; 6), or contradicting with other measured phenes (4). Each phene was included at days 9, 12 and 16. The phene "hsv h brown:green pixel ratio" was not included at days 9 and 12, and "branchpoint count" was excluded at day 9, each due to missing data for most genotypes. Relative growth rates in the period from day 9-12 and day 13-16, as well as dry weight at experiment end, were additionally included, as were the four measured root

traits and DW of shoots from the *in vitro* experiment. The optimal number of *k*-clusters to use was determined using the fviz_nbclust function within the package "factorextra", considering both the "Elbow Method" and "Silhouette method". Twelve *k*-clusters were selected as the best representation of the data. Following assignment of accessions to a *k*-cluster, trait means for each *k*-cluster were recalculated, centred and scaled for generation of Fig **9**.

Genome-wide association analyses

The same set of 214,051 SNP markers as used for the phylogenetic analysis was used to conduct genome-wide association analyses. Analyses were performed using the R package "GAPIT" (version 3), implementing the the GWA method "FarmCPU" (PCA = 4; Liu et al., 2016). Manhattan plots were subsequently generated using R package "qqman" (Turner, 2018).

Proteoform Analysis

Amino acid sequence variations in the boron (B) transporters AtNIP5;1 (*At4g10380*), AtNIP6;1 (*At1g80760*) and AtBOR1 (*At2g47160*) of 1135 Arabidopsis accessions were analyzed using the SNP★ar tool (http://141.48.9.71/arabidopsis_thaliana#select_isoform). In the SNP★ar tool, the transcript identifier of the B transporters was selected and screened for SNPs in coding regions of the available accessions. Changes in the nucleotide sequences that result in non-synonymous amino acid substitutions in the three above-mentioned B transporters were identified and extracted. Their frequency was analysed across all 1135 accessions of the database, and the presence or absence of such changes among the accessions included in this study was assessed.

References

Atwell S, Huang Y, Vilhjálmsson B, Willems G, Horton M, Li Y, Meng D, Platt A, Tarone AM, Hu TT, Jiang R, Muliyati NW, Zhang X, Amer MA, Baxter I, Brachi B, Chory J, Dean C, Debieu M, de Meaux J, Ecker JR, Faure N, Kniskern JM, Jones JDG, Michael T, Nemri A, Roux F, Salt DE, Tang C, Todesco M, Traw MB, Weigel D, Marjoram P, Borevitz JO, Bergelson J, Nordborg M. (2010). Genome-wide association study of 107 phenotypes in *Arabidopsis thaliana* inbred lines. Nature 465, 627–631. https://doi.org/10.1038/nature08800

Eggert K, von Wirén N (2013). Dynamics and partitioning of the ionome in seeds and germinating seedlings of winter oilseed rape. Metallomics 5, 1316-1325. https://doi.org/10.1039/C3MT00109A

Horton MW, Hancock AM, Huang YS, Toomajian C, Atwell S, Auton A, Muliyati NW, Platt A, Sperone FG, Vilhjálmsson BJ, Nordborg M, Borevitz JO, Bergelson J (2012). Genome-wide patterns of genetic variation in worldwide *Arabidopsis thaliana* accessions from the RegMap panel. Nature Genetics 44, 212–216. https://doi.org/10.1038/ng.1042

Junker A, Muraya MM, Weigelt-Fischer K, Arana-Ceballos F, Klukas C, Melchinger AE, Meyer RC, Riewe D, Altmann T (2015). Optimizing experimental procedures for quantitative evaluation of crop plant performance in high throughput phenotyping systems. Frontiers in Plant Science 5, 770. https://doi.org/10.3389/fpls.2014.00770

Klukas C, Chen D, Pape JM (2014). Integrated Analysis Platform: An Open-Source Information System for High-Throughput Plant Phenotyping. Plant Physiology 165, 506-518. https://doi.org/10.1104/pp.113.233932

Liu X, Huang M, Fan B, Buckler ES, Zhang Z (2016). Iterative Usage of Fixed and Random Effect Models for Powerful and Efficient Genome-Wide Association Studies. PLOS Genetics 12, e1005767. https://doi.org/10.1371/journal.pgen.1005767

Nixon, KC (1999). The Parsimony Ratchet, a New Method for Rapid Parsimony Analysis. Cladistics 15, 407-414. https://doi.org/10.1111/j.1096-0031.1999.tb00277.x

Schliep KP (2011). phangorn: phylogenetic analysis in R. Bioinformatics 27, 592–593. https://doi.org/10.1093/bioinformatics/btq706

Shahzad Z, Kellermeier F, Armstrong EM, Rogers S, Lobet G, Amtmann A, Hills A (2018). EZ-Root-VIS: A Software Pipeline for the Rapid Analysis and Visual Reconstruction of Root System Architecture. Plant Physiology 177, 1368-1381. https://doi.org/10.1104/pp.18.00217

Tanaka M, Wallace IS, Takano J, Roberts DM, Fujiwara T (2008). NIP6;1 is a boric acid channel for preferential transport of boron to growing shoot tissues in *Arabidopsis*. The Plant Cell 20, 2860-2875. https://doi.org/10.1105%2Ftpc.108.058628

Takano J, Wada M, Ludewig U, Schaaf G, von Wiren N, Fujiwara T (2006). The *Arabidopsis* major intrinsic protein NIP5;1 is essential for efficient boron uptake and plant development under boron limitation. The Plant Cell 18: 1498–1509. https://doi.org/10.1105/tpc.106.041640

Tschiersch H, Junker A, Meyer RC, Altmann T (2017). Establishment of integrated protocols for automated high throughput kinetic chlorophyll fluorescence analyses. Plant Methods 13, 54. https://doi.org/10.1186/s13007-017-0204-4

Turner, SD (2018). qqman: an R package for visualizing GWAS results using Q-Q and manhattan plots. Journal of Open Source Software 3, 731. https://doi.org/10.21105/joss.00731
